# Genome-Scale CRISPRa Screen Identifies Novel Factors for Cellular Reprogramming

**DOI:** 10.1016/j.stemcr.2019.02.010

**Published:** 2019-03-21

**Authors:** Jian Yang, Sandeep S. Rajan, Mathias J. Friedrich, Guocheng Lan, Xiangang Zou, Hannes Ponstingl, Dimitrios A. Garyfallos, Pentao Liu, Allan Bradley, Emmanouil Metzakopian

**Affiliations:** 1 Wellcome Trust Sanger Institute, Genome Campus, Hinxton, Cambridgeshire CB10 1SA, UK; 2 UK Dementia Research Institute, Department of Clinical Neuroscience, Cambridge Biomedical Campus, University of Cambridge, Cambridge CB2 0AH, UK; 3 Cancer Research UK, Cambridge Institute, Li Ka Shing Centre, University of Cambridge, Cambridge CB2 0RE, UK; 4 School of Biomedical Sciences, Li Ka Shing Faculty of Medicine, Stem Cell and Regenerative Medicine Consortium, University of Hong Kong, Hong Kong, China

**Keywords:** CRISPR/Cas9, CRISPR activation, epiblast stem cells, reprogramming, genome-wide screen, activation screen, gain-of-function, CRISPR screen, reprogramming pathways, induced pluripotent stem cells

## Abstract

Primed epiblast stem cells (EpiSCs) can be reverted to a pluripotent embryonic stem cell (ESC)-like state by expression of single reprogramming factor. We used CRISPR activation to perform a genome-scale, reprogramming screen in EpiSCs and identified 142 candidate genes. Our screen validated a total of 50 genes, previously not known to contribute to reprogramming, of which we chose *Sall1* for further investigation. We show that *Sall1* augments reprogramming of mouse EpiSCs and embryonic fibroblasts and that these induced pluripotent stem cells are indeed fully pluripotent including formation of chimeric mice. We also demonstrate that *Sall1* synergizes with *Nanog* in reprogramming and that overexpression in ESCs delays their conversion back to EpiSCs. Lastly, using RNA sequencing, we identify and validate *Klf5* and *Fam189a2* as new downstream targets of *Sall1* and *Nanog*. In summary, our work demonstrates the power of using CRISPR technology in understanding molecular mechanisms that mediate complex cellular processes such as reprogramming.

## Highlights

• Genome-scale CRISPRa screen in mouse EpiSCs identifies novel reprogramming factors
• 50 novel genes, including *Sall1* and *Fam189a2*, identified to mediate reprogramming
• *Sall1* synergizes with *Nanog* to increase reprogramming efficiency in EpiSCs and MEFs
• RNA-seq provides insight into downstream pathways of *Sall1* and *Nanog*-mediated reprogramming

## Graphical Abstract

**Figure:**
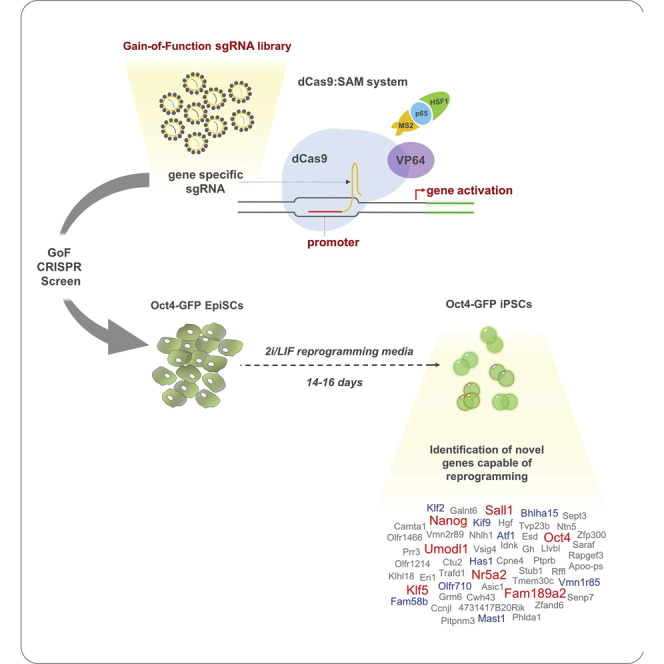

## Teaser

In this study, Metzakopian and colleagues use CRISPR activation to perform a genome-scale reprogramming screen on mouse epiblast stem cells identifying 50 novel candidates. In addition, the authors provide evidence of *Sall1* being a potent reprogramming gene, capable of synergizing with *Nanog*. Lastly, using RNA-seq, the authors provide insight into potential downstream targets of *Sall1* and *Nanog*.

## Introduction

The ability of pluripotent stem cells (PSCs) to self-renew and their potential to differentiate into multiple cell types makes them useful for clinical applications (Martello and Smith, 2014). PSCs can either be derived from early embryos or be induced (iPSCs) by reprogramming somatic cells with Yamanaka factors, i.e., *Oct4*, *Sox2*, *c-Myc*, and *Klf4* among other transcription factors, mRNAs, microRNAs, and small molecules (Hou et al., 2013, Sandmaier and Telugu, 2015, Takahashi and Yamanaka, 2006, Warren et al., 2010). During early mouse embryo development, at least two types of PSCs can be derived, naive embryonic stem cells (ESCs) from the inner mass of the blastocyst and primed post-implantation epiblast stem cells (EpiSCs) (Nichols and Smith, 2009, Tesar et al., 2007). While both have the potential to differentiate into multiple lineages, only ESCs can contribute extensively to chimeras, showing unbiased developmental potential. Both ESCs and EpiSCs express major pluripotent transcription factors such as *Oct4* and *Sox2* at similar levels. In EpiSCs, however, reduced expression of pluripotency-associated factors such as *Rex1* and *Klf4* and elevated levels of early differentiation markers such as *Fgf5*, *Gata6*, and *Otx2* indicate their restricted developmental potential. Interestingly, EpiSCs cultured in fully defined ESC medium (with inhibition of *MAPK* and *GSK3* and supplementation with LIF; hereafter 2i/LIF medium) can be reprogrammed into ESCs by overexpressing only a single gene––such as *Nanog*, *Klf4*, or *Nr5a2* (Guo and Smith, 2010)––making them an ideal model system for genetic screens.

Recently, CRISPR/Cas9 has gained importance by achieving simple, precise, and rapid editing of the genome, enabling large-scale experiments such as genetic screening. While the RNA-programmable (single guide RNA [sgRNA]) endonuclease Cas9 is used to induce double-strand breaks in defined genomic locations, its catalytically dead variant (dCas9) can be fused with transcriptional activators and directed toward promoter regions to increase gene expression (CRISPR activation, CRISPRa) (Doudna and Charpentier, 2014, Gaj et al., 2013).

Genome-wide screening is a powerful unbiased approach to discover genes and pathways that underlie biological processes. To date, identification of key transcription factors and epigenetic modifiers within naive and primed PSCs has been investigated by employing either gain-of-function (GoF) screens using cDNA libraries and *PiggyBac* transposons or loss-of-function screens using RNA interference (Gayle et al., 2015, Hu et al., 2009, Pritsker et al., 2006).

Here, we describe the development and application of a genome-scale CRISPRa screen to identify genes that contribute to mouse EpiSC reprogramming. We show that our screening approach not only detects established reprogramming factors such as *Oct4* and *Nanog*, but also identifies previously unreported candidate genes capable of reprogramming. We focus on the role of *Sall1*, a transcription factor belonging to the Spalt-like gene family, which has been implicated in cellular reprogramming in a number of studies but has not been sufficiently investigated (Basta et al., 2017, Gaspar-Maia et al., 2013, Mansour et al., 2012). Our work substantiates *Sall1* as a potent reprogramming gene candidate by demonstrating its ability to reprogram EpiSCs and mouse embryonic fibroblasts (MEFs) to iPSCs. In addition, we show that *Sall1* may exert its functions by interacting synergistically with *Nanog* to reprogram cells to ground state pluripotency.

## Results

### GoF CRISPRa Screen Identifies Reprogramming Genes

Initially, we sought to determine the optimal Cas9 transactivation system, as several variants have been published (Chavez et al., 2016, Konermann et al., 2015, Tanenbaum et al., 2014). To that end, we created *PiggyBac*-transposable (Yusa et al., 2011) expression vectors with a Blasticidin-mCherry cassette for four different dCas9-CRISPRa systems: dCas:VP160, dCas9:SunTag, dCas9:VPR, and dCas9:SAM (Figure S1).

Furthermore, we designed a versatile sgRNA expression construct (pKLV-PB-U6-gRNA-PGK-Puro-T2A-TagBFP) (Metzakopian et al., 2017) with a selectable and a fluorescent marker (puromycin and BFP, Figure S1), which can be stably integrated into target genomes as lentivirus or via *PiggyBac*-mediated transposition (Yusa et al., 2011).

**Figure 1:**
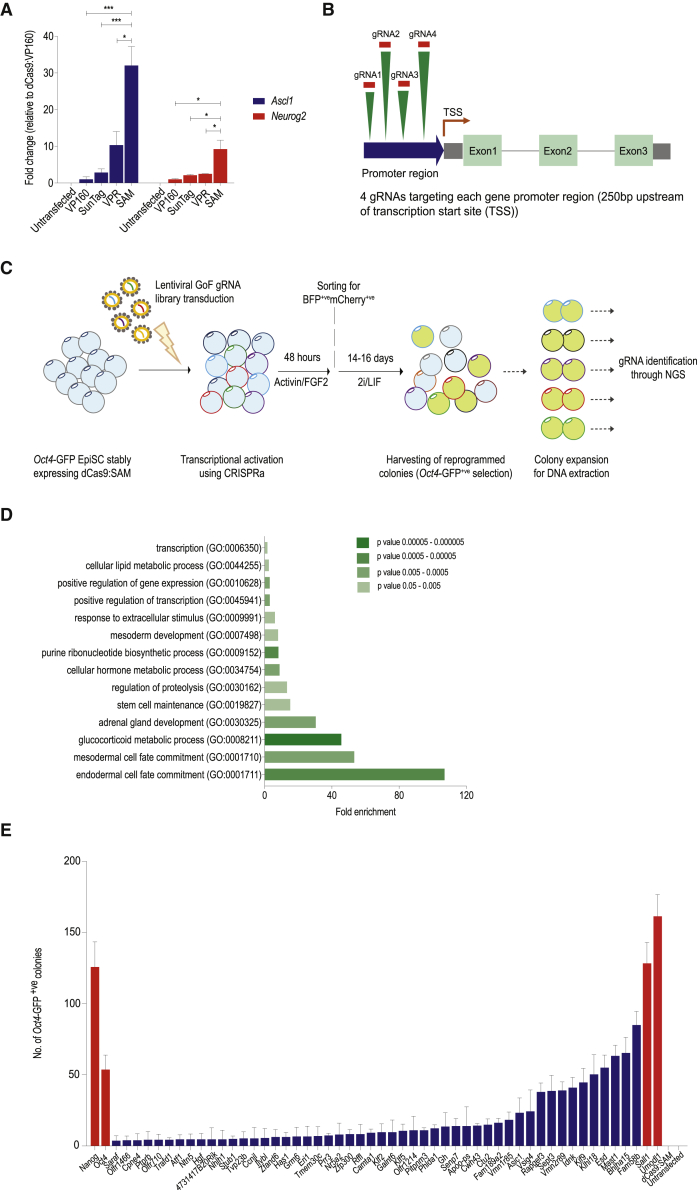
GoF EpiSC Reprogramming Screening with CRISPRa and sgRNA Library (A) Activation of *Ascl1* and *Neurog2* in HEK293 cells. Cells were transfected with one sgRNA per target and four different dCas9 versions. qRT-PCR normalized to *Gapdh*, fold change relative to dCas9:VP160 (mean of experimental triplicates ± SD, ^∗^p < 0.05; ^∗∗∗^p < 0.001). (B) sgRNA design targeting gene promoters in the murine genome. (C) Screening strategy in *Oct4*-GFP EpiSCs stably expressing dCas9:SAM, lentiviral transduction (MOI = 0.3) of the sgRNA library. Reprogramming in 2i/LIF for 14–16 days, after sorting for transduced cells. NGS identified candidate sgRNAs in *Oct4*-GFP^+ve^ iPSC colonies. (D) GOTOOLBox analysis of 142 genes identified in GoF screening. Pathways with fold change compared with reference; colors indicate p values. (E) Validation of 54 genes including *Nanog* and *Oct4* in dCas9:SAM-*Oct4*-GFP EpiSCs with single sgRNAs (*Oct4*-GFP^+ve^ iPSC colonies, mean of 3 independent experiments ± SD). See also Figures S1 and S2 and Tables S5, S6, and S7.

We directed single sgRNAs guides against the promoter region of *Ascl1* and *Neurog2*, genes with low baseline expression, in HEK293 cells. After stable integration of dCas9-CRISPRa and the sgRNA vectors via transposition and antibiotic selection, qRT-PCR revealed that dCas9:SAM achieved the highest overexpression of both target genes and thus was chosen for all subsequent experiments (Figure 1A).

To perform a genome-scale activation screen, we designed a pooled library of 87,863 sgRNAs targeting a 250-bp region upstream of the transcription start site (TSS) of 19,994 genes with an average of 4 guides each (Figure 1B; Table S5).

We decided to use EpiSC derived from *Oct4*-GFP reporter transgenic mice as they have been used for this purpose before (Yang et al., 2010). Characteristically for EpiSCs these cells already exhibit a baseline *Oct4* (and therefore GFP) expression. However, only cells successfully reprogrammed to the naive pluripotent state are able to maintain and increase *Oct4* expression upon plating in aforementioned 2i/LIF medium. Thus, successfully reprogrammed *Oct4*-GFP EpiSCs can be identified by their strong GFP expression (Figure S2A) and the characteristic ESC-like morphology, and grow as distinct colonies, whereas EpiSCs failing to reprogram either detach and die or differentiate.

We stably integrated dCas9:SAM into *Oct4*-GFP EpiSCs via *PiggyBac* transposition and then transduced 100 × 10^6^ dCas9:SAM-expressing EpiSCs with our library at a MOI of 0.3 (Figure S2B). Two days later, we used fluorescence-activated cell sorting 10 × 10^6^ to successfully transduce cells by BFP expression, giving a library coverage of around 114-fold. These BFP^+ve^ cells were seeded in 2i/LIF medium to select for reprogramming cells. After 14–16 days of culture in 2i/LIF, 480 GFP^+ve^ colonies were harvested for expansion (Figure 1C). Next-generation sequencing revealed 146 sgRNAs targeting 142 different genes (Table S6). These included known reprogramming factors *Nanog* (Mitsui et al., 2003), *Klf2* (Qiu et al., 2015), and *Nr5a2* (Guo and Smith 2010), confirming the specificity of the screen.

GOTERM analysis (Castro et al., 2011) on these 142 genes identified an enrichment in pathways related to transcriptional activation, expression of various transcription factors and enrichment toward stem cell maintenance (Figure 1D; Table S6).

To validate these candidate genes individually, we chose the highest performing sgRNA for each from the library, including *Nanog* as a positive control and again transduced dCas9:SAM-expressing *Oct4*-GFP EpiSCs. We expected the validation rate to be no higher than 50%, as small-scale single colony sub-sampling showed an average of two sgRNAs present in most colonies (data not shown), where one sgRNA presumably acts as the driver responsible for reprogramming, while the other is co-amplified as a passenger. As before, GFP^+ve^ ESC-like colonies could be observed for *Oct4*, *Nanog*, and 52 of the candidate genes, resulting in a 36% validation rate (Table S7). The efficiency of reprogramming was gene dependent ranging from 5 to 165 colonies per 1 × 10^6^ cells transfected (Figure 1E). Among the genes with the highest colony counts were positive controls *Nanog* and *Oct4*, as well as transcription factors *Klf2* and *Nr5a2* with a known role in reprogramming, confirming the validity of our CRISPRa approach.

### Gene Dosage Is Critical for *Oct4*-Mediated Reprogramming

**Figure 2:**
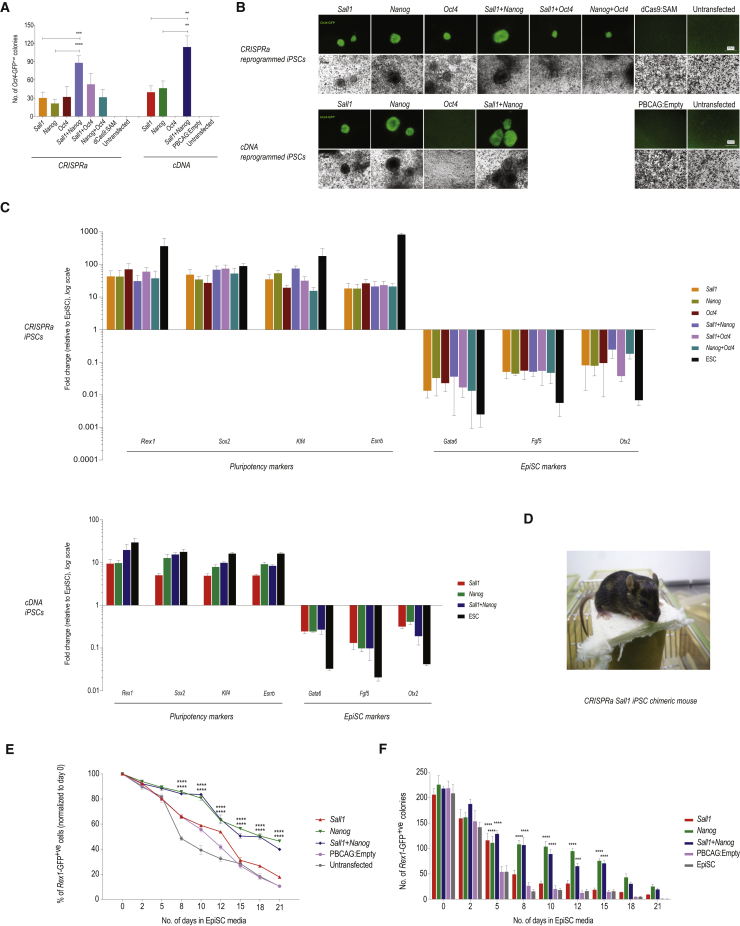
*Sall1* and *Nanog* Reprogram EpiSCs and Influence ESC Differentiation (A) Reprogramming efficiencies of *Sall1*, *Nanog*, and *Oct4* in *Oct4*-GFP EpiSCs stably transfected with CRISPRa or cDNA (*Oct4*-GFP^+ve^ colonies, mean of 3 independent experiments ± SD, ^∗∗^p < 0.01; ^∗∗∗^p < 0.001; ^∗∗∗∗^p < 0.0001). (B) Morphology of *Oct4*-GFP^+ve^ colonies at day 20 in 2i/LIF is similar to ESC colonies. No colonies were observed in untransfected or mock transfected EpiSCs. (C) qRT-PCR expression profiles of pluripotency markers and EpiSC markers in iPSC colonies normalized to *Gapdh* and relative to EpiSCs (mean of 3 independent experiments ± SD). (D) Chimeric mouse produced with CRISPRa *Sall1*-induced PSCs injected into C57B/6 blastocyst. (E) Flow cytometric analysis of *Rex1*-GFP^+ve^ cells cultured in EpiSC medium at the timepoints indicated. Cells were stably transfected with *Sall1* or *Nanog* cDNA, or empty vector and cultured in EpiSC medium (mean of 3 independent experiments ± SD, ^∗∗∗∗^p < 0.0001 versus PBCAG:Empty). (F) Number of *Rex1*-GFP^+ve^ ESC colonies recovered after ESCs were converted in EpiSC medium at indicated timepoints (600 cells plated at time point zero; mean of 3 independent experiments ± SD, ^∗∗∗^p < 0.001; ^∗∗∗∗^p < 0.0001 versus PBCAG:Empty). See also Figure S3 and Table S2.

We observed that CRISPRa-mediated induction of the pluripotency marker *Oct4* produced a significant number of ESC-like colonies, contradicting previous studies showing that cDNA-mediated *Oct4* expression is inefficient in EpiSC reprogramming (Guo and Smith, 2010, Niwa et al., 2000). Indeed, we were unable to generate any iPSC colonies in our EpiSCs with *Oct4* cDNA, while CRISPRa achieved robust reprogramming (Figure 2B).

We speculated that gene dosage might be the underlying issue and compared endogenous *Oct4* induction in EpiSCs with exogenous overexpression in more detail: compared with ESCs, CRISPRa-mediated induction of *Oct4* mRNA achieved roughly half the physiological expression level, while exogenous *Oct4* cDNA slightly surpassed it (Figure S2C). Doxycycline (Dox) titration of tet-inducible *Oct4* resulted in amounts comparable with *Oct4* cDNA and only very low concentrations of Dox gave levels similar to CRISPRa. Nevertheless, all cDNA-mediated overexpression conditions still failed to reprogram. On the protein level, all *Oct4* cDNA conditions produced disproportionally higher amounts than expected from the mRNA levels (Figure S2D, top panel). CRISPRa, on the other hand, achieved *Oct4* protein expression similar to that in ESCs (Figure S2D, bottom panel). We suspect that differences in mRNA stability are the cause, as CRISPRa-driven endogenous mRNA should be physiologically regulated, while exogenous mRNA could be more stable due to differing polyadenylation.

The importance of physiological expression levels agrees with our observation that, although our screening library contained an average of four sgRNAs per gene, almost all candidate genes from our screen were derived from only one specific sgRNA per target. Indeed, sgRNAs showed vastly different activities in a distribution that suggests a dependency on the distance of the sgRNA to the TSS (Figure S2E). This is also supported by a recent report (Liu et al., 2018a), which shows proof-of-principle MEF reprogramming using CRISPRa. In their experiments, only sgRNAs targeting the *Oct4* promoter in very specific locations (−71 and −127 bp from TSS) achieved activation sufficient for reprogramming, while in our experiments an sgRNA −101 bp from the TSS was successful.

### *Sall1* Facilitates EpiSC Reprogramming in Cooperation with *Nanog*

*Umodl1* and *Sall1* were the two most potent validated candidates from our screen. We confirmed that *Umodl1* upregulates *Lifr*, *Essrb*, *Nanog*, and *Sox2*, and downregulated *Tgfbr1* as would be expected in iPSC reprogramming when medium was switched from EpiSC to 2i/LIF (Figure S2F). *Sall1*, a member of the Spalt-family of transcription factors, has been reported to cooperate with *Nanog* to promote the maintenance of ESC state (Karantzali et al., 2011, Novo et al., 2016) and to play an important role in reprogramming and ESC differentiation (Basta et al., 2017, Mansour et al., 2012). However, the downstream targets of *Sall1* involved in reprogramming have not been sufficiently explored. Having found that *Sall1* is also able to independently reprogram EpiSCs, we set to investigate the underlying mechanisms.

First, we asked whether *Sall1* and *Nanog* also act synergistically in EpiSC reprograming by overexpressing them individually and in combination in *Oct4*-GFP EpiSCs. We performed these experiments both with CRISPRa as well as cDNA-mediated overexpression, and also verified that the observed activity of *Sall1*-specific sgRNA was not due to cross-reactivity with *Sall4*, a known pluripotency factor (Figure S2G).

Three days after transfection, qRT-PCR showed a 2.5- to 3.5-fold increase in expression of *Sall1*, *Nanog*, and *Oct4* mediated by CRISPRa and a 10- to 20-fold increase in expression through cDNA (Figure S3A). Co-expression of *Sall1* and *Nanog* resulted in a marked increase in *Oct4*-GFP^+ve^ ESC-like colony numbers (Figures 2A and 2B). As above, *Oct4* induction via CRISPRa successfully reprogrammed EpiSCs (but not cDNA overexpression), without showing significant synergy with either *Sall1* or *Nanog*. Pluripotency markers examined by qRT-PCR (*Rex1*, *Sox2*, *Klf4*, and *Essrb*) were markedly increased; concordantly, EpiSC markers *Gata6*, *Fgf5*, and *Otx2* showed decreased expression (Figure 2C). *Sall1* reprogrammed EpiSCs (MF1 and C57BL/6 background) contributed significantly to chimeras when injected into C57BL/6 blastocysts (Figures 2D and S3E).

To exclude the possibility that the baseline GFP expression of the *Oct4*-GFP reporter EpiSCs might skew the correct identification of successfully reprogrammed EpiSCs, we repeated these experiments with *Nanog*-GFP reporter EpiSCs (Yang et al., 2010), which show strong GFP expression on successfully entering the naive ESC state, but virtually none in the primed EpiSC state (Guo and Smith, 2010). Both gene induction using CRISPRa and overexpression via cDNA confirmed the reprogramming capability of *Sall1* alone and in synergy with *Nanog* (Figures S3B–S3D). Notably, colony formation assays in 2i/LIF recapitulated the results obtained with *Oct4*-GFP EpiSCs and the reprogramming kinetics as measured in time course experiments were comparable between the two reporter cell lines (Figure S3F).

### *Sall1* and *Nanog* Delay Differentiation of ESCs into EpiSCs

ESCs readily differentiate into EpiSCs in culture medium containing the EpiSC self-renewal factors Activin and fibroblast growth factor 2 (FGF2) (Guo et al., 2009). To investigate whether higher levels of *Sall1* and *Nanog* can delay this conversion we generated stable cDNA transfectants in *Rex1*-GFP ESCs (Wang et al., 2011). We cultured the cells in EpiSC media and quantified the *Rex1*-GFP^+ve^ population as a measure of cells remaining in the ESC ground state in a 21-day time course. *Nanog* and *Sall1*+*Nanog* maintained a significantly higher proportion of GFP^+ve^ cells than *Sall1* (Figure 2E). The expression of naive pluripotency and EpiSC markers analyzed by qRT-PCR followed a similar pattern (Figures S3G–S3I), although *Sall1* delayed upregulation of differentiation markers *Fgf5* and *Otx2*. Concordantly, when plated in 2i/LIF medium, *Nanog* and *Sall1*+*Nanog* overexpressing cells retained the ability to form ESC colonies through most of the time course, and *Sall1* preserved colony formation capacity until after 6 days (Figure 2F). While *Sall1* might not have the same capacity as *Nanog* to keep the ESC ground state, it may confer a longer “formative state” (Smith, 2017) during conversion.

### *Sall1* Promotes MEF Reprogramming and Works Synergistically with *Nanog*

**Figure 3:**
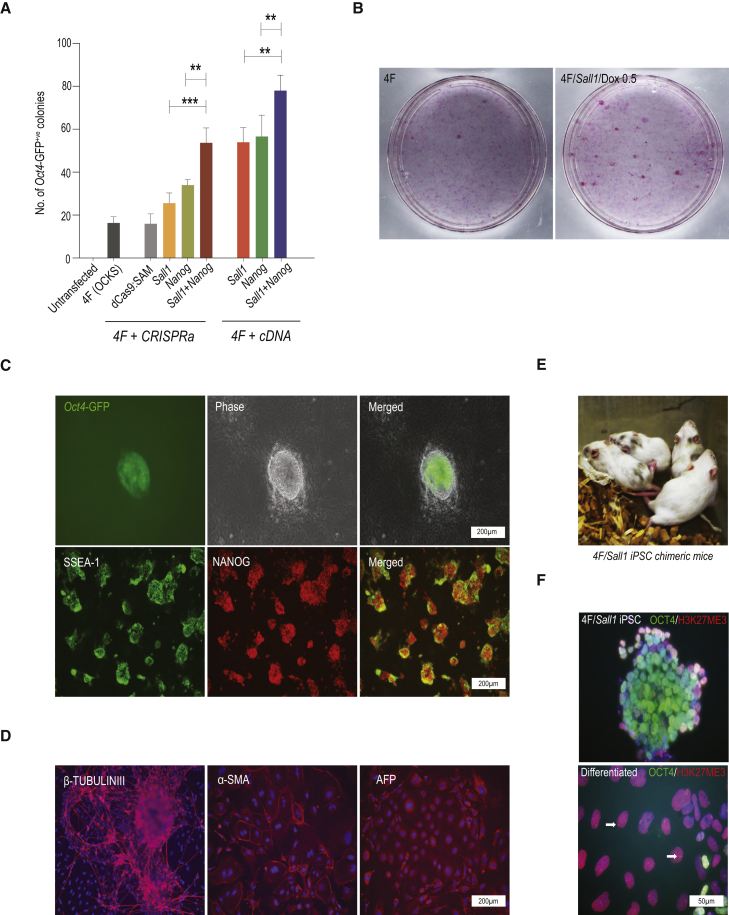
CRISPRa Gene Induction and cDNA-Mediated Overexpression of *Sall1*, *Nanog* Reprogrammed MEF to iPSCs (A) (4F + CRISPRa) MEFs stably transfected with CAG4F and gRNAs against *Sall1*/*Nanog*/*Sall1*+*Nanog* in ESC medium (*Oct4*-GFP^+ve^ colonies after 18 days; mean of 3 independent experiments ± SD, ^∗∗^p < 0.01; ^∗∗∗^p < 0.001) (4F + cDNA) MEFs stably transfected with TRE4F, TRENanog, and TRESall1 (all co-transfected with PBEF-1αTet3G), induced with 0.5 μg/mL Dox for 12 days and counted on day 18 (mean of 3 independent experiments ± SD, ^∗∗^p < 0.01). (B) Alkaline phosphatase-positive (AP^+ve^)-stained ESC colonies reprogrammed from MEFs by 4F alone and in combination with *Sall1* (induced with Dox at 0.5 μg/mL). (C) iPSCs reprogrammed from C57B/6J MEF with 4F/Sall1. *Oct4*-GFP expression and ESC-like morphology (upper panel), immunofluorescent staining for pluripotency markers SSEA-1 and NANOG (lower panel). (D) *In vitro* differentiation of C57B/6 MEF reprogrammed iPSCs with 4F/Sall1; neuronal differentiation in N2B27 (immunofluorescence for β-tubulin III^+^); mesoderm and endoderm differentiation in M10 (alpha smooth muscle actin [α-SMA] and alpha fetoprotein [AFP] antibody staining). (E) Chimeric mice produced with 4F/Sall1-iPSCs injected into CD1 blastocysts. (F) Inactivation of X chromosomes in female 4F/Sall1-iPSCs (co-immunostaining for H3K27me3 and *Oct4*. Arrows indicate H3K27me3 foci). See also Figure S4 and Tables S2 and S3.

To test whether *Sall1* enhances somatic cell reprogramming, we stably transfected *Oct4*-GFP reporter MEFs (*Oct4*-GFP-MEFs) with the Yamanaka factors (CAG4F, Figure S1), dCas9:SAM and sgRNAs against *Sall1* and/or *Nanog*. In ESC media, *Sall1* sgRNA transfected MEFs produced a significantly higher number of *Oct4*-GFP^+ve^ and alkaline phosphatase-positive (AP^+ve^) colonies (Figures 3A and S4A) with ESC-like morphology (Figure S4B) than CAG4F alone, mirroring the results obtained from EpiSC reprogramming, including synergy between *Sall1* and *Nanog*.

To examine the dynamics of MEF reprogramming, we co-transfected *Oct4*-GFP-MEFs with tet-inducible *Sall1* (TRESall1, Figure S1; Table S2) and CAG4F, and induced expression with three concentrations of Dox (0.1, 0.5, and 1.0 μg/mL) to find a suitable concentration to mediate reprogramming in ESC medium. After 18 days, Dox concentrations of 0.5 or 1.0 μg/mL resulted in a significant 2- to 3-fold increase in *Oct4*-GFP^+ve^ and AP^+ve^ colonies (Figures 3B and S4C) and we chose 0.5 μg/mL Dox for all subsequent experiments. To determine the active window for *Sall1*, we induced expression at 0, 2, 4, 6, 8, 10, and 12 days of reprogramming and found that only activation during the first 4 days resulted in higher reprogramming efficiency (Figure S4D).

As *Nanog* has been reported to promote MEF reprogramming (Theunissen et al., 2011) we tested for synergy with *Sall1* by transfecting MEFs with tet-inducible Yamanaka factors, *Nanog* and *Sall1* (TRE4F, TRENanog, and TRESall1). Indeed, co-expression of *Sall1*+*Nanog*/4F led to a 1.5-fold increase in colony number compared with either factor alone (Figures 3A and S4A).

The *Sall1*-iPSCs derived from these experiments were morphologically similar to ESCs with a compact dome-like structure and *Oct4*-GFP expression. Immunofluorescent staining of these iPSCs showed protein expression of the ESC-markers SSEA-1 and NANOG (Figure 3C). In differentiation medium (DMEM/10% fetal calf serum [FCS]) or N2B27 medium (Ying et al., 2003), these iPSCs exited ground state pluripotency and differentiated into mesoderm, endoderm, and ectoderm lineages as confirmed by immunofluorescent staining for expression of smooth muscle actin, alpha fetoprotein, and β-tubulin III (Figure 3D). In addition, when we injected these iPSCs into blastocysts, live chimeras were born (Figure 3E), confirming the pluripotency of these *Sall1*-iPSCs.

Female mESCs have two activated X chromosomes when maintained at ground state (Lessing et al., 2013) and randomly inactivate one of them once they undergo differentiation. Staining with anti-H3K27me3 antibody detects this event as foci on the inactivated X chromosome (Silva et al., 2008). We derived iPSCs from female MEFs by co-transfecting with 4F/*Sall1* as before and then differentiated them in DMEM/10% FCS for 5 days. Loss of *Oct4* expression demonstrated successful differentiation and the presence of H3K27me3 foci indicated X chromosome silencing. In contrast iPSC cultured in 2i/LIF strongly expressed *Oct4* protein and lacked any H3K27me3 foci (Figure 3F).

Together, this demonstrates that *Sall1* can enhance 4F-driven somatic cell reprogramming and that 4F/*Sall1* reprogrammed iPSCs are naive and pluripotent.

Yamanaka factors *Oct4*, *Sox2*, and *Klf4* are essential and sufficient for reprogramming, albeit at a lower efficiency than in conjunction with *c-Myc*; all three can be replaced by other transcription factors or small molecules such as *Gata3* (Shu et al., 2013) or valproic acid (Biswas and Jiang, 2016, Huangfu et al., 2008). However, in co-transfection experiments, *Sall1* was unable to substitute for any of the factors in MEFs (Figures S4E–S4G).

### RNA Sequencing Identifies Potential Mechanisms of Cellular Reprogramming Mediated by *Sall1* and *Nanog*

**Figure 4:**
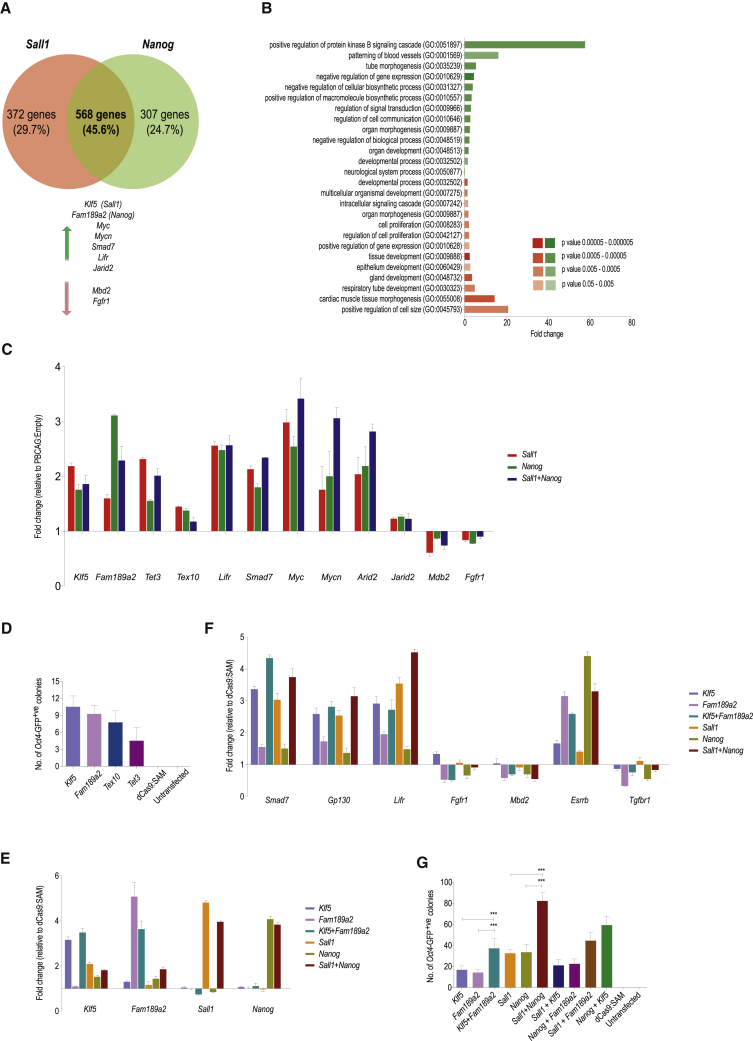
RNA-Seq Identifies Potential Mechanisms of Reprogramming Mediated by Sall1 and Nanog (A) Venn diagram of genes being differentially expressed in *Sall1* and *Nanog* overexpressing cells (cutoff p_adj_ < 0.001). Upregulated (green arrow) and downregulated (red arrow) genes for further experiments were chosen from the overlap between *Sall1* and *Nanog*, except *Klf5* and *Fam189a2*. (B) GOTOOLBox analysis of common regulated genes (fold change compared with reference, colors indicate p values). (C) qRT-PCR validations of RNA-seq (24 h after transfection, normalized to *Gapdh* and relative to PBCAG:Empty; mean of 3 independent experiments ± SD). (D) Reprogramming of *Oct4*-GFP EpiSCs via CRISPRa-mediated gene induction of *Klf5*, *Fam189a2*, *Tex10*, and *Tet3* (*Oct4*-GFP^+^ colonies after 2i/LIF for 20 days; mean of 3 independent experiments ± SD). (E) qRT-PCR for *Klf5*, *Fam189a2*, *Sall1*, and *Nanog* after CRISPRa-mediated induction of *Klf5* and *Fam189a2* (flow-sorted for sgRNA expression after 24 h, normalized to *Gapdh* and relative to dCas9:SAM; mean of 3 independent experiments ± SD). (F) qRT-PCR expression levels of key regulators in JAK/STAT3 and TGF-β signaling (flow-sorted for sgRNA expression 24 h after changing to 2i/LIF media, 48 h after transfection, normalized to *Gapdh* and relative to dCas9:SAM; mean of 3 independent experiments ± SD). (G) Reprogramming of *Oct4*-GFP EpiSCs via CRISPRa-mediated gene induction of *Klf5*, *Fam189a2*, *Sall1*, and *Nanog* (*Oct4*-GFP^+ve^ colonies after 20 days in 2i/LIF; error bars represent mean of 3 independent experiments ± SD). See also Figure S4 and Tables S2 and S8.

We performed RNA sequencing (RNA-seq) for *Oct4*-GFP EpiSCs overexpressing *Sall1* and/or *Nanog* via cDNA for 24 h. Our analysis identified 372 genes differentially expressed specific to *Sall1*-transfected cells, and 307 genes specific to *Nanog*. We observed a large overlap of 568 genes (45%) between both sets (Figure 4A; Table S8) and GOTERM analysis (Castro et al., 2011) revealed that they are involved in a number of developmental processes and signaling cascades (Figure 4B; Table S8).

Among those commonly regulated genes were *Myc*, *Mycn*, *Tet3*, *Tex10*, *Jarid2*, *Fgfr1*, *Mbd2*, *Lifr*, and *Smad7* (Figure 4A) which have previously been implicated in the promotion of cellular reprogramming or inhibition of ESC differentiation (Li et al., 2016, Iseki et al., 2016, Bagci and Fisher, 2013, Fidalgo et al., 2016, Jinek et al., 2012, Niwa et al., 1998, Hall et al., 2009, Festuccia et al., 2017). Upregulation of *Lifr* and downregulation of *Fgfr1* is expected in EpiSC reprogramming and validates our RNA-seq and qRT-PCR data (Figure 4C). Furthermore, we found 215 genes that were only regulated when *Sall1* and *Nanog* were overexpressed together (Figure S4H; Table S8), such as *Dnmt3c* and *Hdac9*, reported to be involved in the epigenetic regulation of male germ cell maintenance (Barau et al., 2016) and muscle differentiation (Mihaylova and Shaw, 2013), respectively; as well as a modest upregulation of *Utf1*, a transcription factor known to synergize with the Yamanaka factors in reprogramming (Zhao et al., 2008).

We had already independently identified the genes *Klf5* and *Fam189a2* in our GoF screen (Figure 1E; Table S5) and RNA-seq showed them to be potentially regulated by *Sall1* and *Nanog*, respectively. We validated the RNA-seq results with qRT-PCR and found a good correlation between both methods (Figures 4C and S4I). While *Klf5* narrowly failed the stringent p value cutoff for the RNA-seq results in *Nanog* overexpressing cells, qRT-PCR indicated that *Klf5* may be regulated by *Nanog* as well, albeit to a lesser extent than by *Sall1*. *Fam189a2* on the other hand seemed to be regulated significantly stronger by *Nanog* than *Sall1*. When we co-expressed *Sall1* and *Nanog*, we did not observe a significant synergistic increase of expression for these downstream targets (Figure 4C); we did, however, for the genes *Myc*, *Mycn* (Chappell and Dalton 2013), and *Arid2*, all of which have been shown to play a role in reprogramming and chromatin remodeling (Awe and Byrne, 2013, Singhal et al., 2010).

We used CRISPRa to induce expression of *Klf5*, *Fam189a2*, *Tex10*, and *Tet3* in *Oct4*-GFP EpiSCs and found that all were able to augment reprogramming into iPSCs (Figure 4D). Reprogramming by *Fam189a2* occurred in 10 days, while *Klf5*, *Tex10*, and *Tet3* required between 14 and 20 days. In all cases, the number of reprogrammed colonies was significantly lower compared with *Sall1* or *Nanog* (Figure 4G), which may indicate that multiple downstream targets of *Sall1* and *Nanog* participate in reprogramming.

We tested the regulatory relationship between *Sall1+Nanog* and *Klf5*+*Fam189a2* by transfecting EpiSCs with CRISPRa for *Klf5* and *Fam189a2*. Transcription increased significantly for *Klf5* and *Fam189a2*, but not for *Sall1* and *Nanog*, indicating *Klf5* and *Fam189a2* are downstream targets (Figure 4E). qRT-PCR for some of the key genes differentially regulated in the RNA-seq data showed that both *Sall1* and *Klf5* upregulated *Smad7*, *Gp130*, and *Lifr*, suggesting the repression of transforming growth factor β (TGF-β) signaling and activation of Jak/Stat3 signaling. *Nanog* and *Fam189a2* on the other hand downregulated *Fgfr1*, *Tgfr1*, and *Mbd2* and upregulated *Esrrb* expression, indicating the repression of FGF and TGF-β signaling, inhibition of epigenetic repression and promotion of self-renewal and pluripotency (Figure 4F). Functionally, co-activation of both *Klf5* and *Fam189a2* generated significantly more *Oct4*-GFP^+ve^ colonies than either gene alone. As expected, co-activation of either *Sall1* and its downstream target *Klf5* or *Nanog* and its downstream target *Fam189a2* showed no synergistic effects in *Oct4*-GFP^+ve^ colony production, whereas co-activation of either *Sall1* and *Fam189a2* or *Nanog* and *Klf5* did, although less than *Sall1* and *Nanog* co-activation. These results suggest that *Klf5* and *Fam189a2* are situated downstream of *Sall1* and *Nanog*, respectively, and can synergize as well (Figure 4G).

## Discussion

To date few CRISPR activation screens have been performed (Bester et al., 2018, Heaton et al., 2017, Liu et al., 2018b) using previously established GoF libraries (Kampmann, 2018, Konermann et al., 2015). However, none of them targeted stem cell reprogramming and, while some recent publications have used CRISPRa in this field of research, they have been restricted to a few genes to demonstrate proof-of-concept (Guo et al., 2017, Liu et al., 2018a, Weltner et al., 2018).

Our present study shows that a genome-scale CRISPRa screen, in conjunction with an experimental model such as EpiSCs, in which a single overexpressed gene may mediate reprogramming to pluripotency, is a powerful tool for gene discovery. We identified 142 candidate reprogramming factors, among them *Nanog*, known to reprogram EpiSCs to iPSCs (Silva et al., 2009), validating our screen. Similarly, we found the Yamanaka factor *Oct4* (Takahashi and Yamanaka, 2006), which is critical for the maintenance of ESCs and differentiation (Niwa et al., 2000). Curiously, CRISPRa-induced *Oct4* readily and robustly reprogrammed EpiSCs into iPSCs, while overexpression via cDNA failed in our experiments and those of others (Guo and Smith, 2010). We reason that gene dosage is one critical aspect to explain this behavior and that excessive levels of *Oct4* can be detrimental to pluripotency, which tallies with previous studies suggesting that artificially reduced *Oct4* levels maintain ESCs in a robust pluripotent state, whereas wild-type levels enable differentiation (Gao et al., 2013, Karwacki-Neisius et al., 2013, Radzisheuskaya et al., 2013).

The important implication for CRISPRa-mediated screens is that tiled sgRNAs in regulatory regions of genes can, as we and others show (Liu et al., 2018a), provide a variety of expression levels unachievable with exogenous cDNA, giving a higher probability of matching the physiological gene dosage. Conceivably, the choice of the CRISPRa system may well influence the outcome of a screen and repeating our screen with a different CRISPRa system at lower activation efficiencies than SAM could produce a non-redundant list of candidate genes.

While the positional aspect of sgRNA efficiency certainly serves to explain why most of our candidate genes were only identified by a single sgRNA in our screen, we also acknowledge that reprogramming is inherently a very inefficient process and, thus, a very large initial cell number may be required to cover a genome-wide library deeply enough to give a sufficient number of cells a chance to gain pluripotency. While we performed our GoF screen with 10 × 10^6^ library-transduced cells (library coverage 114×), a deeper coverage or a more focused library promises to uncover reprogramming candidates the present screen might have missed.

Our screen identified *Sall1*, a member of the Spalt-like gene family, as a potent EpiSC reprogramming factor. *Sall1* and *Sall4* have been implicated in the establishment of pluripotency (Gaspar-Maia et al., 2013) in studies showing that the action of demethylase *Utx* on *Sall1* and *Sall4* is required to enable MEF reprogramming (Mansour et al., 2012). Furthermore, it has been demonstrated that *Sall4* activates *Oct4* expression while *Sall1* is a direct binding partner of *Nanog* (Karantzali et al., 2011, Zhang et al., 2006) and has been suggested to be required in *Nanog*-mediated open heterochromatin maintenance within ESCs and EpiSCs (Novo et al., 2016). So far, it is unclear whether *Sall1* plays an active role in EpiSC reprogramming. In our work, we demonstrate that endogenous as well as exogenous *Sall1* can reprogram EpiSCs, and that *Sall1* synergizes with *Nanog* in reprogramming EpiSCs and MEFs. However, *Sall1* cannot replace *Oct4*, *Sox2*, or *Klf4* in MEF reprogramming, suggesting that it is unable to initiate the reprogramming machinery in more differentiated cells. One of its roles may be in facilitating epigenetic modification and nucleosome remodeling, e.g., through interaction with *Nanog* and the deacetylase complex (NurD) (Basta et al., 2017).

Unlike *Nanog*, the ability of *Sall1* to reprogram EpiSCs is insufficient to keep ESCs in the naive pluripotent state, only marginally delaying loss of pluripotency in differentiation experiments. However, it slowed expression of EpiSC markers *Fgf5* and *Otx2*, and preserved the ability to generate ESC-like colonies. *Sall1* inhibited *Otx2* expression in embryoid body differentiation of ESCs, and a formative pluripotent phase between naive and primed states was postulated when cells lost naive pluripotency markers and gained post-implantation markers such as *Otx2* and *Oct6* among others (Karantzali et al., 2011, Smith, 2017). Considering that, even after 21 days in differentiation medium, some *Sall1* overexpressing cells still formed ESC-like colonies in 2i/LIF, these cells may be stalled in a formative state.

We used RNA-seq to identify downstream targets of *Sall1* and *Nanog* in EpiSCs and found genes previously implicated in pluripotency or stem cell maintenance. *Esrrb*, a downstream target of *Nanog*, plays an important role in maintaining ESCs pluripotency and reprogramming by interacting with the core pluripotency network via *Sox2* (Adachi et al., 2013). *Tex10* was recently reported to be a pluripotency factor and partner of *Sox2*, capable of promoting MEF reprogramming (Ding et al., 2015), a role we further extended to EpiSC reprogramming. *Tet3* is a member of the ten-eleven translocation (Tet) protein family, which regulate DNA methylation. *Tet1* and *Tet2* have already been implicated in somatic reprogramming and *Tet2* has been reported to promote EpiSCs to a naive state (Bagci and Fisher, 2013, Fidalgo et al., 2016). Here, we show that *Tet3* can mediate EpiSC reprogramming as well.

The Kruppel-like factor family proteins *Klf2*, *4*, and *5* are also pluripotency factors and both *Klf2* and *Klf4* have been shown to facilitate reprogramming (Jeon et al., 2016). The potential of *Klf5* however is unclear as it has been reported to be incapable of reprogramming EpiSCs in a study by Hall et al. (2009), while Jeon et al. (2016) and recently Azami et al. (2018) both were able to derive iPSCs from EpiSCs by cDNA-mediated *Klf5* overexpression. We identified *Klf5* in our GoF screen and confirmed its ability to reprogram EpiSCs via CRISPRa transcriptional activation. These incongruent observations may reflect a Goldilocks effect similar to our observations with *Oct4*, highlighting the utility of different overexpression approaches to discover new pluripotency factors. LIF-dependent activation of Jak/Stat3 and its role in ESC self-renewal and reprogramming has been widely studied to date (Tang and Tian, 2013, Yu et al., 2017). Overexpression of *Klf5* via cDNA may compensate for the absence of LIF in maintaining ESC pluripotency and thereby be capable of reprogramming EpiSCs via LIF-independent pathways (Ema et al., 2008, Jeon et al., 2016). Besides *Klf5*, our data also indicate that *Sall1* positively and negatively regulates the Jak/Stat3 and TGF-β pathways, respectively (via *Gp130*, *Lif* receptor, and *Smad7*), together providing insights into the role of *Sall1* in EpiSC reprogramming.

*Fam189a2* was identified as a new target of *Nanog* in EpiSC reprogramming and our data showed that both *Nanog* and *Fam189a2* downregulate *Tgfbr1* and upregulate *Esrrb* expression. We postulate that the observed synergy between *Sall1* and *Nanog* as well as their downstream effectors *Klf5* and *Fam189a2* is partially due to the combined activation of Jak/Stat3, suppression of TGF-β signaling and upregulation of pluripotent genes such as *Esrrb*.

In conclusion, using a genome-scale CRISPR activation screen in the well-established EpiSCs reprogramming model, we identify known and previously unknown genes that can mediate cellular reprogramming in EpiSCs. We demonstrate that the transcription factor *Sall1* can effectively reprogram EpiSCs and MEFs, and provide new insights into the role of *Sall1* in promoting and maintaining pluripotency. Other reprogramming candidates such as transcription factors *Atf1* and *Bhlha15*, kinases *Idnk* and *Has1*, several olfactory receptor genes (*Olfr*), and others with less known functions such as *Umodl1* and *Prr3* deserve further in-depth investigation. Our studies demonstrate the strengths of CRISPR activation screens in the identification of factors that were previously not reported in molecular reprogramming and in illuminating biological pathways.

## Experimental Procedures

### GoF gRNA Library Design

In brief, the GoF library targeted the region of up to 250 bp upstream of the TSS of each protein-coding gene. Up to 4 guides of 19 bp length were selected per gene. Guide sequences with off-target sites exhibiting fewer than 3 mismatches over their 19 bp length were omitted from the design.

A selection algorithm was designed to spread high-quality guides across the target region. To this end, the region upstream of the TSS was divided into quarters of roughly equal length. Starting with the quarter closest to the TSS the algorithm looped over quarters picking the best guide, by quality score, in each if available, and adding it to the library until no more guide fitting the constraints could be found, or the target number of five guides per genes was reached. A constraint for the GC content of less than 55% was applied in the first loop and then relaxed to 70%.

### GoF Reprogramming Screen

The GoF sgRNA library was synthesized by Custom Array, and the oligo pools were cloned into the lentiviral sgRNA expression plasmid via Gibson assembly as described by Shalem et al. (2014), with minor modifications.

In brief, *Oct4*-GFP EpiSC cells stably expressing dCas9:SAM were first generated and were expanded to 100 × 10^6^ cells for lentiviral transduction of the GoF library. Library transduction was carried out at an MOI of 0.3. After 2 days, 10 × 10^6^ BFP^+ve^ *Oct4*-GFP EpiSCs were sorted by flow cytometry and plated in 2i/LIF in order to allow selection for reprogrammed cells. After 14–16 days in 2i/LIF, the individual reprogrammed colonies, verified by *Oct4*-GFP fluorescence, were picked and transferred to 96-well plates for colony expansion and genomic DNA extraction. PCR amplification on the genomic DNA, across the stably integrated sgRNA, was performed using primers described previously (Koike-Yusa et al., 2014) and NGS was used to identify the sgRNA sequences.

All experimental procedures are detailed out in the Supplemental Information.

## Author Contributions

J.Y. initiated and designed the project, performed GoF screening and EpiSC, MEF reprogramming with cDNA, analyzed the data, and wrote the manuscript. S.S.R. performed EpiSC reprogramming, ESC conversion, MEF reprogramming with gRNA, RNA-seq, analyzed the data, and wrote the manuscript. M.J.F. performed EpiSC reprogramming and RNA-seq data analysis, and wrote the manuscript. G.L. and X.Z. performed microinjection and analyzed chimera data. H.P. designed the GoF sgRNA library and performed RNA-seq data analysis. D.A.G. performed experiments to determine various dCas9-mediated overexpression levels. P.L. and A.B. financially supported the project and interpreted the data. E.M. initiated, designed, and supervised the project, performed GoF screening, validation, analyzed and interpreted data, and wrote the manuscript.

## Accession Numbers

The accession number for the data reported in this paper is ArrayExpress: E-MTAB-7692.

## Supplemental Information

Document S1. Supplemental Experimental Procedures, Figures S1–S4, and Tables S1–S4

Table S5. List of Gain-of-Function Guides against the Mouse Genome

Table S6. Results from the Gain-of-Function CRISPRa Screen

Table S7. List of Genes Validated for Reprogramming with Individual gRNAs

Table S8. Results from the RNA-Seq Experiment

Document S2. Article plus Supplemental Information
