# Supplementary material for: Genome-Scale CRISPRa Screen Identifies Novel Factors for Cellular Reprogramming
Source: Stem Cell Reports. 2019 Mar 21;12(4):757–71. doi: 10.1016/j.stemcr.2019.02.010 (PMC6450436; doi:10.1016/j.stemcr.2019.02.010)
Supplement: Document S1. Supplemental Experimental Procedures, Figures S1–S4, and Tables S1–S4 [file mmc1.pdf]

Stem Cell Reports, Volume *12*

Supplemental Information

Genome-Scale CRISPRa Screen Identifies Novel Factors for Cellular Reprogramming

Jian Yang, Sandeep S. Rajan, Mathias J. Friedrich, Guocheng Lan, Xiangang Zou, Hannes Ponstingl, Dimitrios A. Garyfallos, Pentao Liu, Allan Bradley, and Emmanouil Metzakopian

Genome-scale CRISPRa screen identifies novel factors for cellular reprogramming

Jian Yang[‡⊥+], Sandeep S Rajan[‡†+], Mathias J Friedrich[‡+], Guocheng Lan[¶], Xiangang Zou[¶], Hannes Ponstingl[‡], Dimitrios A Garyfallos[‡], Pentao Liu[‡¢], Allan Bradley[‡], and Emmanouil Metzakopian[‡†*]

[‡]Wellcome Trust Sanger Institute, Genome Campus, Hinxton, Cambridgeshire, CB10 1SA, UK

[†]UK Dementia Research Institute, Department of Clinical Neuroscience, University of Cambridge, CB2 0AH, UK

[¶]Cancer Research UK, Cambridge Institute, Li Ka Shing Centre, University of Cambridge, Cambridge, CB2 0RE, UK

[¢]School of Biomedical Sciences, Li Ka Shing Faculty of Medicine, Stem Cell and Regenerative Medicine Consortium, University of Hong Kong, Hong Kong, China

[⊥]*Current address:* Key Laboratory of Arrhythmias, Ministry of Education, Shanghai East Hospital, Tongji University School of Medicine, Shanghai 200120, China

[+]These authors contributed equally to the work.

*Correspondence should be addressed to:

Emmanouil Metzakopian

UK Dementia Research Institute
Cambridge Biomedical Campus
University of Cambridge
Cambridge, CB2 0AH, U.K

Email: em698@medschl.cam.ac.uk

Supplementary Document

Experimental Procedures

Ethics Statement

All animal experimental procedures were carried out in accordance with Home Office UK regulations and the Animals (Scientific Procedures) Act 1986 (licence No. 70/8387 and 80/2552). All experimental protocols were approved by the Animal Welfare and Ethical Review Body (AWERB) of Wellcome Genome Campus and the University of Cambridge CRUK Cambridge Institute. At the end of the study, mice were euthanized in accordance with stated Home Office UK regulations.

Constructs

Guide RNAs (sgRNAs) were expressed under the U6 promoter in expression constructs (pKLV-PB-U6-gRNA-PGK-Puro-T2A-TagBFP, Figure S1) harbouring *PiggyBac inverted terminal repeats to enable transposase-mediated genomic integration* (*PB* transposon) and HIV-1 long terminal repeats to allow lentiviral genomic integration (Figure S1). The sgRNA scaffold used in conjunction with dCas9:SAM was adapted to contain two MS2-binding loops as required by the SAM CRISPRa system (Konermann et al. 2015). The constructs also included a puromycin antibiotic resistance and a TagBFP marker. The four dCas9 variants were cloned into PB transposons and included the mCherry fluorescent marker and Blasticidine antibiotic resistance. The dCas9 cDNAs in these constructs were driven by the Ef1-α promoter and multiple consecutive cDNAs were linked by the T2A self-separating peptide sequence.

cDNAs of *Oct4*, *c-Myc*, *Klf4*, *Sox2*, *Sall1* and *Nanog* were cloned into *PiggyBac* transposons under the control of the CAG promoter (PBCAG) or the Tet response element (PBTRE). Combinations of cDNAs were linked by the T2A self-separating peptide sequence. PBEF1α-TET3G encoding the Tet-On-3G transactivator protein was co-transfected with PBTRE-cDNA to enable Doxycycline induction of the PBTRE constructs.

When stable integration by transposition of the transgene was required, a plasmid encoding *PiggyBac* transposase (*HyPBase* (Yusa et al. 2011)) was co-transfected. Schematic representations of all the constructs used in the study are shown in Figure S1.

Cell Culture

Oct4-GFP and *Nanog*-GFP Epiblast stem cells (EpiSCs) were generated as described previously (Yang et al. 2010). Briefly, EpiSC were derived from post-implanted pregnant transgenic mice at E5.75. Cells from embryos were cultured on human fibronectin (Millipore) coated plates in complete EpiSC media based on N2B27 which comprised 50% Neurobasal media, 50% DMEM-F12 media, 0.1mM β-ME, penicillin (100 U/ml), streptomycin (100 μg/ml), L-glutamine (2 mM), 1X N2 and 1X B27 supplement (Invitrogen) and was supplemented with 20 ng/ml Activin-A (R & D Systems) and 12 ng/ml FGF2 (Peprotech). When confluent, the media was aspirated and the EpiSC were dissociated with Accutase (Millipore) for 3 min. Dissociated cells were spun down at 300g for 5 min and plated either at 1:6 or at 1:8 split ratio on human fibronectin coated plates in complete EpiSC media for maintenance.

For screening and reprogramming, EpiSCs were cultured in 2i/LIF medium comprising N2B27 media (as above) supplemented with 100 U/ml leukemia inhibitory factor (LIF, Millipore), 1 μM PD0325901 (Tocris) and 3 μM CHIR99021 (Tocris).

Rex1-GFP embryonic stem cells (ESCs) cells were generated as described previously (Wang et al. 2011). ESCs were cultured on 0.1% gelatin coated plates. ES cells were regularly maintained in ESC medium (M15) comprising knock-out DMEM containing 15% FBS, 0.1mM β-ME, penicillin (100 U/ml), streptomycin (100 μg/ml), L-glutamine (2 mM), and 100 U/ml LIF. Confluent ES cells were dissociated with 0.025% Trypsin-EDTA for 5 min. Detached cells were collected and spun down at 300g for 5 min and were plated at a split ratio of either 1:8 or at 1:10 on gelatin coated plates.

Mouse embryonic fibroblasts (MEFs) were cultured in M10 medium comprising knock-out DMEM containing 10% FBS, 0.1mM β-ME, penicillin (100 U/ml), streptomycin (100 μg/ml) and L-glutamine (2 mM). For reprogramming, after transfection, cells were cultured in ESC medium until the end of experiment or until the colonies were picked for iPSC line derivation.

GoF gRNA library design

The Gain of Function (GoF) library targeted the region of up to 250 base pairs upstream of the transcription start site (TSS) of each protein-coding gene. Up to 4 guides of 19 bp length were selected per gene. Protein coding genes and TSSs were obtained from the mouse reference assembly GRCm38 in the ENSEMBL database (version 78, http://www.ensembl.org) and TSSs were checked against CAGE data in the FANTOM data base (April 2015, http://fantom.gsc.riken.jp/).

Potential guide sequences were identified on the reference assembly as the 19 bp sequence at the 5' end of each PAM motif (NGG), i.e. 2 to 20 bp upstream of each

guanosine di-nucleotide in the reference. Guide sequences consisted of no more than 13 guanosine or cytosine bases (GC content < 70%). Guides in the 250 bp region on both DNA strands upstream of the TSS were then compared to all other potential guide sequences across the genome. Guide sequences with off-target sites exhibiting fewer than 3 mismatches over their 19 bp length were omitted from the design.

The remaining guides were sorted by a simple ad-hoc quality score intended to reflect a likely increased tolerance of mismatches distal of the PAM motif. For each potential off-target site a score was calculated that linearly increased with the number of mismatches and decreased with their distance to the PAM motif.

A selection algorithm was designed to spread high quality guides across the target region. To this end, the region upstream of the TSS was divided in quarters of roughly equal length. Starting with the quarter closest to the TSS the algorithm looped over quarters picking the best guide, by quality score, in each if available and adding it to the library until no more guide fitting the constraints could be found, or the target number of 5 guides per genes was reached. A constraint for the GC content of less than 55% was applied in the first loop and then relaxed to 70%.

GoF reprogramming screen

The GoF sgRNA library was synthesized by Custom Array, and the oligo pools were cloned into the lentiviral sgRNA expression plasmid via Gibson assembly as described by Shalem *et al*.(Shalem et al. 2014), with minor modifications.

Oct4-GFP EpiSC cells were transfected with 200 ng of plasmid encoding *PiggyBac* transposase together with 1 µg dCas9:SAM to facilitate stable integration. Transfections were performed with Lipofectamine LTX (Invitrogen), according to manufacturer's instructions. Transfected cells were then selected by 10 µg/ml

Blasticidine (Gibco) for 10 days. Post-selection, dCas9:SAM expressing *Oct4*-GFP EpiSC were expanded to 100 x 10^6 cells for lentiviral transduction.

Library transduction was carried out at a MOI (multiplicity of infection) of 0.3. After two days, 10 x 10^6 BFP^{+ve} *Oct4*-GFP EpiSCs were sorted by flow cytometry. The sorted cells were seeded on fibronectin coated plates and allowed to recover in complete EpiSC medium for 24 h. The medium was then changed to 2i/LIF in order to allow selection for reprogrammed cells. After 14-16 days in 2i/LIF, the individual reprogrammed colonies, verified by *Oct4*-GFP fluorescence, were picked and transferred to 96 well plates. Colonies were passaged twice in 2i/LIF before sequencing. Genomic DNA was extracted from each colony and PCR amplification across the stably integrated sgRNA was performed using primers described previously (Koike-Yusa et al. 2014). PCR amplicon libraries were pooled and Next Generation Sequencing was used to identify the distribution of sgRNA sequences.

CRISPRa transfections

Oct4-GFP EpiSC and *Nanog*-GFP EpiSC cells were grown to 70% confluence on fibronectin coated 6-well plates. The cells were dissociated with Accutase and re-suspended in EpiSC media for reverse transfections (approx. 1 X 10^6 cells in 250 µl per transfection).

Cells were transfected with 500 ng *PiggyBac* transposase together with 500 ng dCas9:SAM and 500 ng of sgRNA expression construct for one or more sgRNAs. Transfections were performed, in triplicate, using Lipofectamine LTX (Invitrogen), according to manufacturer's instructions. Cells were cultured in EpiSC medium for 24 h. Stably-transfected cell lines were generated by selection with Blasticidine

(10 µg/ml) for at least 10 days post-transfection. Integration of constructs was confirmed by PCR genotyping (Table S1).

cDNA transfections

Oct4-GFP EpiSCs, *Nanog*-GFP EpiSCs and *Rex1*-GFP ESCs were transfected, in triplicate, with 1 µg PBCAG expressing either *Sall1*, *Nanog* or a combination of both vectors via Lipofectamine LTX. Cells were co-transfected with plasmids encoding *PiggyBac* transposase (500 ng) and mCherry Blasticidine (100 ng) selection markers to enable selection of transfected cells. Presence of PBCAG in the selected cells was confirmed with PCR (Table S1).

EpiSC Reprogramming

Stable lines of *Oct4*-GFP and *Nanog*-GFP EpiSC generated either from CRISPRa or cDNA transfections were plated in triplicate on fibronectin coated 6-well plates in EpiSC medium. Medium was changed to 2i/LIF when cells reached 80% confluence and thereafter replaced every 2 days to select for reprogramming for up to 20 and 24 days for *Oct4*-GFP transfected EpiSCs and *Nanog*-GFP transfected EpiSCs, respectively. GFP^{+ve} ESC-like colonies were counted and transferred onto gelatin coated plates for expansion. Expanded colonies were then phenotyped for gene expression by RT-qPCR (Table S3).

To derive iPSCs for blastocyst injection, *Oct4*-GFP EpiSC were transfected with 1 µg PBTRESall1, 1 µg PBTET3G and 2 µg transposase using Lipofectamine LTX. Transgene expression was induced by supplementing the medium with 0.5 µg/ml Doxycycline after switching the cells to 2i/LIF.

Flow cytometry analysis of EpiSC reprogramming

Stably transfected CRISPRa *Oct4*-GFP and CRISPRa *Nanog*-GFP EpiSC were plated in triplicates on fibronectin coated 24-well plates at a density of 50,000 cells per well, in EpiSC media. Upon reaching confluence, the media was changed to 2i/LIF to select for reprogramming. Cells were harvested at regular time intervals and were analysed for GFP fluorescence in a flow cytometer (CytoFLEX, Beckman Coulter Life Sciences, Indianapolis US).

MEF Reprogramming

For sgRNA mediated reprogramming of MEFs, 1×10^6 *Oct4*-GFP MEFs were electroporated with 0.5 µg PBCAG4F, 1 µg gRNA Sall1 / 1 µg gRNA Nanog / 0.5 µg gRNA Sall1+0.5 µg gRNA Nanog, 1 µg dCas9:SAM and 0.5 µg *PiggyBac* transposase using the Amaxa Nucleofector (Amaxa, Lonza). The transfected cells were plated onto gelatin-coated 10 cm dishes in M15. All transfections were performed in triplicates. After 24 h, the medium was replaced and was renewed every two days until day 18 when *Oct4*-GFP^{+ve} and AP^{+ve} cells were counted.

For inducible cDNA mediated reprogramming, 1×10^6 *Oct4*-GFP MEFs were electroporated with 1 µg PBTRE4F, 0.5 µg PBTRESall1, 2 µg PBEF-1αTet3G and 2 µg *PiggyBac* transposase using the Amaxa Nucleofector (Amaxa, Lonza). The transfected cells were plated in gelatin-coated 10 cm dishes in ESC medium. All transfections were performed in triplicates. After 24 h, the medium was replaced and three different concentrations of Doxycycline (0.1, 0.5 and 1.0 µg/ml) were tested for induction of cDNA expression. At day 12, Doxycycline was withdrawn and the cells were cultured for 6 more days, after which *Oct4*-GFP^{+ve} and alkaline phosphatase stained colonies were counted and single colonies picked for RT-qPCR.

To produce iPSCs for *in vitro* and *in vivo* assays, C57B/6J MEFs were transfected with the same amount of PBTRE4F, PBTRESall1, PBEF1α-TET3G and *PiggyBac* transposase as described above and induced with 0.5 μg/ml Doxycycline in ESC media.

All the combinations of cDNA transfections for MEF reprogramming are listed in Table S2.

Western Blotting

Oct4-GFP EpiSCs transfected with CRISPRa *Oct4* and cDNA *Oct4* (both CAG and TRE) together with experimental controls and untransfected cells (EpiSCs and ESCs) were collected for Western blotting after 3 days 2i/LIF (Dox induction for 3 days). Protein amounts were determined using a Bradford assay and 30 μg of lysates were subjected to electrophoresis on 4–15% Mini-PROTEAN® TGX™ Precast Protein Gels (Biorad). Proteins were transferred onto PVDF membranes (Millipore) overnight at 30 V for 8 hours at 4°C. Transferred proteins were then immunoblotted for Oct4 (c-10, Santa Cruz, #sc-5279, dilution 1:800) and Gapdh (Sigma, #G8795, dilution 1:4000). All antibodies used are listed in Table S4.

Conversion of ESCs to EpiSCs

Stable lines of cDNA transfected *Rex1*-GFP ESCs were cultured in 2i/LIF medium for 3 days prior to the conversion. Upon reaching 50% confluence, cells were dissociated and seeded on gelatin-coated plates in EpiSC medium for conversion for at least 3 passages. Cells were seeded on fibronectin-coated plates after the 2nd passage to promote differentiation. In addition, 600 cells were plated back on 0.1% gelatin coated plates in 2i/LIF medium for about 7 days to promote formation of iPS colonies. The colonies were assessed by AP staining. Lastly, at every passage, cells were assessed

for *Rex1*-GFP fluorescence using flow cytometry and total RNA was extracted for RT-qPCR. All ESC to EpiSC conversion experiments were carried out in triplicates.

Quantitative RT-PCR (RT-qPCR)

Total RNA was extracted using the RNeasy Mini Kit (Qiagen) according to manufacturer's instructions. First strand cDNA was synthesized using qScript cDNA Supermix (Quantabio) according to manufacturer's protocol. All qPCR studies were performed using Taqman Gene Expression Assays either in the 7900HT Fast Real-Time PCR system (Applied Biosystems) or the StepOnePlus™ Real-Time PCR System (Applied Biosystems). Samples were run in triplicate, from 3 independent experiments, for both gene of interest and house-keeping genes. Expression levels were normalized to *Gapdh*. TaqMan probes used are listed in Table S3.

RNA-sequencing

Oct4-GFP EpiSCs were transfected via Lipofectamine LTX with 1 µg PBCAGSall1, PBCAGNanog, PBCAGSall1+PBCAGNanog or empty vector (PBCAG:Empty). Cells were co-transfected with CAGmCherry (100 ng) to enable fluorescent marker selection. All transfections were performed in triplicates. 24 h later, cells were FACS sorted for GFP^{+ve}mCherry^{+ve} and their RNA was extracted using the Rneasy Mini Kit (Qiagen) according to manufacturer's instructions. Illumina Truseq NGS libraries were prepared and sequenced on a Illumina HiSeq instrument.

Sequencing results were analysed by aligning reads to the mouse genome build GRCm38.p5 using the STAR aligner (Ver. 2.5.3a) and read counts were prepared with the TOPHAT package. Differential expression of genes was analysed with the DESeq2 package for the R statistical computing framework. We used a cut-off padj value of <0.001 to determine genes that were differentially regulated between

experimental and control samples. All data from RNA-seq is available from ArrayExpress (https://www.ebi.ac.uk/arrayexpress/) using accession number: E-MTAB-7692.

GO Term analysis

We used the GoToolBox platform (http://genome.crg.es/GOToolBox/) to perform enrichment analysis on the genes identified in the CRISPRa screen and via RNAseq as described previously (Castro et al. 2011). In brief, a Data-Set was created using the Mouse Genome Informatics version (MGI) and then used for pathway enrichment analysis. Fold changes for enriched gene sets were computed by dividing the frequency in our set by that of the reference. Representative pathways with a fold change of 2 or more and with a p-value of 0.05 or lower were graphically represented in the results.

Immunofluorescence

C57BL/6J MEF reprogrammed iPSCs were plated at 2 x 10^3 onto 24 well plates in 2i/LIF. After 24 hours, cells were washed with PBS and fixed in 4% paraformaldehyde (PFA), blocked and permeabilised with 1% bovine serum albumin (BSA) and 3% serum in PBS with 0.1%Triton X100. Samples were incubated with mouse anti-SSEA-1 (BD Biosciences) or rabbit anti-*Nanog* (Abcam) antibodies at 4℃ overnight, then rinsed and incubated with Alexa488-conjugated goat anti-Mouse IgM and Alexa594-conjugated goat anti-Rabbit IgG (Invitrogen), and counterstained with DAPI.

To examine the X chromosome status in female iPSCs, cells were plated at 5 x 10^3 on gelatin coated slides in 2i/LIF or in M10 medium for 5 days, then cells were fixed in 4% PFA, and immunofluorescence was performed as mentioned above. Slides were incubated with rabbit anti-H3K27me3 (Millipore) and mouse anti-*Oct4* antibodies

(Santa Cruz), then Alexa594-conjugated goat anti-Rabbit IgG and Alexa488 goat anti-Mouse IgG and counterstained with DAPI. All antibodies used are listed in Table S4.

In Vitro differentiation

C57BL/6J MEF reprogrammed iPSCs were plated at 5 x 10^5 in a petri-dish in M10 medium for 4 days, then dissociated with 0.05% Trypsin/EDTA and plated at 1 x 10^5 in M10 on gelatinized plates for another 4 days. The cells were then fixed in 4% PFA and examined for mesoderm and endoderm markers using immunofluorescent staining with antibodies against smooth muscle antigen (SMA) (R&D Systems) and alpha fetoprotein (AFP) (R&D Systems). For neuronal differentiation, cells were plated at 1.5 x 10^5 in N2B27 medium on gelatinized plates. The medium was changed every other day and at day 8, the cells were fixed and stained with anti-beta tubulin III (Tuj1) antibody (R&D Systems).

Chimeras

Chimeras were produced by a standard microinjection protocol. Chimerism was estimated based on coat colour since iPSCs derived from EpiSCs and MEFs are of MF1 and C57BL/6J genetic background (agouti and black furs) whereas the host blastocysts were from albino C57BL/6.

Statistical analyses

All statistical analyses were performed in GraphPad Prism (Version 6.01). Data are presented as means ± standard deviations. Statistical significance was determined either using a Student's unpaired *t*-test with two-tailed distribution or Two-way ANOVA. Students' t-test was used for comparison across two groups while Two-way ANOVA with multiple comparisons were performed on samples undergoing a time course experiment. *p*-values less than 0.05 were considered significant.

Supplementary Figure Legends

Supplementary Figure S1 | Related to main Figure 1: Plasmids used in the study

LTR: long terminal repeat from HIV-1. **PB:** *PiggyBac* inverted terminal repeats to enable stable integration by *PBase* mediated transposition. **U6:** U6 promoter for sgRNA transcription. **sgRNA:** sgRNA scaffold. **sgRNA-MS2-MS2:** sgRNA scaffold with extended stem loops for dCas9:SAM. **PGK:** PGK promoter. **PuroR:** puromycin N-acetyltransferase. **T2A:** 2A peptide from *Thosea asigna* virus capsid protein. **BFP:** blue fluorescent protein. **Poly(A):** polyadenylation signal. **EF1-α:** human elongation factor 1 alpha promoter. **VP160-dCas9:** 10 tandem repeats of transcriptional activation domain of herpes simplex virus protein VP16 fused to dCas9. **mCherry:** mCherry fluorescent protein. **P2A:** 2A peptide from *porcine teschovirus-1* polyprotein. **BlastR:** *Aspergillus terreus* blasticidin S deaminase. **GCN4:** GCN4 single chain antibody. **VP64:** 4 tandem repeats of transcriptional activation domain of herpes simplex virus protein VP16. **dCas9-SunTag:** GCN4 peptide fused to dCas9. **dCas9-VPR:** VP64, P65 and RTA (transcriptional activation domain from the human herpesvirus 4) fused to dCas9. **MS2:** bacteriophage MS2 coat protein. **P65:** C-terminal portion of the p65 subunit of mouse NF-κB. **HSF1:** C-terminal activation domain from the human heat shock transcription factor. **E2A:** 2A peptide from equine rhinitis A virus polyprotein. **bpA:** bovine growth hormone polyadenylation signal. **CAG:** CMV enhancer, chicken β-actin and rabbit β-globin promoter. **TRE:** tet response element. **TET3G:** Tet-On-3G transactivator protein.

Supplementary Figure S2 | Related to main Figures 1 and 2: Supplementary results for GoF CRISPRa screen and gene dosage of *Oct4*

(a) Flow cytometry plots showing dynamics of GFP expression in *Oct4*-GFP reporter EpiSCs during reprogramming: *Oct4*-GFP EpiSCs transfected with dCas9:SAM +

sgRNA against gene *Nanog*, dCas9:SAM only or untransfected. Over a time course of 14 days in selective 2i/LIF medium, the GFP positive cell population rapidly and completely disappears in the untransfected or dCas9:SAM only groups. However, cells reprogrammed by CRISPRa mediated overexpression of *Nanog* recover their *Oct4*-GFP levels by day 14. **(b)** Flow cytometry plots showing the percentage of BFP^{+ve} cells obtained upon transducing *Oct4*-GFP EpiSCs with lentiviral sgRNA library at an MOI of 0.0 (untransduced) and an MOI of 0.3. **(c)** RT-qPCR on *Oct4*-GFP EpiSCs transfected with CRISPRa and cDNA Oct4 showing levels of total *Oct4* in cells after 3 days in 2i/LIF. Levels of *Oct4* are normalized to *Gapdh* (error bars represent mean of experimental triplicates ± s.d.). **(d)** Western blot on *Oct4*-GFP EpiSCs transfected with CRISPRa and cDNA *Oct4* showing levels of total Oct4 in cells after 3 days in 2i/LIF (top panel). Western blot for Oct4 in gRNA and CAG cDNA transfected EpiSCs with a comparison to ESCs (bottom panel) *Gapdh* was used as loading control in both cases. **(e)** Variable gene dosage with tiled guides: *Oct4*-GFP EpiSCs were transfected with dCas9:SAM and all tiled sgRNAs for *Sall1* and *Nanog* available in our library. Numbers on top of bars indicate distance to the transcription start site (TSS). RT-qPCR expression levels of *Sall1* and *Nanog*, on tiled sgRNA transfected cells, normalized to *Gapdh* and relative to dCas9:SAM (error bars represent mean of experimental triplicates ± s.d.). **(f)** *Umodl1* regulates reprogramming pathways: RT-qPCR expression levels of key regulators in JAK/STAT3 and TGFβ signalling, on flow-sorted for sgRNA expression either 24 h after transfection (EpiSC media) or 24 h after changing to 2i/LIF media (48 h after transfection), normalized to *Gapdh* and relative to dCas9:SAM (error bars represent mean of 3 independent experiments ± s.d.), ** $p<0.01$, *** $p<0.001$. **(g)** RT-qPCR shows that *Sall4* is not overexpressed when

Oct4-GFP+ve EpiSC are transfected with *Sall1* CRISPRa relative to dCas9:SAM (error bars represent mean of 3 independent experiments ± s.d.).

Supplementary Figure S3 | Related to main Figure 2: Reprogramming in *Nanog*-GFP reporter EpiSCs and supplementary results for ESC to EpiSC conversion

(a,b) Gene induction of *Sall1*, *Nanog* and *Oct4* in *Oct4*-GFP EpiSC **(a)** and *Nanog*-GFP EpiSCs **(b)**, respectively, with CRISPRa (single sgRNA per target) as well as cDNA mediated overexpression. Expression levels were measured by RT-qPCR 72h after transfection and expressed in relation to dCas9:SAM-only or empty vector (PBCAG:Empty) (error bars represent mean of 3 independent experiments ± s.d). **(c)** Reprogramming efficiencies of *Sall1*, *Nanog* and *Oct4*. *Nanog*-GFP EpiSCs were stably transfected with CRISPRa or cDNA and cultured in 2i/LIF for 16 days without selection. *Nanog*-GFP^{+ve} colonies were counted and are represented as mean of 3 independent experiments ± s.d ** p<0.01. **(d)** RT-qPCR expression profiles of pluripotency markers and EpiSC markers in iPSC colonies normalized to *Gapdh* and relative to EpiSCs (error bars represent mean of 3 independent experiments ± s.d). Cells were transfected with cDNA as before. **(e)** Chimeric mouse produced with cDNA *Sall1* – induced PSCs injected into C57B/6 blastocyst. **(f)** Identical endpoints of *Oct4*-GFP and *Nanog*-GFP reporter EpiSCs in reprogramming: *Oct4*-GFP EpiSCs (top panel) and *Nanog*-GFP EpiSCs (bottom panel) transfected with either dCas9:SAM alone or in combination with sgRNAs against *Sall1*, *Nanog* and *Oct4* and selected in 2i/LIF medium in a time course of 14 and 22 days. As before, *Oct4*-GFP EpiSCs lose initial GFP expression rapidly and recover it upon successful reprogramming via CRISPRa mediated gene induction of *Sall1*, *Nanog* or *Oct4*, but not in cells transfected with dCas9:SAM only. *Nanog*-GFP reporter EpiSCs – showing no baseline GFP expression – nevertheless upregulate GFP with similar but slightly slower dynamics in

sgRNA transfected groups only. On day 14 both reporter lines show comparable GFP positive cell populations and a clear synergistic effect when co-transfecting sgRNAs against *Sall1* and *Nanog*. Values are mean of 3 independent experiments ± s.d ** $p<0.01$, *** $p<0.001$, **** $p<0.0001$ **(g-i)** *Rex1*-GFP^{+ve} ES cells were stably transfected with *Sall1* or *Nanog* cDNA, or empty vector and cultured in EpiSC medium. Change of expression levels of pluripotency marker *Rex1* and differentiation markers *Fgf5* and *Otx2* measured by RT-qPCR normalized to *Gapdh* expression (error bars represent mean of 3 independent experiments ± s.d).

Supplementary Figure S4 | Related to main Figures 3 and 4: *Sall1* cannot replace *Oct4* in MEF reprogramming and supplementary results for RNA-seq

(a) (4F+CRISPRa) MEFs were stably transfected with CAG4F and gRNAs against *Sall1*/*Nanog*/*Sall1*+*Nanog* and reprogrammed in ESC medium. AP^{+ve} colonies were counted after 18 days of reprogramming. Gene induction of *Sall1* produced more iPSC colonies compared to CAG4F alone and gene induction of both *Sall1* and *Nanog* produced significantly higher number of colonies compared to activation of either *Sall1* or *Nanog* alone (error bars represent mean of 3 independent experiments ± s.d., ** $p<0.01$). (4F+cDNA) MEFs stably transfected with TRE4F, TRENanog and TRESall1 (all co-transfected with PBEF-1αTet3G) and induced with 0.5 µg/ml Doxycycline for 12 days. Overexpression of *Sall1* produced more iPSC colonies compared to CAG4F alone and overexpression of both *Sall1* and *Nanog* produced significantly higher number of colonies compared to activation of either *Sall1* or *Nanog* alone. AP^{+ve} colonies were counted on day 18 (error bars represent mean of 3 independent experiments ± s.d., *** $p<0.001$). **(b)** Morphology of *Oct4*-GFP^{+ve} colonies at day 18 in ESC media. The reprogrammed colonies from *Oct4*-GFP^{+ve} MEFs are morphologically similar to ESC colonies with *Oct4*-GFP^{+ve} fluorescence. No

colonies were observed in untransfected or dCas9:SAM only transfected MEFs. **(c)** MEF were stably transfected with CAG4F and TRESall1/PBEF-1αTet3G and reprogrammed in ESC medium. 24h after transfection, expression of Sall1 was induced with different concentration of Doxycycline for 12 days. After 18 days, both *Oct4*-GFP^{+ve} and AP^{+ve} colonies were counted. Overexpression of *Sall1* produced more iPSC colonies (error bars represent mean of 3 independent experiments ± s.d., ** p<0.01, *** p<0.001, $^{\Delta}$ p<0.05, $^{\Delta\Delta}$ p<0.01 vs. 4F). **(d)** MEFs stably transfected with CAG4F and TRESall1/PBEF-1αTet3G and reprogrammed in ESC medium. 0.5 µg/ml Doxycycline was added on the indicated days for the duration of reprogramming. *Oct4*-GFP^{+ve} and AP^{+ve} colonies were counted on D18 (error bars represent mean of 3 independent experiments ± s.d., ** p<0.01, $^{\Delta\Delta\Delta}$ p<0.001 vs. 4F). **(e-g)** MEFs were stably transfected with combinations of *Oct4*, *C-myc*, *Klf4*, *Sox2* (OCKS) and *Sall1* cDNA, whereby *Sall1* replaced either **(e)** *Klf4*, **(f)** *Sox2* or **(g)** *Oct4*. Cells were reprogrammed in ESC medium for 18 days and AP^{+ve} colonies were counted (error bars represent mean of 3 independent experiments ± s.d). **(h)** Venn diagram showing number and percentage of genes being differentially expressed in *Oct4*-GFP EpiSC transfected with cDNA for *Sall1/Nanog/Sall1+Nanog* for RNA-seq. Differentially expressed genes were identified with a cut-off padj value < 0.001. **(i)** CAG cDNA mediated overexpression of *Sall1*, *Nanog* in *Oct4*-GFP EpiSC transfected with either *Sall1*/*Nanog*/*Sall1+Nanog* for RNA-seq. Expression levels were measured by RT-qPCR 48h after transfection and expressed in relation to empty vector (error bars represent mean of 3 independent experiments ± s.d).

SUPPLEMENTARY FIGURE S1

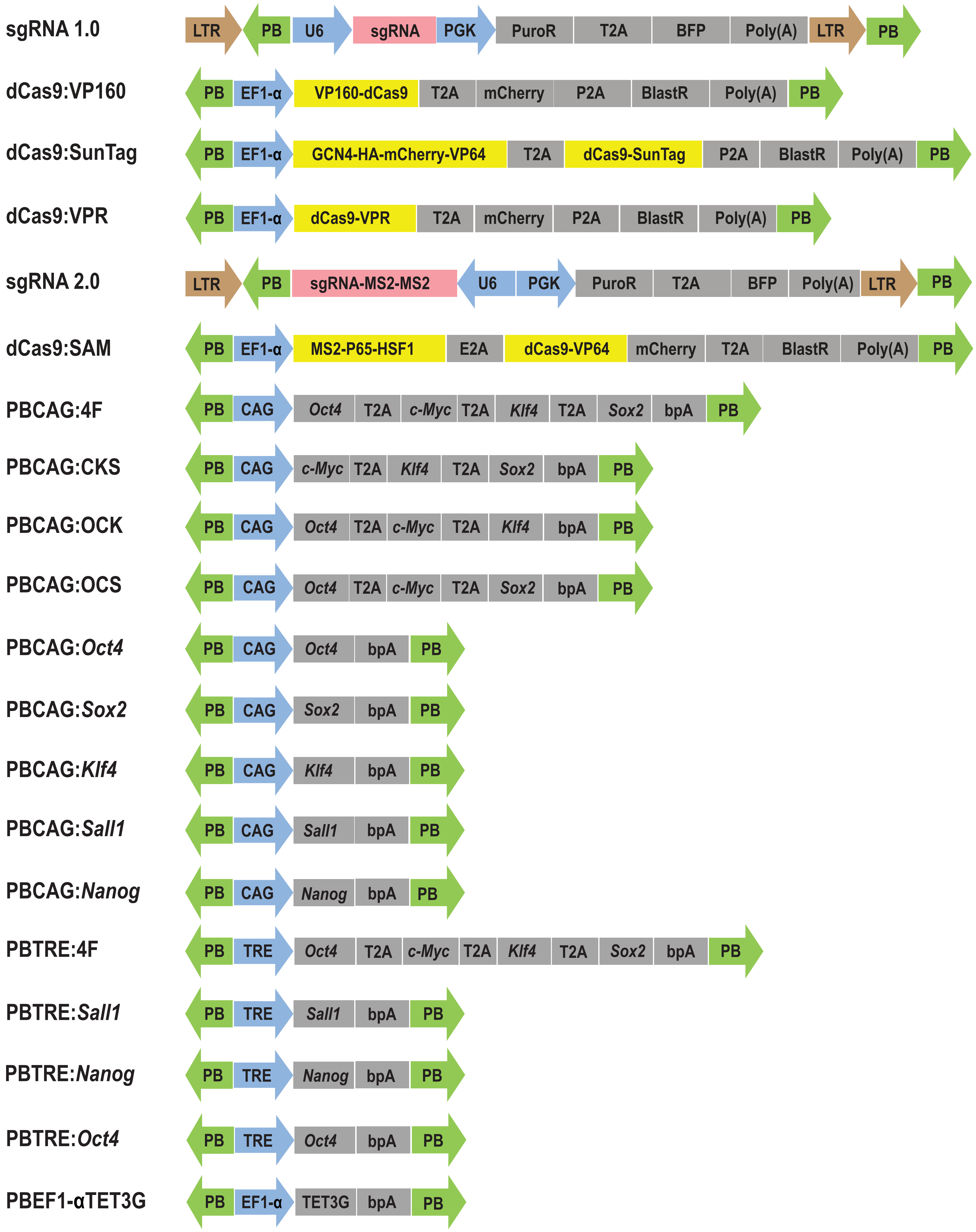

SUPPLEMENTARY FIGURE S2

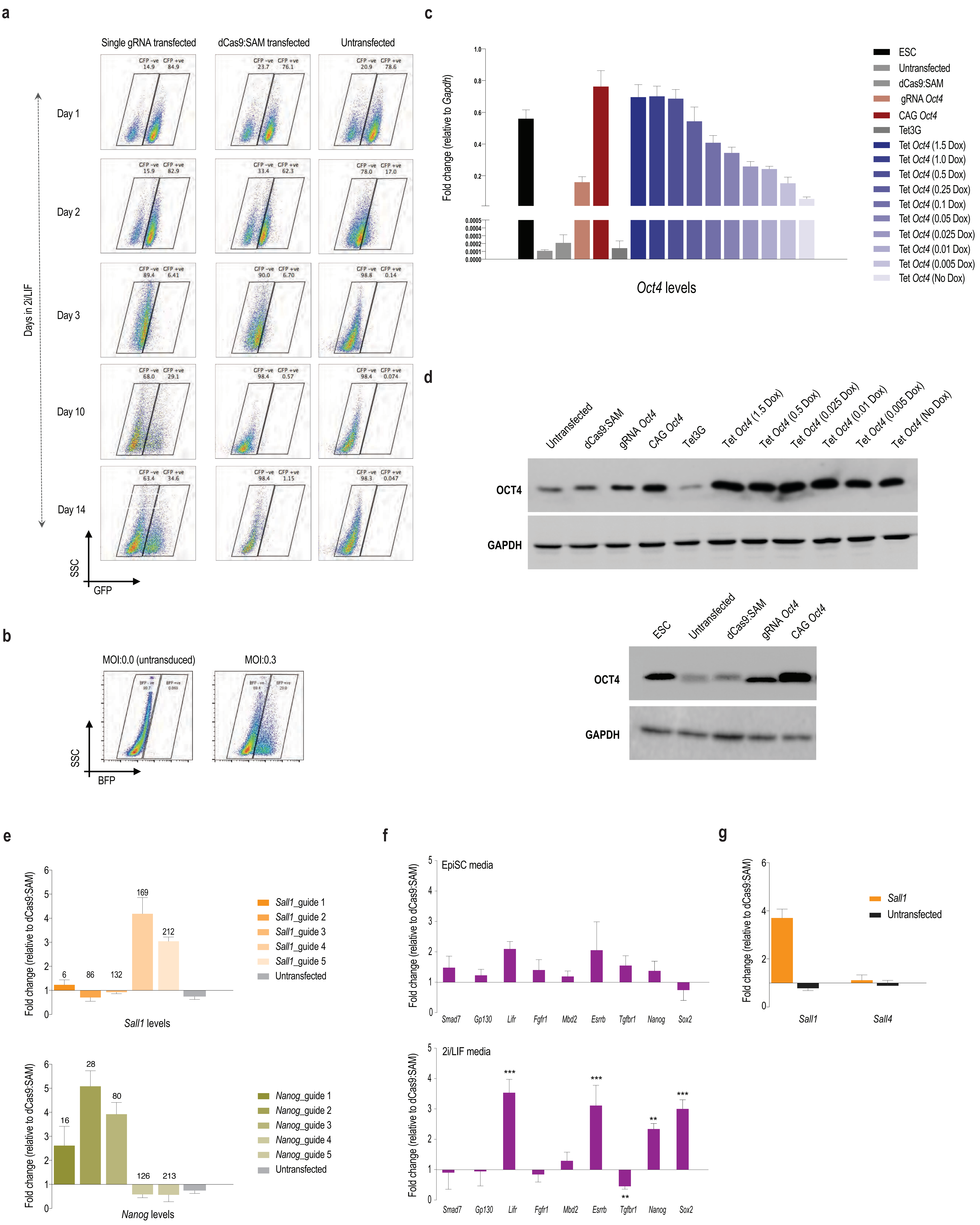

SUPPLEMENTARY FIGURE S3

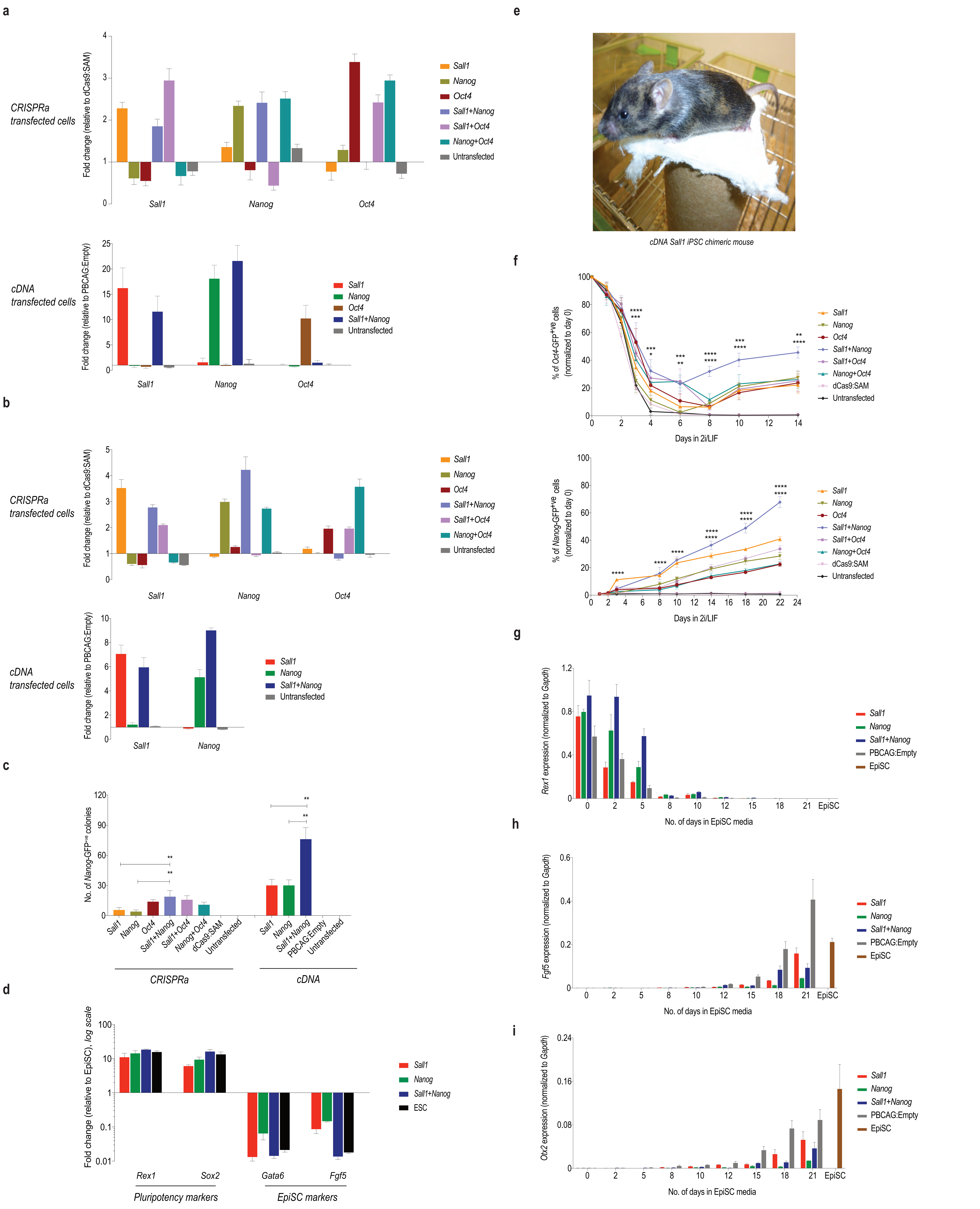

SUPPLEMENTARY FIGURE S4

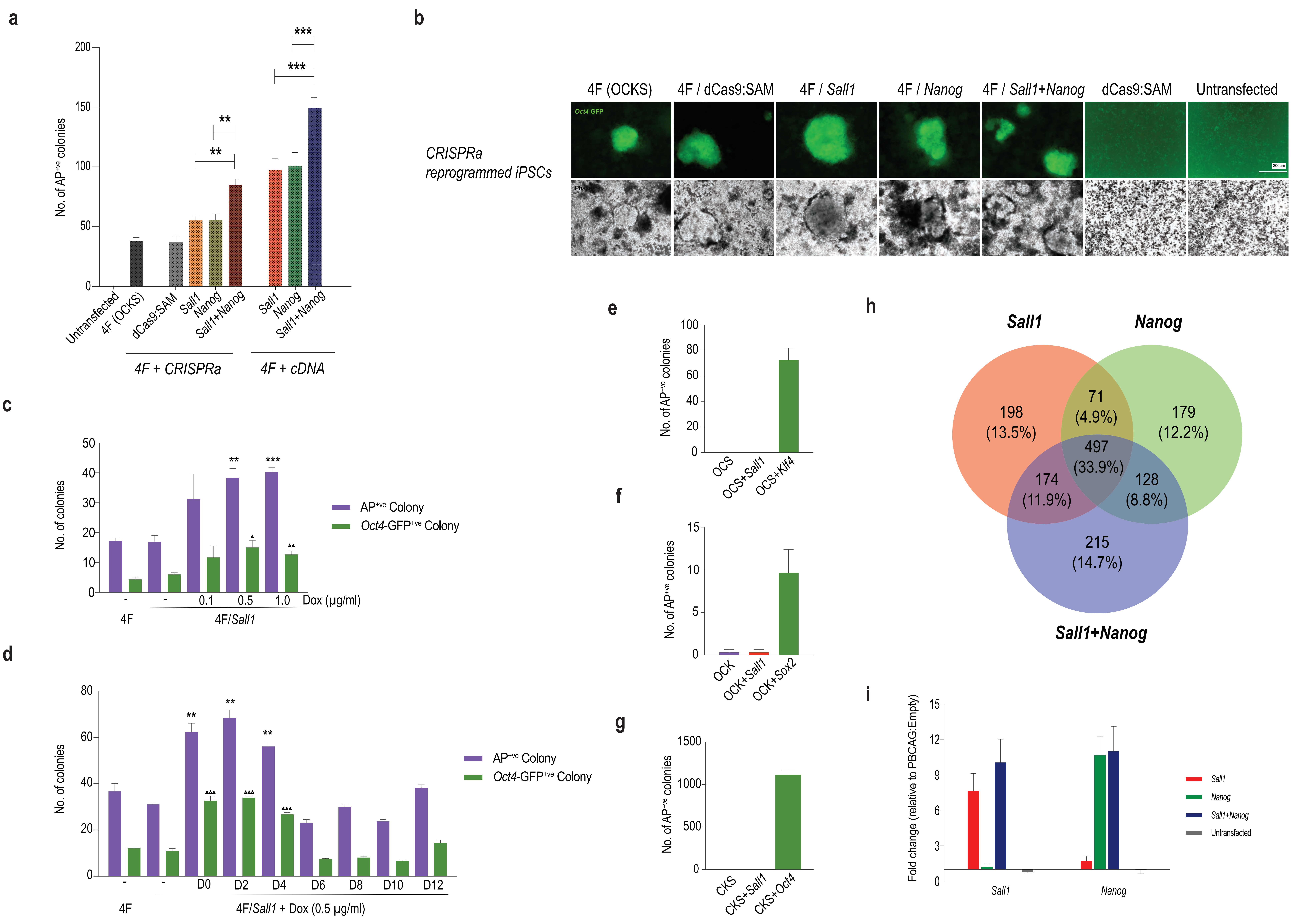

Supplementary Table S1

Primers for confirming *PiggyBac* mediated Gene integration (Genotyping).

Gene Name	Primer Name	Primer Sequence
Sall1-BPA	Sall1-BPA F	CAATCCTGTCAAGTTCCCAGAAAT
	Sall1-BPA R	CATCCCCAGCATGCCTGCTATT
Nanog-BPA	Nanog-BPA F	AGGGCTATCTGGTGAACGCATC
	Nanog-BPA R	AATCCTCCCCCTTGCTGTCCT
Oct4-c-Myc	Oct4-c-Myc F	GCCCCCAGGTCCCCACTTTG
	Oct4-c-Myc R	CCAGCTGATCGGCGGTGGAG
Klf4-Sox2	Klf4-Sox2 F	ACTATGCAGGCTGTGGCAA
	Klf4-Sox2 R	TTGCTGCGGGCCCGGCGGCT
Tet3G	Tet3G F	CCGTCCAGGCACCTCGATTAGTTC
	Tet3G R	GGTATGACTTGGCGTTGTTCC
Actb	Actb F	GTTTGAGACCTTCAACACCCC
	Actb R	GTGGCCATCTCCTGCTCGAAGTC
gRNA	pKLV_Flip_gRNA F	AGCAAAAAAAGCACCGACTCG
	pKLV_Flip_gRNA R	TAAAGCGCATGCTCCAGACTGC
dCas9:SAM	SamCas9 F	TTACTCAGTTCGTGCTCGTGGAC
	SamCas9 R	ATTGCCTTCACGATGAGTTCACA

Supplementary Table S2

Combinations of cDNA transfection used for MEF reprogramming. ***(Related to Figure 3 and Supplementary Figure S4)***

**Constructs**	**Combinations (amount of DNA in µg)**														
	1	2	3	4	5	6	7	8	9	10	11	12	13	14	15
PBCAG4F	1.0	1.0													
PBCAGCKS							1.0			1.0			1.0		
PBCAGOCK								1.0			1.0			1.0	
PBCAGOCS									1.0			1.0			1.0
PBCAG*Oct4*										1.0					
PBCAG*Klf4*												1.0			
PBCAG*Sox2*											0.5				
PBTRE4F			1.0	1.0	1.0	1.0									
PBTRE*Sall1*		0.5		0.5		0.5							0.5	0.5	0.5
PBTRE*Nanog*					0.1	0.1									
PBEF1-αTET3G		1.0	1.0	1.0	1.0	1.0							1.0	1.0	1.0
PiggyBac Transposase	2.0	2.0	2.0	2.0	2.0	2.0	2.0	2.0	2.0	2.0	2.0	2.0	2.0	2.0	2.0

Supplementary Table S3

Mouse RT-qPCR Probes for RNA Expression. ***(Related to Figures 2, 4 and Supplementary Figures S2, S3 and S4)***

Gene Name	Applied Biosystems Catalogue Number
Esrrb	Mm00442411_m1
Fgf5	Mm00438615_m1
Gapdh	4352339E
Gata6	Mm00802636_m1
Id3	Mm00492575_m1
Klf4	Mm00516104_m1
Nanog	Mm02384862_g1
Pou5f1	Mm00658129_gH
Rex1	Mm03053975_g
Sox2	Mm03053810_s1
Id2	Mm00711781_m1
Mdb2	Mm00521967_m1
Jarid2	Mm00445574_m1
Tet3	Mm00805756_m1
Tex10	Mm06549480_m1
Klf5	Mm00456521_m1
Smad7	Mm00484742_m1
Gp130	Mm00439665_m1
Sall1	Mm00491266_m1
Otx2	Mm0046859_m1
Fgfr1	Mm00438930_m1
Fam189a2	Mm01194369_m1
Lifr	Mm00442942_m1
Myc	Mm00487804_m1
Mycn	Mm00476449_m1
Arid2	Mm00558381_m1

Supplementary Table S4

Antibodies for Western Blotting / Immunofluorescence staining. ***(Related to Figure 3 and Supplementary Figure S2)***

Antibody	Company	Catalogue Number	Dilution
SSEA-1 Clone MC480	BD Pharmingen	560079	1:200
NANOG	Abcam	Ab80892	1:150
β III TUBULIN (TUJ1)	R & D Systems	MAB1159	1:150
α-Smooth Muscle Actin (SMA)	R & D Systems	MAB1420	1:150
α-Fetoprotein (AFP)	R & D Systems	MAB1368	1:150
H3K27ME3	Millipore	07-449	1:1000
OCT4 (C10)	Santa-Cruz	SC-5279	1:150 (IF)<br>1:800 (WB)
GAPDH	Sigma	G8795	1:4000
