# Supplementary material for: Genome-Scale CRISPRa Screen Identifies Novel Factors for Cellular Reprogramming
Source: Stem Cell Reports. 2019 Mar 21;12(4):757–71. doi: 10.1016/j.stemcr.2019.02.010 (PMC6450436; doi:10.1016/j.stemcr.2019.02.010)
Supplement: Document S2. Article plus Supplemental Information [file mmc6.pdf]

Stem Cell Reports
Article
ISSCR
OPEN ACCESS

# Genome-Scale CRISPRa Screen Identifies Novel Factors for Cellular Reprogramming

Jian Yang,[1,5,6] Sandeep S. Rajan,[1,2,6] Mathias J. Friedrich,[1,6] Guocheng Lan,[3] Xiangang Zou,[3] Hannes Ponstingl,[1] Dimitrios A. Garyfallos,[1] Pentao Liu,[1,4] Allan Bradley,[1] and Emmanouil Metzakopian[1,2,*]
[1]Wellcome Trust Sanger Institute, Genome Campus, Hinxton, Cambridgeshire CB10 1SA, UK
[2]UK Dementia Research Institute, Department of Clinical Neuroscience, Cambridge Biomedical Campus, University of Cambridge, Cambridge CB2 0AH, UK
[3]Cancer Research UK, Cambridge Institute, Li Ka Shing Centre, University of Cambridge, Cambridge CB2 0RE, UK
[4]School of Biomedical Sciences, Li Ka Shing Faculty of Medicine, Stem Cell and Regenerative Medicine Consortium, University of Hong Kong, Hong Kong, China
[5]Present address: Key Laboratory of Arrhythmias, Ministry of Education, Shanghai East Hospital, Tongji University School of Medicine, Shanghai 200120, China
[6]Co-first author
*Correspondence: em698@medschl.cam.ac.uk
https://doi.org/10.1016/j.stemcr.2019.02.010

## SUMMARY

Primed epiblast stem cells (EpiSCs) can be reverted to a pluripotent embryonic stem cell (ESC)-like state by expression of single reprogramming factor. We used CRISPR activation to perform a genome-scale, reprogramming screen in EpiSCs and identified 142 candidate genes. Our screen validated a total of 50 genes, previously not known to contribute to reprogramming, of which we chose *Sall1* for further investigation. We show that *Sall1* augments reprogramming of mouse EpiSCs and embryonic fibroblasts and that these induced pluripotent stem cells are indeed fully pluripotent including formation of chimeric mice. We also demonstrate that *Sall1* synergizes with *Nanog* in reprogramming and that overexpression in ESCs delays their conversion back to EpiSCs. Lastly, using RNA sequencing, we identify and validate *Klf5* and *Fam189a2* as new downstream targets of *Sall1* and *Nanog*. In summary, our work demonstrates the power of using CRISPR technology in understanding molecular mechanisms that mediate complex cellular processes such as reprogramming.

## INTRODUCTION

The ability of pluripotent stem cells (PSCs) to self-renew and their potential to differentiate into multiple cell types makes them useful for clinical applications (Martello and Smith, 2014). PSCs can either be derived from early embryos or be induced (iPSCs) by reprogramming somatic cells with Yamanaka factors, i.e., *Oct4*, *Sox2*, *c-Myc*, and *Klf4* among other transcription factors, mRNAs, microRNAs, and small molecules (Hou et al., 2013; Sandmaier and Telugu, 2015; Takahashi and Yamanaka, 2006; Warren et al., 2010). During early mouse embryo development, at least two types of PSCs can be derived, naive embryonic stem cells (ESCs) from the inner mass of the blastocyst and primed post-implantation epiblast stem cells (EpiSCs) (Nichols and Smith, 2009; Tesar et al., 2007). While both have the potential to differentiate into multiple lineages, only ESCs can contribute extensively to chimeras, showing unbiased developmental potential. Both ESCs and EpiSCs express major pluripotent transcription factors such as *Oct4* and *Sox2* at similar levels. In EpiSCs, however, reduced expression of pluripotency-associated factors such as *Rex1* and *Klf4* and elevated levels of early differentiation markers such as *Fgf5*, *Gata6*, and *Otx2* indicate their restricted developmental potential. Interestingly, EpiSCs cultured in fully defined ESC medium (with inhibition of *MAPK* and *GSK3* and supplementation with LIF; hereafter 2i/LIF medium) can be reprogrammed into ESCs by overexpressing only a single gene—such as *Nanog*, *Klf4*, or *Nr5a2* (Guo and Smith, 2010)—making them an ideal model system for genetic screens.

Recently, CRISPR/Cas9 has gained importance by achieving simple, precise, and rapid editing of the genome, enabling large-scale experiments such as genetic screening. While the RNA-programmable (single guide RNA [sgRNA]) endonuclease Cas9 is used to induce double-strand breaks in defined genomic locations, its catalytically dead variant (dCas9) can be fused with transcriptional activators and directed toward promoter regions to increase gene expression (CRISPR activation, CRISPRa) (Doudna and Charpentier, 2014; Gaj et al., 2013).

Genome-wide screening is a powerful unbiased approach to discover genes and pathways that underlie biological processes. To date, identification of key transcription factors and epigenetic modifiers within naive and primed PSCs has been investigated by employing either gain-of-function (GoF) screens using cDNA libraries and *PiggyBac* transposons or loss-of-function screens using RNA interference (Gayle et al., 2015; Hu et al., 2009; Pritsker et al., 2006).

Here, we describe the development and application of a genome-scale CRISPRa screen to identify genes that contribute to mouse EpiSC reprogramming. We show that our screening approach not only detects established reprogramming factors such as *Oct4* and *Nanog*, but also identifies previously unreported candidate genes capable of reprogramming. We focus on the role of *Sall1*, a


This is an open access article under the CC BY license (http://creativecommons.org/licenses/by/4.0/).

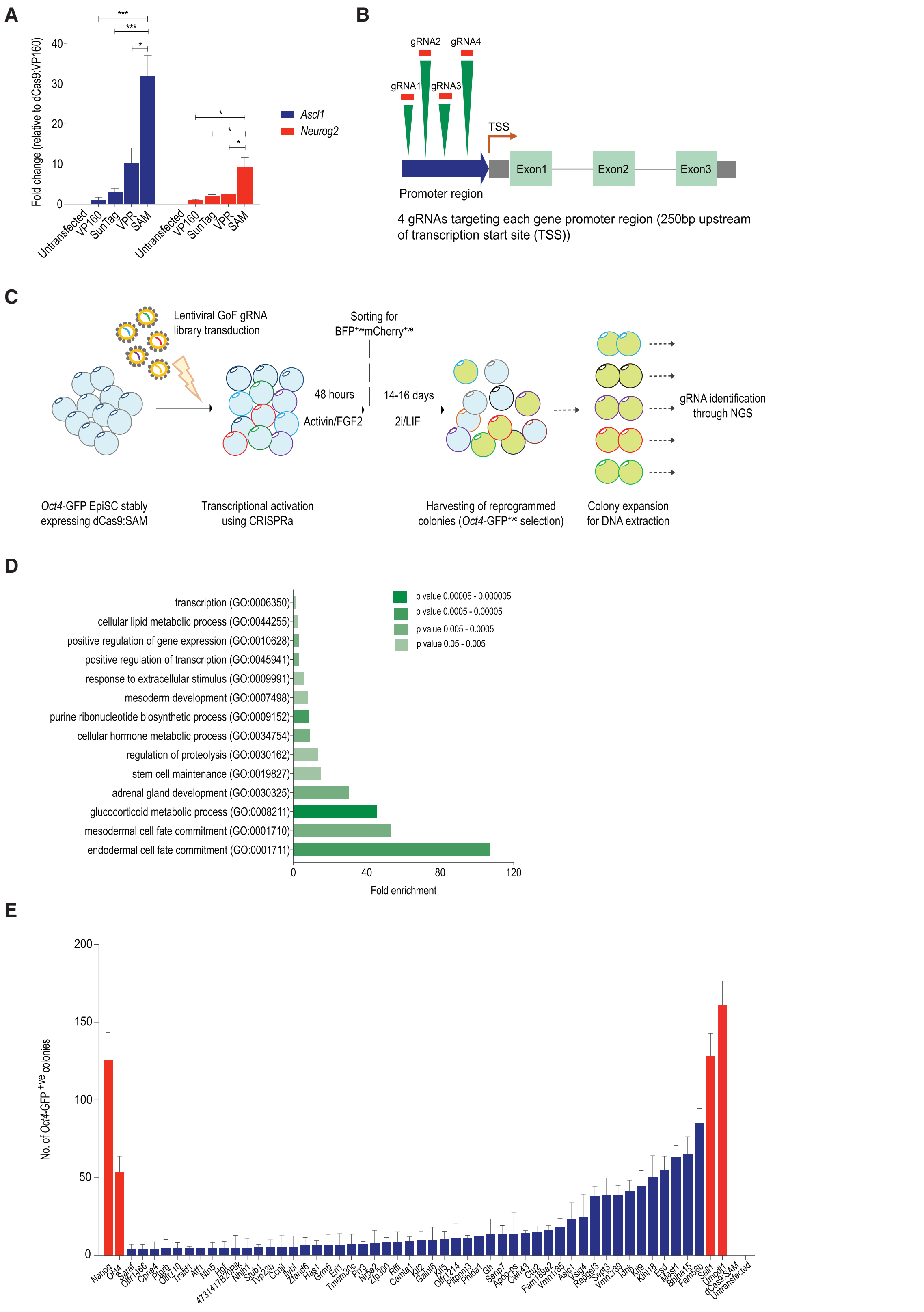


(legend on next page)

transcription factor belonging to the Spalt-like gene family, which has been implicated in cellular reprogramming in a number of studies but has not been sufficiently investigated (Basta et al., 2017; Gaspar-Maia et al., 2013; Mansour et al., 2012). Our work substantiates *Sall1* as a potent reprogramming gene candidate by demonstrating its ability to reprogram EpiSCs and mouse embryonic fibroblasts (MEFs) to iPSCs. In addition, we show that *Sall1* may exert its functions by interacting synergistically with *Nanog* to reprogram cells to ground state pluripotency.

## RESULTS

### GoF CRISPRa Screen Identifies Reprogramming Genes

Initially, we sought to determine the optimal Cas9 transactivation system, as several variants have been published (Chavez et al., 2016; Konermann et al., 2015; Tanenbaum et al., 2014). To that end, we created *PiggyBac*-transposable (Yusa et al., 2011) expression vectors with a Blasticidin-mCherry cassette for four different dCas9-CRISPRa systems: dCas:VP160, dCas9:SunTag, dCas9:VPR, and dCas9:SAM (Figure S1).

Furthermore, we designed a versatile sgRNA expression construct (pKLV-PB-U6-gRNA-PGK-Puro-T2A-TagBFP) (Metzakopian et al., 2017) with a selectable and a fluorescent marker (puromycin and BFP, Figure S1), which can be stably integrated into target genomes as lentivirus or via *PiggyBac*-mediated transposition (Yusa et al., 2011).

We directed single sgRNAs guides against the promoter region of *Ascl1* and *Neurog2*, genes with low baseline expression, in HEK293 cells. After stable integration of dCas9-CRISPRa and the sgRNA vectors via transposition and antibiotic selection, qRT-PCR revealed that dCas9:SAM achieved the highest overexpression of both target genes and thus was chosen for all subsequent experiments (Figure 1A).

To perform a genome-scale activation screen, we designed a pooled library of 87,863 sgRNAs targeting a 250-bp region upstream of the transcription start site (TSS) of 19,994 genes with an average of 4 guides each (Figure 1B; Table S5).

We decided to use EpiSC derived from *Oct4*-GFP reporter transgenic mice as they have been used for this purpose before (Yang et al., 2010). Characteristically for EpiSCs these cells already exhibit a baseline *Oct4* (and therefore GFP) expression. However, only cells successfully reprogrammed to the naive pluripotent state are able to maintain and increase *Oct4* expression upon plating in aforementioned 2i/LIF medium. Thus, successfully reprogrammed *Oct4*-GFP EpiSCs can be identified by their strong GFP expression (Figure S2A) and the characteristic ESC-like morphology, and grow as distinct colonies, whereas EpiSCs failing to reprogram either detach and die or differentiate.

We stably integrated dCas9:SAM into *Oct4*-GFP EpiSCs via *PiggyBac* transposition and then transduced $100 \times 10^6$ dCas9:SAM-expressing EpiSCs with our library at a MOI of 0.3 (Figure S2B). Two days later, we used fluorescence-activated cell sorting $10 \times 10^6$ to successfully transduce cells by BFP expression, giving a library coverage of around 114-fold. These BFP$^{+ve}$ cells were seeded in 2i/LIF medium to select for reprogramming cells. After 14–16 days of culture in 2i/LIF, 480 GFP$^{+ve}$ colonies were harvested for expansion (Figure 1C). Next-generation sequencing revealed 146 sgRNAs targeting 142 different genes (Table S6). These included known reprogramming factors *Nanog* (Mitsui et al., 2003), *Klf2* (Qiu et al., 2015), and *Nr5a2* (Guo and Smith 2010), confirming the specificity of the screen.

GOTERM analysis (Castro et al., 2011) on these 142 genes identified an enrichment in pathways related to transcriptional activation, expression of various transcription factors and enrichment toward stem cell maintenance (Figure 1D; Table S6).

To validate these candidate genes individually, we chose the highest performing sgRNA for each from the library, including *Nanog* as a positive control and again transduced dCas9:SAM-expressing *Oct4*-GFP EpiSCs. We expected the validation rate to be no higher than 50%, as small-scale single colony sub-sampling showed an average of two sgRNAs present in most colonies (data not shown), where one sgRNA presumably acts as the driver responsible for reprogramming, while the other is co-amplified as a passenger. As before, GFP$^{+ve}$ ESC-like colonies could be observed for *Oct4*,

**Figure 1. GoF EpiSC Reprogramming Screening with CRISPRa and sgRNA Library**

(A) Activation of *Ascl1* and *Neurog2* in HEK293 cells. Cells were transfected with one sgRNA per target and four different dCas9 versions. qRT-PCR normalized to *Gapdh*, fold change relative to dCas9:VP160 (mean of experimental triplicates ± SD, *$p < 0.05$; ***$p < 0.001$).

(B) sgRNA design targeting gene promoters in the murine genome.

(C) Screening strategy in *Oct4*-GFP EpiSCs stably expressing dCas9:SAM, lentiviral transduction (MOI = 0.3) of the sgRNA library. Reprogramming in 2i/LIF for 14–16 days, after sorting for transduced cells. NGS identified candidate sgRNAs in *Oct4*-GFP$^{+ve}$ iPSC colonies.

(D) GOTOOLBox analysis of 142 genes identified in GoF screening. Pathways with fold change compared with reference; colors indicate p values.

(E) Validation of 54 genes including *Nanog* and *Oct4* in dCas9:SAM-*Oct4*-GFP EpiSCs with single sgRNAs (*Oct4*-GFP$^{+ve}$ iPSC colonies, mean of 3 independent experiments ± SD).

See also Figures S1 and S2 and Tables S5, S6, and S7.

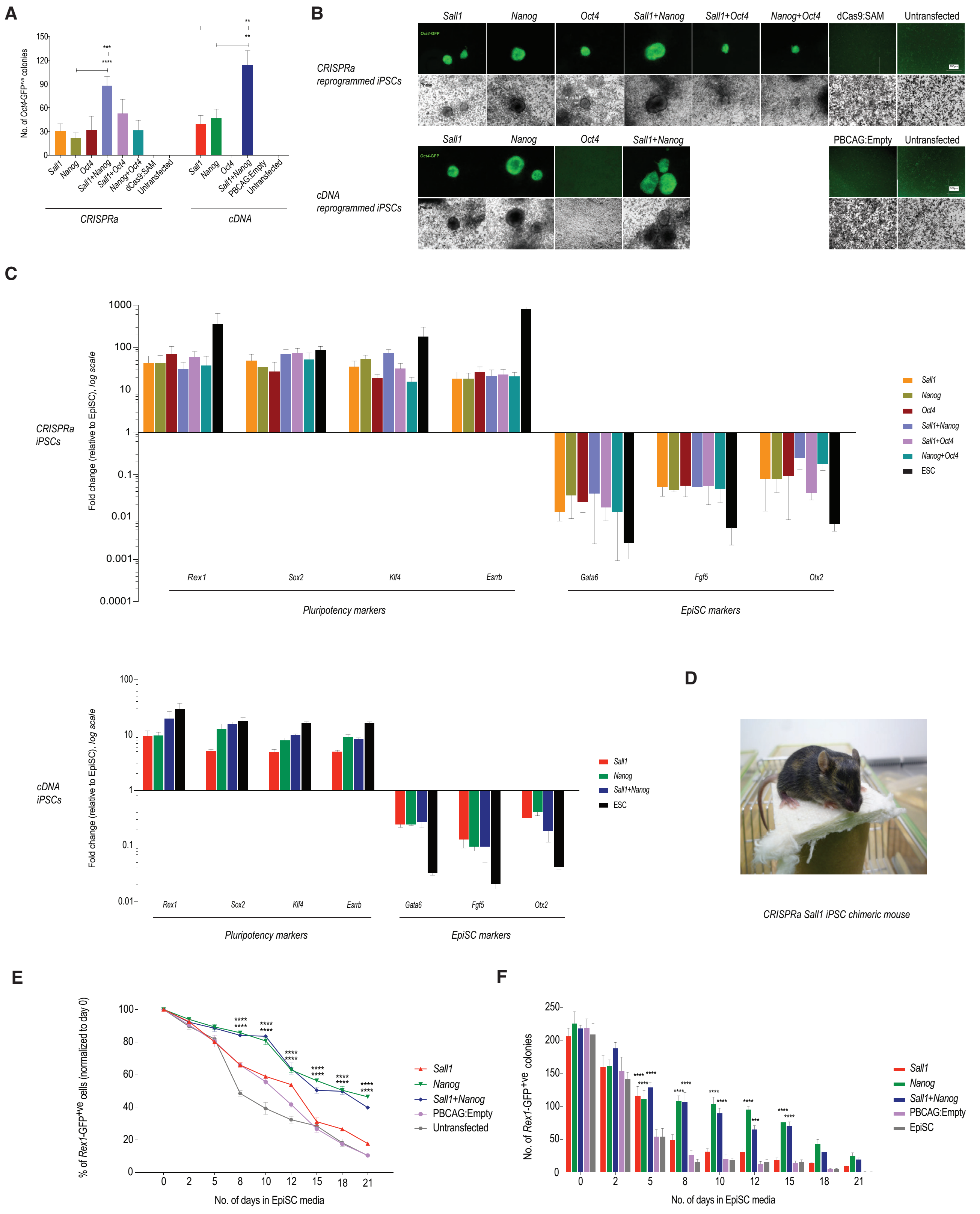


*(legend on next page)*

*Nanog*, and 52 of the candidate genes, resulting in a 36% validation rate (Table S7). The efficiency of reprogramming was gene dependent ranging from 5 to 165 colonies per $1 \times 10^6$ cells transfected (Figure 1E). Among the genes with the highest colony counts were positive controls *Nanog* and *Oct4*, as well as transcription factors *Klf2* and *Nr5a2* with a known role in reprogramming, confirming the validity of our CRISPRa approach.

## Gene Dosage Is Critical for *Oct4*-Mediated Reprogramming

We observed that CRISPRa-mediated induction of the pluripotency marker *Oct4* produced a significant number of ESC-like colonies, contradicting previous studies showing that cDNA-mediated *Oct4* expression is inefficient in EpiSC reprogramming (Guo and Smith, 2010; Niwa et al., 2000). Indeed, we were unable to generate any iPSC colonies in our EpiSCs with *Oct4* cDNA, while CRISPRa achieved robust reprogramming (Figure 2B).

We speculated that gene dosage might be the underlying issue and compared endogenous *Oct4* induction in EpiSCs with exogenous overexpression in more detail: compared with ESCs, CRISPRa-mediated induction of *Oct4* mRNA achieved roughly half the physiological expression level, while exogenous *Oct4* cDNA slightly surpassed it (Figure S2C). Doxycycline (Dox) titration of tet-inducible *Oct4* resulted in amounts comparable with *Oct4* cDNA and only very low concentrations of Dox gave levels similar to CRISPRa. Nevertheless, all cDNA-mediated overexpression conditions still failed to reprogram. On the protein level, all *Oct4* cDNA conditions produced disproportionally higher amounts than expected from the mRNA levels (Figure S2D, top panel). CRISPRa, on the other hand, achieved *Oct4* protein expression similar to that in ESCs (Figure S2D, bottom panel). We suspect that differences in mRNA stability are the cause, as CRISPRa-driven endogenous mRNA should be physiologically regulated, while exogenous mRNA could be more stable due to differing polyadenylation.

The importance of physiological expression levels agrees with our observation that, although our screening library contained an average of four sgRNAs per gene, almost all candidate genes from our screen were derived from only one specific sgRNA per target. Indeed, sgRNAs showed vastly different activities in a distribution that suggests a dependency on the distance of the sgRNA to the TSS (Figure S2E). This is also supported by a recent report (Liu et al., 2018a), which shows proof-of-principle MEF reprogramming using CRISPRa. In their experiments, only sgRNAs targeting the *Oct4* promoter in very specific locations (−71 and −127 bp from TSS) achieved activation sufficient for reprogramming, while in our experiments an sgRNA −101 bp from the TSS was successful.

## *Sall1* Facilitates EpiSC Reprogramming in Cooperation with *Nanog*

*Umodl1* and *Sall1* were the two most potent validated candidates from our screen. We confirmed that *Umodl1* upregulates *Lifr*, *Essrb*, *Nanog*, and *Sox2*, and downregulated *Tgfbr1* as would be expected in iPSC reprogramming when medium was switched from EpiSC to 2i/LIF (Figure S2F). *Sall1*, a member of the Spalt-family of transcription factors, has been reported to cooperate with *Nanog* to promote the maintenance of ESC state (Karantzali et al., 2011; Novo et al., 2016) and to play an important role in reprogramming and ESC differentiation (Basta et al., 2017; Mansour et al., 2012). However, the downstream targets of *Sall1* involved in reprogramming have not been sufficiently explored. Having found that *Sall1* is also able to independently reprogram EpiSCs, we set to investigate the underlying mechanisms.

First, we asked whether *Sall1* and *Nanog* also act synergistically in EpiSC reprograming by overexpressing them individually and in combination in *Oct4*-GFP EpiSCs. We performed these experiments both with CRISPRa as well as

**Figure 2. *Sall1* and *Nanog* Reprogram EpiSCs and Influence ESC Differentiation**

(A) Reprogramming efficiencies of *Sall1*, *Nanog*, and *Oct4* in *Oct4*-GFP EpiSCs stably transfected with CRISPRa or cDNA (*Oct4*-GFP$^{+ve}$ colonies, mean of 3 independent experiments ± SD, $**p < 0.01$; $***p < 0.001$; $****p < 0.0001$).

(B) Morphology of *Oct4*-GFP$^{+ve}$ colonies at day 20 in 2i/LIF is similar to ESC colonies. No colonies were observed in untransfected or mock transfected EpiSCs.

(C) qRT-PCR expression profiles of pluripotency markers and EpiSC markers in iPSC colonies normalized to *Gapdh* and relative to EpiSCs (mean of 3 independent experiments ± SD).

(D) Chimeric mouse produced with CRISPRa *Sall1*-induced PSCs injected into C57B/6 blastocyst.

(E) Flow cytometric analysis of *Rex1*-GFP$^{+ve}$ cells cultured in EpiSC medium at the timepoints indicated. Cells were stably transfected with *Sall1* or *Nanog* cDNA, or empty vector and cultured in EpiSC medium (mean of 3 independent experiments ± SD, $****p < 0.0001$ versus PBCAG:Empty).

(F) Number of *Rex1*-GFP$^{+ve}$ ESC colonies recovered after ESCs were converted in EpiSC medium at indicated timepoints (600 cells plated at time point zero; mean of 3 independent experiments ± SD, $***p < 0.001$; $****p < 0.0001$ versus PBCAG:Empty).

See also Figure S3 and Table S2.

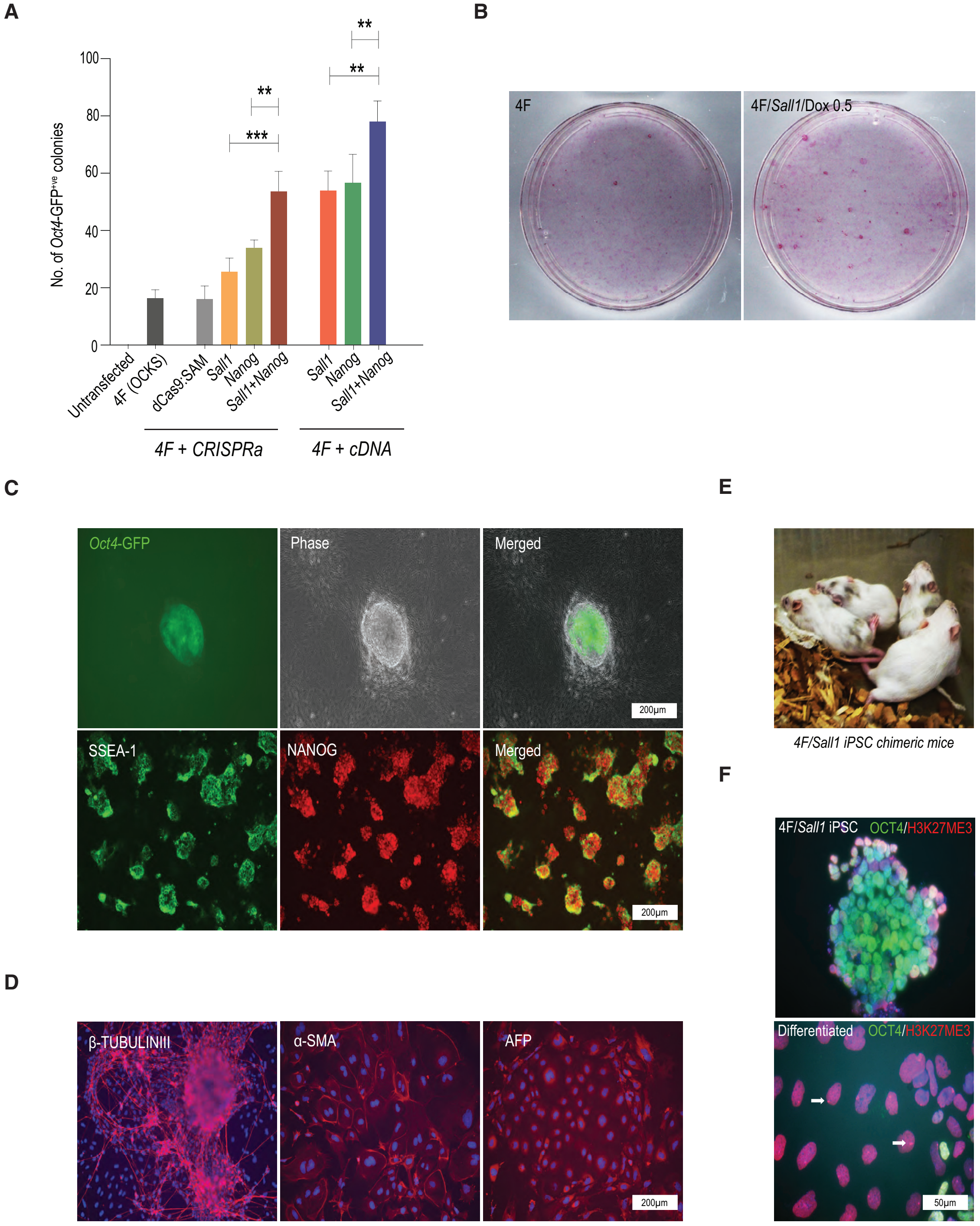


**Figure 3. CRISPRa Gene Induction and cDNA-Mediated Overexpression of *Sall1*, *Nanog* Reprogrammed MEF to iPSCs**

(A) (4F + CRISPRa) MEFs stably transfected with CAG4F and gRNAs against *Sall1*/*Nanog*/*Sall1*+*Nanog* in ESC medium (*Oct4*-GFP$^{+ve}$ colonies after 18 days; mean of 3 independent experiments ± SD, $^{**}p < 0.01$; $^{***}p < 0.001$) (4F + cDNA) MEFs stably transfected with TRE4F, TRENanog, and TRESall1 (all co-transfected with PBEF-1αTet3G), induced with 0.5 µg/mL Dox for 12 days and counted on day 18 (mean of 3 independent experiments ± SD, $^{**}p < 0.01$).

(B) Alkaline phosphatase-positive (AP$^{+ve}$)-stained ESC colonies reprogrammed from MEFs by 4F alone and in combination with *Sall1* (induced with Dox at 0.5 µg/mL).

*(legend continued on next page)*

cDNA-mediated overexpression, and also verified that the observed activity of *Sall1*-specific sgRNA was not due to cross-reactivity with *Sall4*, a known pluripotency factor (Figure S2G).

Three days after transfection, qRT-PCR showed a 2.5- to 3.5-fold increase in expression of *Sall1*, *Nanog*, and *Oct4* mediated by CRISPRa and a 10- to 20-fold increase in expression through cDNA (Figure S3A). Co-expression of *Sall1* and *Nanog* resulted in a marked increase in *Oct4*-GFP$^{+ve}$ ESC-like colony numbers (Figures 2A and 2B). As above, *Oct4* induction via CRISPRa successfully reprogrammed EpiSCs (but not cDNA overexpression), without showing significant synergy with either *Sall1* or *Nanog*. Pluripotency markers examined by qRT-PCR (*Rex1*, *Sox2*, *Klf4*, and *Essrb*) were markedly increased; concordantly, EpiSC markers *Gata6*, *Fgf5*, and *Otx2* showed decreased expression (Figure 2C). *Sall1* reprogrammed EpiSCs (MF1 and C57BL/6 background) contributed significantly to chimeras when injected into C57BL/6 blastocysts (Figures 2D and S3E).

To exclude the possibility that the baseline GFP expression of the *Oct4*-GFP reporter EpiSCs might skew the correct identification of successfully reprogrammed EpiSCs, we repeated these experiments with *Nanog*-GFP reporter EpiSCs (Yang et al., 2010), which show strong GFP expression on successfully entering the naive ESC state, but virtually none in the primed EpiSC state (Guo and Smith, 2010). Both gene induction using CRISPRa and overexpression via cDNA confirmed the reprogramming capability of *Sall1* alone and in synergy with *Nanog* (Figures S3B–S3D). Notably, colony formation assays in 2i/LIF recapitulated the results obtained with *Oct4*-GFP EpiSCs and the reprogramming kinetics as measured in time course experiments were comparable between the two reporter cell lines (Figure S3F).

## *Sall1* and *Nanog* Delay Differentiation of ESCs into EpiSCs

ESCs readily differentiate into EpiSCs in culture medium containing the EpiSC self-renewal factors Activin and fibroblast growth factor 2 (FGF2) (Guo et al., 2009). To investigate whether higher levels of *Sall1* and *Nanog* can delay this conversion we generated stable cDNA transfectants in *Rex1*-GFP ESCs (Wang et al., 2011). We cultured the cells in EpiSC media and quantified the *Rex1*-GFP$^{+ve}$ population as a measure of cells remaining in the ESC ground state in a 21-day time course. *Nanog* and *Sall1+Nanog* maintained a significantly higher proportion of GFP$^{+ve}$ cells than *Sall1* (Figure 2E). The expression of naive pluripotency and EpiSC markers analyzed by qRT-PCR followed a similar pattern (Figures S3G–S3I), although *Sall1* delayed upregulation of differentiation markers *Fgf5* and *Otx2*. Concordantly, when plated in 2i/LIF medium, *Nanog* and *Sall1+Nanog* overexpressing cells retained the ability to form ESC colonies through most of the time course, and *Sall1* preserved colony formation capacity until after 6 days (Figure 2F). While *Sall1* might not have the same capacity as *Nanog* to keep the ESC ground state, it may confer a longer "formative state" (Smith, 2017) during conversion.

## *Sall1* Promotes MEF Reprogramming and Works Synergistically with *Nanog*

To test whether *Sall1* enhances somatic cell reprogramming, we stably transfected *Oct4*-GFP reporter MEFs (*Oct4*-GFP-MEFs) with the Yamanaka factors (CAG4F, Figure S1), dCas9:SAM and sgRNAs against *Sall1* and/or *Nanog*. In ESC media, *Sall1* sgRNA transfected MEFs produced a significantly higher number of *Oct4*-GFP$^{+ve}$ and alkaline phosphatase-positive (AP$^{+ve}$) colonies (Figures 3A and S4A) with ESC-like morphology (Figure S4B) than CAG4F alone, mirroring the results obtained from EpiSC reprogramming, including synergy between *Sall1* and *Nanog*.

To examine the dynamics of MEF reprogramming, we co-transfected *Oct4*-GFP-MEFs with tet-inducible *Sall1* (TRESall1, Figure S1; Table S2) and CAG4F, and induced expression with three concentrations of Dox (0.1, 0.5, and 1.0 μg/mL) to find a suitable concentration to mediate reprogramming in ESC medium. After 18 days, Dox concentrations of 0.5 or 1.0 μg/mL resulted in a significant 2- to 3-fold increase in *Oct4*-GFP$^{+ve}$ and AP$^{+ve}$ colonies (Figures 3B and S4C) and we chose 0.5 μg/mL Dox for all subsequent experiments. To determine the active window for *Sall1*, we induced expression at 0, 2, 4, 6, 8, 10, and 12 days of reprogramming and found that only activation during the first 4 days resulted in higher reprogramming efficiency (Figure S4D).

As *Nanog* has been reported to promote MEF reprogramming (Theunissen et al., 2011) we tested for synergy with

(C) iPSCs reprogrammed from C57B/6J MEF with 4F/Sall1. *Oct4*-GFP expression and ESC-like morphology (upper panel), immunofluorescent staining for pluripotency markers SSEA-1 and NANOG (lower panel).
(D) *In vitro* differentiation of C57B/6 MEF reprogrammed iPSCs with 4F/Sall1; neuronal differentiation in N2B27 (immunofluorescence for β-tubulin III$^{+}$); mesoderm and endoderm differentiation in M10 (alpha smooth muscle actin [α-SMA] and alpha fetoprotein [AFP] antibody staining).
(E) Chimeric mice produced with 4F/Sall1-iPSCs injected into CD1 blastocysts.
(F) Inactivation of X chromosomes in female 4F/Sall1-iPSCs (co-immunostaining for H3K27me3 and *Oct4*. Arrows indicate H3K27me3 foci).
See also Figure S4 and Tables S2 and S3.

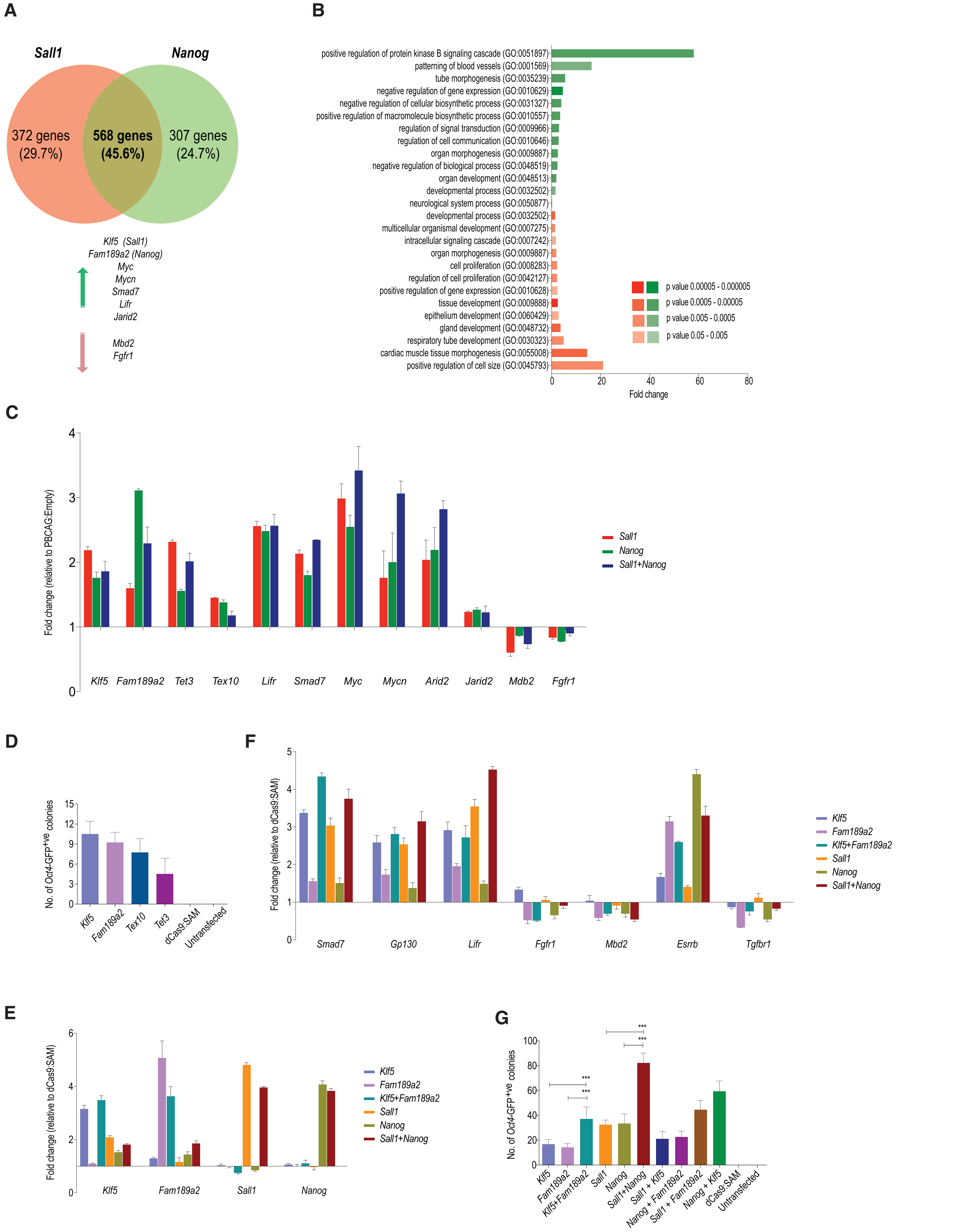


*(legend on next page)*

*Sall1* by transfecting MEFs with tet-inducible Yamanaka factors, *Nanog* and *Sall1* (TRE4F, TRENanog, and TRESall1). Indeed, co-expression of *Sall1*+*Nanog*/4F led to a 1.5-fold increase in colony number compared with either factor alone (Figures 3A and S4A).

The *Sall1*-iPSCs derived from these experiments were morphologically similar to ESCs with a compact dome-like structure and *Oct4*-GFP expression. Immunofluorescent staining of these iPSCs showed protein expression of the ESC-markers SSEA-1 and NANOG (Figure 3C). In differentiation medium (DMEM/10% fetal calf serum [FCS]) or N2B27 medium (Ying et al., 2003), these iPSCs exited ground state pluripotency and differentiated into mesoderm, endoderm, and ectoderm lineages as confirmed by immunofluorescent staining for expression of smooth muscle actin, alpha fetoprotein, and β-tubulin III (Figure 3D). In addition, when we injected these iPSCs into blastocysts, live chimeras were born (Figure 3E), confirming the pluripotency of these *Sall1*-iPSCs.

Female mESCs have two activated X chromosomes when maintained at ground state (Lessing et al., 2013) and randomly inactivate one of them once they undergo differentiation. Staining with anti-H3K27me3 antibody detects this event as foci on the inactivated X chromosome (Silva et al., 2008). We derived iPSCs from female MEFs by co-transfecting with 4F/*Sall1* as before and then differentiated them in DMEM/10% FCS for 5 days. Loss of *Oct4* expression demonstrated successful differentiation and the presence of H3K27me3 foci indicated X chromosome silencing. In contrast iPSC cultured in 2i/LIF strongly expressed *Oct4* protein and lacked any H3K27me3 foci (Figure 3F).

Together, this demonstrates that *Sall1* can enhance 4F-driven somatic cell reprogramming and that 4F/*Sall1* reprogrammed iPSCs are naive and pluripotent.

Yamanaka factors *Oct4*, *Sox2*, and *Klf4* are essential and sufficient for reprogramming, albeit at a lower efficiency than in conjunction with *c-Myc*; all three can be replaced by other transcription factors or small molecules such as *Gata3* (Shu et al., 2013) or valproic acid (Biswas and Jiang, 2016; Huangfu et al., 2008). However, in co-transfection experiments, *Sall1* was unable to substitute for any of the factors in MEFs (Figures S4E–S4G).

### RNA Sequencing Identifies Potential Mechanisms of Cellular Reprogramming Mediated by *Sall1* and *Nanog*

We performed RNA sequencing (RNA-seq) for *Oct4*-GFP EpiSCs overexpressing *Sall1* and/or *Nanog* via cDNA for 24 h. Our analysis identified 372 genes differentially expressed specific to *Sall1*-transfected cells, and 307 genes specific to *Nanog*. We observed a large overlap of 568 genes (45%) between both sets (Figure 4A; Table S8) and GOTERM analysis (Castro et al., 2011) revealed that they are involved in a number of developmental processes and signaling cascades (Figure 4B; Table S8).

Among those commonly regulated genes were *Myc*, *Mycn*, *Tet3*, *Tex10*, *Jarid2*, *Fgfr1*, *Mbd2*, *Lifr*, and *Smad7* (Figure 4A) which have previously been implicated in the promotion of cellular reprogramming or inhibition of ESC differentiation (Li et al., 2016; Iseki et al., 2016; Bagci and Fisher, 2013; Fidalgo et al., 2016; Jinek et al., 2012; Niwa et al., 1998; Hall et al., 2009; Festuccia et al., 2017). Upregulation of *Lifr* and downregulation of *Fgfr1* is expected in EpiSC reprogramming and validates our RNA-seq and qRT-PCR data (Figure 4C). Furthermore, we found 215 genes that were only regulated when *Sall1* and *Nanog* were overexpressed together (Figure S4H; Table S8), such as *Dnmt3c* and *Hdac9*, reported to be involved in the epigenetic regulation of male germ cell maintenance (Barau et al., 2016) and muscle differentiation (Mihaylova and Shaw, 2013), respectively; as well as a modest upregulation of *Utf1*, a transcription factor known to synergize

**Figure 4. RNA-Seq Identifies Potential Mechanisms of Reprogramming Mediated by Sall1 and Nanog**

(A) Venn diagram of genes being differentially expressed in *Sall1* and *Nanog* overexpressing cells (cutoff $p_{adj} < 0.001$). Upregulated (green arrow) and downregulated (red arrow) genes for further experiments were chosen from the overlap between *Sall1* and *Nanog*, except *Klf5* and *Fam189a2*.

(B) GOTOOLBox analysis of common regulated genes (fold change compared with reference, colors indicate p values).

(C) qRT-PCR validations of RNA-seq (24 h after transfection, normalized to *Gapdh* and relative to PBCAG:Empty; mean of 3 independent experiments ± SD).

(D) Reprogramming of *Oct4*-GFP EpiSCs via CRISPRa-mediated gene induction of *Klf5*, *Fam189a2*, *Tex10*, and *Tet3* (*Oct4*-GFP$^{+}$ colonies after 2i/LIF for 20 days; mean of 3 independent experiments ± SD).

(E) qRT-PCR for *Klf5*, *Fam189a2*, *Sall1*, and *Nanog* after CRISPRa-mediated induction of *Klf5* and *Fam189a2* (flow-sorted for sgRNA expression after 24 h, normalized to *Gapdh* and relative to dCas9:SAM; mean of 3 independent experiments ± SD).

(F) qRT-PCR expression levels of key regulators in JAK/STAT3 and TGF-β signaling (flow-sorted for sgRNA expression 24 h after changing to 2i/LIF media, 48 h after transfection, normalized to *Gapdh* and relative to dCas9:SAM; mean of 3 independent experiments ± SD).

(G) Reprogramming of *Oct4*-GFP EpiSCs via CRISPRa-mediated gene induction of *Klf5*, *Fam189a2*, *Sall1*, and *Nanog* (*Oct4*-GFP$^{+ve}$ colonies after 20 days in 2i/LIF; error bars represent mean of 3 independent experiments ± SD).

See also Figure S4 and Tables S2 and S8.

with the Yamanaka factors in reprogramming (Zhao et al., 2008).

We had already independently identified the genes *Klf5* and *Fam189a2* in our GoF screen (Figure 1E; Table S5) and RNA-seq showed them to be potentially regulated by *Sall1* and *Nanog*, respectively. We validated the RNA-seq results with qRT-PCR and found a good correlation between both methods (Figures 4C and S4I). While *Klf5* narrowly failed the stringent p value cutoff for the RNA-seq results in *Nanog* overexpressing cells, qRT-PCR indicated that *Klf5* may be regulated by *Nanog* as well, albeit to a lesser extent than by *Sall1*. *Fam189a2* on the other hand seemed to be regulated significantly stronger by *Nanog* than *Sall1*. When we co-expressed *Sall1* and *Nanog*, we did not observe a significant synergistic increase of expression for these downstream targets (Figure 4C); we did, however, for the genes *Myc*, *Mycn* (Chappell and Dalton 2013), and *Arid2*, all of which have been shown to play a role in reprogramming and chromatin remodeling (Awe and Byrne, 2013; Singhal et al., 2010).

We used CRISPRa to induce expression of *Klf5*, *Fam189a2*, *Tex10*, and *Tet3* in *Oct4*-GFP EpiSCs and found that all were able to augment reprogramming into iPSCs (Figure 4D). Reprogramming by *Fam189a2* occurred in 10 days, while *Klf5*, *Tex10*, and *Tet3* required between 14 and 20 days. In all cases, the number of reprogrammed colonies was significantly lower compared with *Sall1* or *Nanog* (Figure 4G), which may indicate that multiple downstream targets of *Sall1* and *Nanog* participate in reprogramming.

We tested the regulatory relationship between *Sall1+Nanog* and *Klf5+Fam189a2* by transfecting EpiSCs with CRISPRa for *Klf5* and *Fam189a2*. Transcription increased significantly for *Klf5* and *Fam189a2*, but not for *Sall1* and *Nanog*, indicating *Klf5* and *Fam189a2* are downstream targets (Figure 4E). qRT-PCR for some of the key genes differentially regulated in the RNA-seq data showed that both *Sall1* and *Klf5* upregulated *Smad7*, *Gp130*, and *Lifr*, suggesting the repression of transforming growth factor β (TGF-β) signaling and activation of Jak/Stat3 signaling. *Nanog* and *Fam189a2* on the other hand downregulated *Fgfr1*, *Tgfr1*, and *Mbd2* and upregulated *Esrrb* expression, indicating the repression of FGF and TGF-β signaling, inhibition of epigenetic repression and promotion of self-renewal and pluripotency (Figure 4F). Functionally, co-activation of both *Klf5* and *Fam189a2* generated significantly more *Oct4*-GFP$^{+ve}$ colonies than either gene alone. As expected, co-activation of either *Sall1* and its downstream target *Klf5* or *Nanog* and its downstream target *Fam189a2* showed no synergistic effects in *Oct4*-GFP$^{+ve}$ colony production, whereas co-activation of either *Sall1* and *Fam189a2* or *Nanog* and *Klf5* did, although less than *Sall1* and *Nanog* co-activation. These results suggest that *Klf5* and *Fam189a2* are situated downstream of *Sall1* and *Nanog*, respectively, and can synergize as well (Figure 4G).

## DISCUSSION

To date few CRISPR activation screens have been performed (Bester et al., 2018; Heaton et al., 2017; Liu et al., 2018b) using previously established GoF libraries (Kampmann, 2018; Konermann et al., 2015). However, none of them targeted stem cell reprogramming and, while some recent publications have used CRISPRa in this field of research, they have been restricted to a few genes to demonstrate proof-of-concept (Guo et al., 2017; Liu et al., 2018a; Weltner et al., 2018).

Our present study shows that a genome-scale CRISPRa screen, in conjunction with an experimental model such as EpiSCs, in which a single overexpressed gene may mediate reprogramming to pluripotency, is a powerful tool for gene discovery. We identified 142 candidate reprogramming factors, among them *Nanog*, known to reprogram EpiSCs to iPSCs (Silva et al., 2009), validating our screen. Similarly, we found the Yamanaka factor *Oct4* (Takahashi and Yamanaka, 2006), which is critical for the maintenance of ESCs and differentiation (Niwa et al., 2000). Curiously, CRISPRa-induced *Oct4* readily and robustly reprogrammed EpiSCs into iPSCs, while overexpression via cDNA failed in our experiments and those of others (Guo and Smith, 2010). We reason that gene dosage is one critical aspect to explain this behavior and that excessive levels of *Oct4* can be detrimental to pluripotency, which tallies with previous studies suggesting that artificially reduced *Oct4* levels maintain ESCs in a robust pluripotent state, whereas wild-type levels enable differentiation (Gao et al., 2013; Karwacki-Neisius et al., 2013; Radzisheuskaya et al., 2013).

The important implication for CRISPRa-mediated screens is that tiled sgRNAs in regulatory regions of genes can, as we and others show (Liu et al., 2018a), provide a variety of expression levels unachievable with exogenous cDNA, giving a higher probability of matching the physiological gene dosage. Conceivably, the choice of the CRISPRa system may well influence the outcome of a screen and repeating our screen with a different CRISPRa system at lower activation efficiencies than SAM could produce a non-redundant list of candidate genes.

While the positional aspect of sgRNA efficiency certainly serves to explain why most of our candidate genes were only identified by a single sgRNA in our screen, we also acknowledge that reprogramming is inherently a very inefficient process and, thus, a very large initial cell number may be required to cover a genome-wide library deeply enough to give a sufficient number of cells a chance to

gain pluripotency. While we performed our GoF screen with 10 × $10^6$ library-transduced cells (library coverage 114×), a deeper coverage or a more focused library promises to uncover reprogramming candidates the present screen might have missed.

Our screen identified *Sall1*, a member of the Spalt-like gene family, as a potent EpiSC reprogramming factor. *Sall1* and *Sall4* have been implicated in the establishment of pluripotency (Gaspar-Maia et al., 2013) in studies showing that the action of demethylase *Utx* on *Sall1* and *Sall4* is required to enable MEF reprogramming (Mansour et al., 2012). Furthermore, it has been demonstrated that *Sall4* activates *Oct4* expression while *Sall1* is a direct binding partner of *Nanog* (Karantzali et al., 2011; Zhang et al., 2006) and has been suggested to be required in *Nanog*-mediated open heterochromatin maintenance within ESCs and EpiSCs (Novo et al., 2016). So far, it is unclear whether *Sall1* plays an active role in EpiSC reprogramming. In our work, we demonstrate that endogenous as well as exogenous *Sall1* can reprogram EpiSCs, and that *Sall1* synergizes with *Nanog* in reprogramming EpiSCs and MEFs. However, *Sall1* cannot replace *Oct4*, *Sox2*, or *Klf4* in MEF reprogramming, suggesting that it is unable to initiate the reprogramming machinery in more differentiated cells. One of its roles may be in facilitating epigenetic modification and nucleosome remodeling, e.g., through interaction with *Nanog* and the deacetylase complex (NurD) (Basta et al., 2017).

Unlike *Nanog*, the ability of *Sall1* to reprogram EpiSCs is insufficient to keep ESCs in the naive pluripotent state, only marginally delaying loss of pluripotency in differentiation experiments. However, it slowed expression of EpiSC markers *Fgf5* and *Otx2*, and preserved the ability to generate ESC-like colonies. *Sall1* inhibited *Otx2* expression in embryoid body differentiation of ESCs, and a formative pluripotent phase between naive and primed states was postulated when cells lost naive pluripotency markers and gained post-implantation markers such as *Otx2* and *Oct6* among others (Karantzali et al., 2011; Smith, 2017). Considering that, even after 21 days in differentiation medium, some *Sall1* overexpressing cells still formed ESC-like colonies in 2i/LIF, these cells may be stalled in a formative state.

We used RNA-seq to identify downstream targets of *Sall1* and *Nanog* in EpiSCs and found genes previously implicated in pluripotency or stem cell maintenance. *Esrrb*, a downstream target of *Nanog*, plays an important role in maintaining ESCs pluripotency and reprogramming by interacting with the core pluripotency network via *Sox2* (Adachi et al., 2013). *Tex10* was recently reported to be a pluripotency factor and partner of *Sox2*, capable of promoting MEF reprogramming (Ding et al., 2015), a role we further extended to EpiSC reprogramming. *Tet3* is a member of the ten-eleven translocation (Tet) protein family, which regulate DNA methylation. *Tet1* and *Tet2* have already been implicated in somatic reprogramming and *Tet2* has been reported to promote EpiSCs to a naive state (Bagci and Fisher, 2013; Fidalgo et al., 2016). Here, we show that *Tet3* can mediate EpiSC reprogramming as well.

The Kruppel-like factor family proteins *Klf2*, *4*, and *5* are also pluripotency factors and both *Klf2* and *Klf4* have been shown to facilitate reprogramming (Jeon et al., 2016). The potential of *Klf5* however is unclear as it has been reported to be incapable of reprogramming EpiSCs in a study by Hall et al. (2009), while Jeon et al. (2016) and recently Azami et al. (2018) both were able to derive iPSCs from EpiSCs by cDNA-mediated *Klf5* overexpression. We identified *Klf5* in our GoF screen and confirmed its ability to reprogram EpiSCs via CRISPRa transcriptional activation. These incongruent observations may reflect a Goldilocks effect similar to our observations with *Oct4*, highlighting the utility of different overexpression approaches to discover new pluripotency factors. LIF-dependent activation of Jak/Stat3 and its role in ESC self-renewal and reprogramming has been widely studied to date (Tang and Tian, 2013; Yu et al., 2017). Overexpression of *Klf5* via cDNA may compensate for the absence of LIF in maintaining ESC pluripotency and thereby be capable of reprogramming EpiSCs via LIF-independent pathways (Ema et al., 2008; Jeon et al., 2016). Besides *Klf5*, our data also indicate that *Sall1* positively and negatively regulates the Jak/Stat3 and TGF-β pathways, respectively (via *Gp130*, *Lif* receptor, and *Smad7*), together providing insights into the role of *Sall1* in EpiSC reprogramming.

*Fam189a2* was identified as a new target of *Nanog* in EpiSC reprogramming and our data showed that both *Nanog* and *Fam189a2* downregulate *Tgfbr1* and upregulate *Esrrb* expression. We postulate that the observed synergy between *Sall1* and *Nanog* as well as their downstream effectors *Klf5* and *Fam189a2* is partially due to the combined activation of Jak/Stat3, suppression of TGF-β signaling and upregulation of pluripotent genes such as *Esrrb*.

In conclusion, using a genome-scale CRISPR activation screen in the well-established EpiSCs reprogramming model, we identify known and previously unknown genes that can mediate cellular reprogramming in EpiSCs. We demonstrate that the transcription factor *Sall1* can effectively reprogram EpiSCs and MEFs, and provide new insights into the role of *Sall1* in promoting and maintaining pluripotency. Other reprogramming candidates such as transcription factors *Atf1* and *Bhlha15*, kinases *Idnk* and *Has1*, several olfactory receptor genes (*Olfr*), and others with less known functions such as *Umodl1* and *Prr3* deserve further in-depth investigation. Our studies demonstrate the strengths of CRISPR activation screens in the identification of factors that were previously not reported in

molecular reprogramming and in illuminating biological pathways.

## EXPERIMENTAL PROCEDURES

### GoF gRNA Library Design

In brief, the GoF library targeted the region of up to 250 bp upstream of the TSS of each protein-coding gene. Up to 4 guides of 19 bp length were selected per gene. Guide sequences with off-target sites exhibiting fewer than 3 mismatches over their 19 bp length were omitted from the design.

A selection algorithm was designed to spread high-quality guides across the target region. To this end, the region upstream of the TSS was divided into quarters of roughly equal length. Starting with the quarter closest to the TSS the algorithm looped over quarters picking the best guide, by quality score, in each if available, and adding it to the library until no more guide fitting the constraints could be found, or the target number of five guides per genes was reached. A constraint for the GC content of less than 55% was applied in the first loop and then relaxed to 70%.

### GoF Reprogramming Screen

The GoF sgRNA library was synthesized by Custom Array, and the oligo pools were cloned into the lentiviral sgRNA expression plasmid via Gibson assembly as described by Shalem et al. (2014), with minor modifications.

In brief, *Oct4*-GFP EpiSC cells stably expressing dCas9:SAM were first generated and were expanded to 100 × $10^6$ cells for lentiviral transduction of the GoF library. Library transduction was carried out at an MOI of 0.3. After 2 days, 10 × $10^6$ BFP$^{+ve}$ *Oct4*-GFP EpiSCs were sorted by flow cytometry and plated in 2i/LIF in order to allow selection for reprogrammed cells. After 14–16 days in 2i/LIF, the individual reprogrammed colonies, verified by *Oct4*-GFP fluorescence, were picked and transferred to 96-well plates for colony expansion and genomic DNA extraction. PCR amplification on the genomic DNA, across the stably integrated sgRNA, was performed using primers described previously (Koike-Yusa et al., 2014) and NGS was used to identify the sgRNA sequences.

All experimental procedures are detailed out in the Supplemental Information.

## ACCESSION NUMBERS

The accession number for the data reported in this paper is ArrayExpress: E-MTAB-7692.

## SUPPLEMENTAL INFORMATION

Supplemental Information can be found online at https://doi.org/10.1016/j.stemcr.2019.02.010.

## AUTHOR CONTRIBUTIONS

J.Y. initiated and designed the project, performed GoF screening and EpiSC, MEF reprogramming with cDNA, analyzed the data, and wrote the manuscript. S.S.R. performed EpiSC reprogramming, ESC conversion, MEF reprogramming with gRNA, RNA-seq, analyzed the data, and wrote the manuscript. M.J.F. performed EpiSC reprogramming and RNA-seq data analysis, and wrote the manuscript. G.L. and X.Z. performed microinjection and analyzed chimera data. H.P. designed the GoF sgRNA library and performed RNA-seq data analysis. D.A.G. performed experiments to determine various dCas9-mediated overexpression levels. P.L. and A.B. financially supported the project and interpreted the data. E.M. initiated, designed, and supervised the project, performed GoF screening, validation, analyzed and interpreted data, and wrote the manuscript.

## ACKNOWLEDGMENTS

We thank Caroline Sinclair, Michael Woods, Evelyn Grau, and Brendan Doe from the research support facility (RSF) at the Sanger Institute for providing blastocysts. We also thank Prof. Austin Smith and Dr. Ge Guo from Wellcome Trust-Medical Research Council Cambridge Stem Cell Institute for the reagents. This work was funded by the Wellcome Trust (WT098051). J.Y. is funded by the National Science Foundation of China (NSFC) (31871491). E.M. is funded by the UK Dementia Research Institute (RG86445) and Parkinson's Disease UK (F-1501).

Received: August 28, 2018
Revised: February 17, 2019
Accepted: February 20, 2019
Published: March 21, 2019

## REFERENCES

Adachi, K., Nikaido, I., Ohta, H., Ohtsuka, S., Ura, H., Kadota, M., Wakayama, T., Ueda, H.R., and Niwa, H. (2013). Context-dependent wiring of Sox2 regulatory networks for self-renewal of embryonic and trophoblast stem cells. Mol. Cell *52*, 380–392.

Awe, J.P., and Byrne, J.A. (2013). Identifying candidate oocyte reprogramming factors using cross-species global transcriptional analysis. Cell Reprogram. *15*, 126–133.

Azami, T., Matsumoto, K., Jeon, H., Waku, T., Muratani, M., Niwa, H., Takahashi, S., and Ema, M. (2018). Klf5 suppresses ERK signaling in mouse pluripotent stem cells. PLoS One *13*, e0207321.

Bagci, H., and Fisher, A.G. (2013). DNA demethylation in pluripotency and reprogramming: the role of tet proteins and cell division. Cell Stem Cell *13*, 265–269.

Barau, J., Teissandier, A., Zamudio, N., Roy, S., Nalesso, V., Herault, Y., Guillou, F., and Bourc'his, D. (2016). The DNA methyltransferase DNMT3C protects male germ cells from transposon activity. Science *354*, 909–912.

Basta, J.M., Robbins, L., Denner, D.R., Kolar, G.R., and Rauchman, M. (2017). A Sall1-NuRD interaction regulates multipotent nephron progenitors and is required for loop of Henle formation. Development *144*, 3080–3094.

Bester, A.C., Lee, J.D., Chavez, A., Lee, Y.R., Nachmani, D., Vora, S., Victor, J., Sauvageau, M., Monteleone, E., Rinn, J.L., et al. (2018). An integrated genome-wide CRISPRa approach to functionalize lncRNAs in drug resistance. Cell *173*, 649–664.e20.

Biswas, D., and Jiang, P. (2016). Chemically induced reprogramming of somatic cells to pluripotent stem cells and neural cells. Int. J. Mol. Sci. *17*, 226.

Castro, D.S., Martynoga, B., Parras, C., Ramesh, V., Pacary, E., Johnston, C., Drechsel, D., Lebel-Potter, M., Garcia, L.G., Hunt, C., et al. (2011). A novel function of the proneural factor Ascl1 in progenitor proliferation identified by genome-wide characterization of its targets. Genes Dev. *25*, 930–945.

Chappell, J., and Dalton, S. (2013). Roles for MYC in the establishment and maintenance of pluripotency. Cold Spring Harb. Perspect. Med. *3*, a014381.

Chavez, A., Tuttle, M., Pruitt, B.W., Ewen-Campen, B., Chari, R., Ter-Ovanesyan, D., Haque, S.J., Cecchi, R.J., Kowal, E.J.K., Buchthal, J., et al. (2016). Comparison of Cas9 activators in multiple species. Nat. Methods *13*, 563–567.

Ding, J., Huang, X., Shao, N., Zhou, H., Lee, D.F., Faiola, F., Fidalgo, M., Guallar, D., Saunders, A., Shliaha, P.V., et al. (2015). Tex10 coordinates epigenetic control of super-enhancer activity in pluripotency and reprogramming. Cell Stem Cell *16*, 653–668.

Doudna, J.A., and Charpentier, E. (2014). Genome editing. The new frontier of genome engineering with CRISPR-Cas9. Science *346*, 1258096.

Ema, M., Mori, D., Niwa, H., Hasegawa, Y., Yamanaka, Y., Hitoshi, S., Mimura, J., Kawabe, Y., Hosoya, T., Morita, M., et al. (2008). Kruppel-like factor 5 is essential for blastocyst development and the normal self-renewal of mouse ESCs. Cell Stem Cell *3*, 555–567.

Festuccia, N., Owens, N., and Navarro, P. (2017). Esrrb, an estrogen-related receptor involved in early development, pluripotency, and reprogramming. FEBS Lett. https://doi.org/10.1002/1873-3468.12826.

Fidalgo, M., Huang, X., Guallar, D., Sanchez-Priego, C., Valdes, V.J., Saunders, A., Ding, J., Wu, W.S., Clavel, C., and Wang, J. (2016). Zfp281 coordinates opposing functions of Tet1 and Tet2 in pluripotent states. Cell Stem Cell *19*, 355–369.

Gaj, T., Gersbach, C.A., and Barbas, C.F., 3rd. (2013). ZFN, TALEN, and CRISPR/Cas-based methods for genome engineering. Trends Biotechnol. *31*, 397–405.

Gao, X., Yang, J., Tsang, J.C., Ooi, J., Wu, D., and Liu, P. (2013). Reprogramming to pluripotency using designer TALE transcription factors targeting enhancers. Stem Cell Reports *1*, 183–197.

Gaspar-Maia, A., Qadeer, Z.A., Hasson, D., Ratnakumar, K., Leu, N.A., Leroy, G., Liu, S., Costanzi, C., Valle-Garcia, D., Schaniel, C., et al. (2013). MacroH2A histone variants act as a barrier upon reprogramming towards pluripotency. Nat. Commun. *4*, 1565.

Gayle, S., Pan, Y., Landrette, S., and Xu, T. (2015). piggyBac insertional mutagenesis screen identifies a role for nuclear RHOA in human ES cell differentiation. Stem Cell Reports *4*, 926–938.

Guo, G., and Smith, A. (2010). A genome-wide screen in EpiSCs identifies Nr5a nuclear receptors as potent inducers of ground state pluripotency. Development *137*, 3185–3192.

Guo, G., Yang, J., Nichols, J., Hall, J.S., Eyres, I., Mansfield, W., and Smith, A. (2009). Klf4 reverts developmentally programmed restriction of ground state pluripotency. Development *136*, 1063–1069.

Guo, J., Ma, D., Huang, R., Ming, J., Ye, M., Kee, K., Xie, Z., and Na, J. (2017). An inducible CRISPR-ON system for controllable gene activation in human pluripotent stem cells. Protein Cell *8*, 379–393.

Hall, J., Guo, G., Wray, J., Eyres, I., Nichols, J., Grotewold, L., Morfopoulou, S., Humphreys, P., Mansfield, W., Walker, R., et al. (2009). Oct4 and LIF/Stat3 additively induce Kruppel factors to sustain embryonic stem cell self-renewal. Cell Stem Cell *5*, 597–609.

Heaton, B.E., Kennedy, E.M., Dumm, R.E., Harding, A.T., Sacco, M.T., Sachs, D., and Heaton, N.S. (2017). A CRISPR activation screen identifies a pan-avian influenza virus inhibitory host factor. Cell Rep. *20*, 1503–1512.

Hou, P., Li, Y., Zhang, X., Liu, C., Guan, J., Li, H., Zhao, T., Ye, J., Yang, W., Liu, K., et al. (2013). Pluripotent stem cells induced from mouse somatic cells by small-molecule compounds. Science *341*, 651–654.

Hu, G., Kim, J., Xu, Q., Leng, Y., Orkin, S.H., and Elledge, S.J. (2009). A genome-wide RNAi screen identifies a new transcriptional module required for self-renewal. Genes Dev. *23*, 837–848.

Huangfu, D., Osafune, K., Maehr, R., Guo, W., Eijkelenboom, A., Chen, S., Muhlestein, W., and Melton, D.A. (2008). Induction of pluripotent stem cells from primary human fibroblasts with only Oct4 and Sox2. Nat. Biotechnol. *26*, 1269–1275.

Iseki, H., Nakachi, Y., Hishida, T., Yamashita-Sugahara, Y., Hirasaki, M., Ueda, A., Tanimoto, Y., Iijima, S., Sugiyama, F., Yagami, K., et al. (2016). Combined overexpression of JARID2, PRDM14, ESRRB, and SALL4A dramatically improves efficiency and kinetics of reprogramming to induced pluripotent stem cells. Stem Cells *34*, 322–333.

Jeon, H., Waku, T., Azami, T., Khoa le, T.P., Yanagisawa, J., Takahashi, S., and Ema, M. (2016). Comprehensive identification of Kruppel-like factor family members contributing to the self-renewal of mouse embryonic stem cells and cellular reprogramming. PLoS One *11*, e0150715.

Jinek, M., Chylinski, K., Fonfara, I., Hauer, M., Doudna, J.A., and Charpentier, E. (2012). A programmable dual-RNA-guided DNA endonuclease in adaptive bacterial immunity. Science *337*, 816–821.

Kampmann, M. (2018). CRISPRi and CRISPRa screens in mammalian cells for precision biology and medicine. ACS Chem. Biol. *13*, 406–416.

Karantzali, E., Lekakis, V., Ioannou, M., Hadjimichael, C., Papamatheakis, J., and Kretsovali, A. (2011). Sall1 regulates embryonic stem cell differentiation in association with nanog. J. Biol. Chem. *286*, 1037–1045.

Karwacki-Neisius, V., Goke, J., Osorno, R., Halbritter, F., Ng, J.H., Weisse, A.Y., Wong, F.C., Gagliardi, A., Mullin, N.P., Festuccia, N., et al. (2013). Reduced Oct4 expression directs a robust pluripotent state with distinct signaling activity and increased enhancer occupancy by Oct4 and Nanog. Cell Stem Cell *12*, 531–545.

Koike-Yusa, H., Li, Y., Tan, E.P., Velasco-Herrera Mdel, C., and Yusa, K. (2014). Genome-wide recessive genetic screening in mammalian cells with a lentiviral CRISPR-guide RNA library. Nat. Biotechnol. *32*, 267–273.

Konermann, S., Brigham, M.D., Trevino, A.E., Joung, J., Abudayyeh, O.O., Barcena, C., Hsu, P.D., Habib, N., Gootenberg, J.S., Nishimasu, H., et al. (2015). Genome-scale transcriptional activation by an engineered CRISPR-Cas9 complex. Nature *517*, 583–588.

Lessing, D., Anguera, M.C., and Lee, J.T. (2013). X chromosome inactivation and epigenetic responses to cellular reprogramming. Annu. Rev. Genomics Hum. Genet. *14*, 85–110.

Li, Q., Lex, R.K., Chung, H., Giovanetti, S.M., Ji, Z., Ji, H., Person, M.D., Kim, J., and Vokes, S.A. (2016). The pluripotency factor NANOG binds to GLI proteins and represses hedgehog-mediated transcription. J. Biol. Chem. *291*, 7171–7182.

Liu, P., Chen, M., Liu, Y., Qi, L.S., and Ding, S. (2018a). CRISPR-based chromatin remodeling of the endogenous Oct4 or Sox2 locus enables reprogramming to pluripotency. Cell Stem Cell *22*, 252–261.e4.

Liu, Y., Yu, C., Daley, T.P., Wang, F., Cao, W.S., Bhate, S., Lin, X., Still, C., 2nd, Liu, H., Zhao, D., et al. (2018b). CRISPR activation screens systematically identify factors that drive neuronal fate and reprogramming. Cell Stem Cell *23*, 758–771.e8.

Mansour, A.A., Gafni, O., Weinberger, L., Zviran, A., Ayyash, M., Rais, Y., Krupalnik, V., Zerbib, M., Amann-Zalcenstein, D., Maza, I., et al. (2012). The H3K27 demethylase Utx regulates somatic and germ cell epigenetic reprogramming. Nature *488*, 409–413.

Martello, G., and Smith, A. (2014). The nature of embryonic stem cells. Annu. Rev. Cell Dev. Biol. *30*, 647–675.

Metzakopian, E., Strong, A., Iyer, V., Hodgkins, A., Tzelepis, K., Antunes, L., Friedrich, M.J., Kang, Q., Davidson, T., Lamberth, J., et al. (2017). Enhancing the genome editing toolbox: genome wide CRISPR arrayed libraries. Sci. Rep. *7*, 2244.

Mihaylova, M.M., and Shaw, R.J. (2013). Metabolic reprogramming by class I and II histone deacetylases. Trends Endocrinol. Metab. *24*, 48–57.

Mitsui, K., Tokuzawa, Y., Itoh, H., Segawa, K., Murakami, M., Takahashi, K., Maruyama, M., Maeda, M., and Yamanaka, S. (2003). The homeoprotein Nanog is required for maintenance of pluripotency in mouse epiblast and ES cells. Cell *113*, 631–642.

Nichols, J., and Smith, A. (2009). Naive and primed pluripotent states. Cell Stem Cell *4*, 487–492.

Niwa, H., Burdon, T., Chambers, I., and Smith, A. (1998). Self-renewal of pluripotent embryonic stem cells is mediated via activation of STAT3. Genes Dev. *12*, 2048–2060.

Niwa, H., Miyazaki, J., and Smith, A.G. (2000). Quantitative expression of Oct-3/4 defines differentiation, dedifferentiation or self-renewal of ES cells. Nat. Genet. *24*, 372–376.

Novo, C.L., Tang, C., Ahmed, K., Djuric, U., Fussner, E., Mullin, N.P., Morgan, N.P., Hayre, J., Sienerth, A.R., Elderkin, S., et al. (2016). The pluripotency factor Nanog regulates pericentromeric heterochromatin organization in mouse embryonic stem cells. Genes Dev. *30*, 1101–1115.

Pritsker, M., Ford, N.R., Jenq, H.T., and Lemischka, I.R. (2006). Genomewide gain-of-function genetic screen identifies functionally active genes in mouse embryonic stem cells. Proc. Natl. Acad. Sci. U S A *103*, 6946–6951.

Qiu, D., Ye, S., Ruiz, B., Zhou, X., Liu, D., Zhang, Q., and Ying, Q.L. (2015). Klf2 and Tfcp2l1, two Wnt/beta-catenin targets, act synergistically to induce and maintain naive pluripotency. Stem Cell Reports *5*, 314–322.

Radzisheuskaya, A., Chia Gle, B., dos Santos, R.L., Theunissen, T.W., Castro, L.F., Nichols, J., and Silva, J.C. (2013). A defined Oct4 level governs cell state transitions of pluripotency entry and differentiation into all embryonic lineages. Nat. Cell Biol. *15*, 579–590.

Sandmaier, S.E., and Telugu, B.P. (2015). MicroRNA-mediated reprogramming of somatic cells into induced pluripotent stem cells. Methods Mol. Biol. *1330*, 29–36.

Shalem, O., Sanjana, N.E., Hartenian, E., Shi, X., Scott, D.A., Mikkelson, T., Heckl, D., Ebert, B.L., Root, D.E., Doench, J.G., and Zhang, F. (2014). Genome-scale CRISPR-Cas9 knockout screening in human cells. Science *343*, 84–87.

Shu, J., Wu, C., Wu, Y., Li, Z., Shao, S., Zhao, W., Tang, X., Yang, H., Shen, L., Zuo, X., et al. (2013). Induction of pluripotency in mouse somatic cells with lineage specifiers. Cell *153*, 963–975.

Silva, J., Barrandon, O., Nichols, J., Kawaguchi, J., Theunissen, T.W., and Smith, A. (2008). Promotion of reprogramming to ground state pluripotency by signal inhibition. PLoS Biol. *6*, e253.

Silva, J., Nichols, J., Theunissen, T.W., Guo, G., van Oosten, A.L., Barrandon, O., Wray, J., Yamanaka, S., Chambers, I., and Smith, A. (2009). Nanog is the gateway to the pluripotent ground state. Cell *138*, 722–737.

Singhal, N., Graumann, J., Wu, G., Arauzo-Bravo, M.J., Han, D.W., Greber, B., Gentile, L., Mann, M., and Scholer, H.R. (2010). Chromatin-remodeling components of the BAF complex facilitate reprogramming. Cell *141*, 943–955.

Smith, A. (2017). Formative pluripotency: the executive phase in a developmental continuum. Development *144*, 365–373.

Takahashi, K., and Yamanaka, S. (2006). Induction of pluripotent stem cells from mouse embryonic and adult fibroblast cultures by defined factors. Cell *126*, 663–676.

Tanenbaum, M.E., Gilbert, L.A., Qi, L.S., Weissman, J.S., and Vale, R.D. (2014). A protein-tagging system for signal amplification in gene expression and fluorescence imaging. Cell *159*, 635–646.

Tang, Y., and Tian, X.C. (2013). JAK-STAT3 and somatic cell reprogramming. JAKSTAT *2*, e24935.

Tesar, P.J., Chenoweth, J.G., Brook, F.A., Davies, T.J., Evans, E.P., Mack, D.L., Gardner, R.L., and McKay, R.D. (2007). New cell lines from mouse epiblast share defining features with human embryonic stem cells. Nature *448*, 196–199.

Theunissen, T.W., van Oosten, A.L., Castelo-Branco, G., Hall, J., Smith, A., and Silva, J.C. (2011). Nanog overcomes reprogramming barriers and induces pluripotency in minimal conditions. Curr. Biol. *21*, 65–71.

Wang, W., Yang, J., Liu, H., Lu, D., Chen, X., Zenonos, Z., Campos, L.S., Rad, R., Guo, G., Zhang, S., et al. (2011). Rapid and efficient reprogramming of somatic cells to induced pluripotent stem cells by retinoic acid receptor gamma and liver receptor homolog 1. Proc. Natl. Acad. Sci. U S A *108*, 18283–18288.

Warren, L., Manos, P.D., Ahfeldt, T., Loh, Y.H., Li, H., Lau, F., Ebina, W., Mandal, P.K., Smith, Z.D., Meissner, A., et al. (2010). Highly efficient reprogramming to pluripotency and directed differentiation of human cells with synthetic modified mRNA. Cell Stem Cell *7*, 618–630.

Weltner, J., Balboa, D., Katayama, S., Bespalov, M., Krjutskov, K., Jouhilahti, E.M., Trokovic, R., Kere, J., and Otonkoski, T. (2018).

Human pluripotent reprogramming with CRISPR activators. Nat. Commun. *9*, 2643.

Yang, J., van Oosten, A.L., Theunissen, T.W., Guo, G., Silva, J.C., and Smith, A. (2010). Stat3 activation is limiting for reprogramming to ground state pluripotency. Cell Stem Cell. *7*, 319–328.

Ying, Q.L., Stavridis, M., Griffiths, D., Li, M., and Smith, A. (2003). Conversion of embryonic stem cells into neuroectodermal precursors in adherent monoculture. Nat. Biotechnol. *21*, 183–186.

Yu, Y., Gu, S., Li, W., Sun, C., Chen, F., Xiao, M., Wang, L., Xu, D., Li, Y., Ding, C., et al. (2017). Smad7 enables STAT3 activation and promotes pluripotency independent of TGF-beta signaling. Proc. Natl. Acad. Sci. U S A *114*, 10113–10118.

Yusa, K., Zhou, L., Li, M.A., Bradley, A., and Craig, N.L. (2011). A hyperactive piggyBac transposase for mammalian applications. Proc. Natl. Acad. Sci. U S A *108*, 1531–1536.

Zhang, J., Tam, W.L., Tong, G.Q., Wu, Q., Chan, H.Y., Soh, B.S., Lou, Y., Yang, J., Ma, Y., Chai, L., et al. (2006). Sall4 modulates embryonic stem cell pluripotency and early embryonic development by the transcriptional regulation of Pou5f1. Nat. Cell Biol. *8*, 1114–1123.

Zhao, Y., Yin, X., Qin, H., Zhu, F., Liu, H., Yang, W., Zhang, Q., Xiang, C., Hou, P., Song, Z., et al. (2008). Two supporting factors greatly improve the efficiency of human iPSC generation. Cell Stem Cell *3*, 475–479.

**Stem Cell Reports, Volume *12***

## Supplemental Information

# Genome-Scale CRISPRa Screen Identifies Novel Factors for Cellular Reprogramming

**Jian Yang, Sandeep S. Rajan, Mathias J. Friedrich, Guocheng Lan, Xiangang Zou, Hannes Ponstingl, Dimitrios A. Garyfallos, Pentao Liu, Allan Bradley, and Emmanouil Metzakopian**

# Genome-scale CRISPRa screen identifies novel factors for cellular reprogramming

**Jian Yang[‡⊥+], Sandeep S Rajan[‡†+], Mathias J Friedrich[‡+], Guocheng Lan[¶], Xiangang Zou[¶], Hannes Ponstingl[‡], Dimitrios A Garyfallos[‡], Pentao Liu[‡¢], Allan Bradley[‡], and Emmanouil Metzakopian[‡†*]**

[‡]Wellcome Trust Sanger Institute, Genome Campus, Hinxton, Cambridgeshire, CB10 1SA, UK

[†]UK Dementia Research Institute, Department of Clinical Neuroscience, University of Cambridge, CB2 0AH, UK

[¶]Cancer Research UK, Cambridge Institute, Li Ka Shing Centre, University of Cambridge, Cambridge, CB2 0RE, UK

[¢]School of Biomedical Sciences, Li Ka Shing Faculty of Medicine, Stem Cell and Regenerative Medicine Consortium, University of Hong Kong, Hong Kong, China

[⊥]*Current address:* Key Laboratory of Arrhythmias, Ministry of Education, Shanghai East Hospital, Tongji University School of Medicine, Shanghai 200120, China

[+]These authors contributed equally to the work.

*Correspondence should be addressed to:

**Emmanouil Metzakopian**

UK Dementia Research Institute
Cambridge Biomedical Campus
University of Cambridge
Cambridge, CB2 0AH, U.K

Email: em698@medschl.cam.ac.uk

# Supplementary Document

## Experimental Procedures

### Ethics Statement

All animal experimental procedures were carried out in accordance with Home Office UK regulations and the Animals (Scientific Procedures) Act 1986 (licence No. 70/8387 and 80/2552). All experimental protocols were approved by the Animal Welfare and Ethical Review Body (AWERB) of Wellcome Genome Campus and the University of Cambridge CRUK Cambridge Institute. At the end of the study, mice were euthanized in accordance with stated Home Office UK regulations.

### Constructs

Guide RNAs (sgRNAs) were expressed under the U6 promoter in expression constructs (pKLV-PB-U6-gRNA-PGK-Puro-T2A-TagBFP, Figure S1) harbouring *PiggyBac inverted terminal repeats to enable transposase-mediated genomic integration* (*PB* transposon) and HIV-1 long terminal repeats to allow lentiviral genomic integration (Figure S1). The sgRNA scaffold used in conjunction with dCas9:SAM was adapted to contain two MS2-binding loops as required by the SAM CRISPRa system (Konermann et al. 2015). The constructs also included a puromycin antibiotic resistance and a TagBFP marker. The four dCas9 variants were cloned into PB transposons and included the mCherry fluorescent marker and Blasticidine antibiotic resistance. The dCas9 cDNAs in these constructs were driven by the Ef1-α promoter and multiple consecutive cDNAs were linked by the T2A self-separating peptide sequence.

cDNAs of *Oct4*, *c-Myc*, *Klf4*, *Sox2*, *Sall1* and *Nanog* were cloned into *PiggyBac* transposons under the control of the CAG promoter (PBCAG) or the Tet response element (PBTRE). Combinations of cDNAs were linked by the T2A self-separating peptide sequence. PBEF1α-TET3G encoding the Tet-On-3G transactivator protein was co-transfected with PBTRE-cDNA to enable Doxycycline induction of the PBTRE constructs.

When stable integration by transposition of the transgene was required, a plasmid encoding *PiggyBac* transposase (*HyPBase* (Yusa et al. 2011)) was co-transfected. Schematic representations of all the constructs used in the study are shown in Figure S1.

**Cell Culture**

*Oct4*-GFP and *Nanog*-GFP Epiblast stem cells (EpiSCs) were generated as described previously (Yang et al. 2010). Briefly, EpiSC were derived from post-implanted pregnant transgenic mice at E5.75. Cells from embryos were cultured on human fibronectin (Millipore) coated plates in complete EpiSC media based on N2B27 which comprised 50% Neurobasal media, 50% DMEM-F12 media, 0.1mM β-ME, penicillin (100 U/ml), streptomycin (100 µg/ml), L-glutamine (2 mM), 1X N2 and 1X B27 supplement (Invitrogen) and was supplemented with 20 ng/ml Activin-A (R & D Systems) and 12 ng/ml FGF2 (Peprotech). When confluent, the media was aspirated and the EpiSC were dissociated with Accutase (Millipore) for 3 min. Dissociated cells were spun down at 300g for 5 min and plated either at 1:6 or at 1:8 split ratio on human fibronectin coated plates in complete EpiSC media for maintenance.

For screening and reprogramming, EpiSCs were cultured in 2i/LIF medium comprising N2B27 media (as above) supplemented with 100 U/ml leukemia inhibitory factor (LIF, Millipore), 1 μM PD0325901 (Tocris) and 3 μM CHIR99021 (Tocris).

*Rex1*-GFP embryonic stem cells (ESCs) cells were generated as described previously (Wang et al. 2011). ESCs were cultured on 0.1% gelatin coated plates. ES cells were regularly maintained in ESC medium (M15) comprising knock-out DMEM containing 15% FBS, 0.1mM β-ME, penicillin (100 U/ml), streptomycin (100 μg/ml), L-glutamine (2 mM), and 100 U/ml LIF. Confluent ES cells were dissociated with 0.025% Trypsin-EDTA for 5 min. Detached cells were collected and spun down at 300g for 5 min and were plated at a split ratio of either 1:8 or at 1:10 on gelatin coated plates.

Mouse embryonic fibroblasts (MEFs) were cultured in M10 medium comprising knock-out DMEM containing 10% FBS, 0.1mM β-ME, penicillin (100 U/ml), streptomycin (100 μg/ml) and L-glutamine (2 mM). For reprogramming, after transfection, cells were cultured in ESC medium until the end of experiment or until the colonies were picked for iPSC line derivation.

**GoF gRNA library design**

The Gain of Function (GoF) library targeted the region of up to 250 base pairs upstream of the transcription start site (TSS) of each protein-coding gene. Up to 4 guides of 19 bp length were selected per gene. Protein coding genes and TSSs were obtained from the mouse reference assembly GRCm38 in the ENSEMBL database (version 78, http://www.ensembl.org) and TSSs were checked against CAGE data in the FANTOM data base (April 2015, http://fantom.gsc.riken.jp/).

Potential guide sequences were identified on the reference assembly as the 19 bp sequence at the 5' end of each PAM motif (NGG), i.e. 2 to 20 bp upstream of each

guanosine di-nucleotide in the reference. Guide sequences consisted of no more than 13 guanosine or cytosine bases (GC content < 70%). Guides in the 250 bp region on both DNA strands upstream of the TSS were then compared to all other potential guide sequences across the genome. Guide sequences with off-target sites exhibiting fewer than 3 mismatches over their 19 bp length were omitted from the design.

The remaining guides were sorted by a simple ad-hoc quality score intended to reflect a likely increased tolerance of mismatches distal of the PAM motif. For each potential off-target site a score was calculated that linearly increased with the number of mismatches and decreased with their distance to the PAM motif.

A selection algorithm was designed to spread high quality guides across the target region. To this end, the region upstream of the TSS was divided in quarters of roughly equal length. Starting with the quarter closest to the TSS the algorithm looped over quarters picking the best guide, by quality score, in each if available and adding it to the library until no more guide fitting the constraints could be found, or the target number of 5 guides per genes was reached. A constraint for the GC content of less than 55% was applied in the first loop and then relaxed to 70%.

**GoF reprogramming screen**

The GoF sgRNA library was synthesized by Custom Array, and the oligo pools were cloned into the lentiviral sgRNA expression plasmid via Gibson assembly as described by Shalem *et al*.(Shalem et al. 2014), with minor modifications.

*Oct4*-GFP EpiSC cells were transfected with 200 ng of plasmid encoding *PiggyBac* transposase together with 1 µg dCas9:SAM to facilitate stable integration. Transfections were performed with Lipofectamine LTX (Invitrogen), according to manufacturer's instructions. Transfected cells were then selected by 10 µg/ml

Blasticidine (Gibco) for 10 days. Post-selection, dCas9:SAM expressing *Oct4*-GFP EpiSC were expanded to 100 x $10^6$ cells for lentiviral transduction.

Library transduction was carried out at a MOI (multiplicity of infection) of 0.3. After two days, 10 x $10^6$ BFP$^{+ve}$ *Oct4*-GFP EpiSCs were sorted by flow cytometry. The sorted cells were seeded on fibronectin coated plates and allowed to recover in complete EpiSC medium for 24 h. The medium was then changed to 2i/LIF in order to allow selection for reprogrammed cells. After 14-16 days in 2i/LIF, the individual reprogrammed colonies, verified by *Oct4*-GFP fluorescence, were picked and transferred to 96 well plates. Colonies were passaged twice in 2i/LIF before sequencing. Genomic DNA was extracted from each colony and PCR amplification across the stably integrated sgRNA was performed using primers described previously (Koike-Yusa et al. 2014). PCR amplicon libraries were pooled and Next Generation Sequencing was used to identify the distribution of sgRNA sequences.

**CRISPRa transfections**

*Oct4*-GFP EpiSC and *Nanog*-GFP EpiSC cells were grown to 70% confluence on fibronectin coated 6-well plates. The cells were dissociated with Accutase and re-suspended in EpiSC media for reverse transfections (approx. 1 X $10^6$ cells in 250 µl per transfection).

Cells were transfected with 500 ng *PiggyBac* transposase together with 500 ng dCas9:SAM and 500 ng of sgRNA expression construct for one or more sgRNAs. Transfections were performed, in triplicate, using Lipofectamine LTX (Invitrogen), according to manufacturer's instructions. Cells were cultured in EpiSC medium for 24 h. Stably-transfected cell lines were generated by selection with Blasticidine

(10 µg/ml) for at least 10 days post-transfection. Integration of constructs was confirmed by PCR genotyping (Table S1).

**cDNA transfections**

*Oct4*-GFP EpiSCs, *Nanog*-GFP EpiSCs and *Rex1*-GFP ESCs were transfected, in triplicate, with 1 µg PBCAG expressing either *Sall1*, *Nanog* or a combination of both vectors via Lipofectamine LTX. Cells were co-transfected with plasmids encoding *PiggyBac* transposase (500 ng) and mCherry Blasticidine (100 ng) selection markers to enable selection of transfected cells. Presence of PBCAG in the selected cells was confirmed with PCR (Table S1).

**EpiSC Reprogramming**

Stable lines of *Oct4*-GFP and *Nanog*-GFP EpiSC generated either from CRISPRa or cDNA transfections were plated in triplicate on fibronectin coated 6-well plates in EpiSC medium. Medium was changed to 2i/LIF when cells reached 80% confluence and thereafter replaced every 2 days to select for reprogramming for up to 20 and 24 days for *Oct4*-GFP transfected EpiSCs and *Nanog*-GFP transfected EpiSCs, respectively. GFP$^{+ve}$ ESC-like colonies were counted and transferred onto gelatin coated plates for expansion. Expanded colonies were then phenotyped for gene expression by RT-qPCR (Table S3).

To derive iPSCs for blastocyst injection, *Oct4*-GFP EpiSC were transfected with 1 µg PBTRESall1, 1 µg PBTET3G and 2 µg transposase using Lipofectamine LTX. Transgene expression was induced by supplementing the medium with 0.5 µg/ml Doxycycline after switching the cells to 2i/LIF.

**Flow cytometry analysis of EpiSC reprogramming**

Stably transfected CRISPRa *Oct4*-GFP and CRISPRa *Nanog*-GFP EpiSC were plated in triplicates on fibronectin coated 24-well plates at a density of 50,000 cells per well, in EpiSC media. Upon reaching confluence, the media was changed to 2i/LIF to select for reprogramming. Cells were harvested at regular time intervals and were analysed for GFP fluorescence in a flow cytometer (CytoFLEX, Beckman Coulter Life Sciences, Indianapolis US).

**MEF Reprogramming**

For sgRNA mediated reprogramming of MEFs, $1 \times 10^6$ *Oct4*-GFP MEFs were electroporated with 0.5 µg PBCAG4F, 1 µg gRNA Sall1 / 1 µg gRNA Nanog / 0.5 µg gRNA Sall1+0.5 µg gRNA Nanog, 1 µg dCas9:SAM and 0.5 µg *PiggyBac* transposase using the Amaxa Nucleofector (Amaxa, Lonza). The transfected cells were plated onto gelatin-coated 10 cm dishes in M15. All transfections were performed in triplicates. After 24 h, the medium was replaced and was renewed every two days until day 18 when *Oct4*-GFP$^{+ve}$ and AP$^{+ve}$ cells were counted.

For inducible cDNA mediated reprogramming, $1 \times 10^6$ *Oct4*-GFP MEFs were electroporated with 1 µg PBTRE4F, 0.5 µg PBTRESall1, 2 µg PBEF-1αTet3G and 2 µg *PiggyBac* transposase using the Amaxa Nucleofector (Amaxa, Lonza). The transfected cells were plated in gelatin-coated 10 cm dishes in ESC medium. All transfections were performed in triplicates. After 24 h, the medium was replaced and three different concentrations of Doxycycline (0.1, 0.5 and 1.0 µg/ml) were tested for induction of cDNA expression. At day 12, Doxycycline was withdrawn and the cells were cultured for 6 more days, after which *Oct4*-GFP$^{+ve}$ and alkaline phosphatase stained colonies were counted and single colonies picked for RT-qPCR.

To produce iPSCs for *in vitro* and *in vivo* assays, C57B/6J MEFs were transfected with the same amount of PBTRE4F, PBTRESall1, PBEF1α-TET3G and *PiggyBac* transposase as described above and induced with 0.5 µg/ml Doxycycline in ESC media.

All the combinations of cDNA transfections for MEF reprogramming are listed in Table S2.

**Western Blotting**

*Oct4*-GFP EpiSCs transfected with CRISPRa *Oct4* and cDNA *Oct4* (both CAG and TRE) together with experimental controls and untransfected cells (EpiSCs and ESCs) were collected for Western blotting after 3 days 2i/LIF (Dox induction for 3 days). Protein amounts were determined using a Bradford assay and 30 µg of lysates were subjected to electrophoresis on 4–15% Mini-PROTEAN® TGX™ Precast Protein Gels (Biorad). Proteins were transferred onto PVDF membranes (Millipore) overnight at 30 V for 8 hours at 4°C. Transferred proteins were then immunoblotted for Oct4 (c-10, Santa Cruz, #sc-5279, dilution 1:800) and Gapdh (Sigma, #G8795, dilution 1:4000). All antibodies used are listed in Table S4.

**Conversion of ESCs to EpiSCs**

Stable lines of cDNA transfected *Rex1*-GFP ESCs were cultured in 2i/LIF medium for 3 days prior to the conversion. Upon reaching 50% confluence, cells were dissociated and seeded on gelatin-coated plates in EpiSC medium for conversion for at least 3 passages. Cells were seeded on fibronectin-coated plates after the 2$^{nd}$ passage to promote differentiation. In addition, 600 cells were plated back on 0.1% gelatin coated plates in 2i/LIF medium for about 7 days to promote formation of iPS colonies. The colonies were assessed by AP staining. Lastly, at every passage, cells were assessed

for *Rex1*-GFP fluorescence using flow cytometry and total RNA was extracted for RT-qPCR. All ESC to EpiSC conversion experiments were carried out in triplicates.

**Quantitative RT-PCR (RT-qPCR)**

Total RNA was extracted using the RNeasy Mini Kit (Qiagen) according to manufacturer's instructions. First strand cDNA was synthesized using qScript cDNA Supermix (Quantabio) according to manufacturer's protocol. All qPCR studies were performed using Taqman Gene Expression Assays either in the 7900HT Fast Real-Time PCR system (Applied Biosystems) or the StepOnePlus™ Real-Time PCR System (Applied Biosystems). Samples were run in triplicate, from 3 independent experiments, for both gene of interest and house-keeping genes. Expression levels were normalized to *Gapdh*. TaqMan probes used are listed in Table S3.

**RNA-sequencing**

*Oct4*-GFP EpiSCs were transfected via Lipofectamine LTX with 1 µg PBCAGSall1, PBCAGNanog, PBCAGSall1+PBCAGNanog or empty vector (PBCAG:Empty). Cells were co-transfected with CAGmCherry (100 ng) to enable fluorescent marker selection. All transfections were performed in triplicates. 24 h later, cells were FACS sorted for GFP$^{+ve}$mCherry$^{+ve}$ and their RNA was extracted using the Rneasy Mini Kit (Qiagen) according to manufacturer's instructions. Illumina Truseq NGS libraries were prepared and sequenced on a Illumina HiSeq instrument.

Sequencing results were analysed by aligning reads to the mouse genome build GRCm38.p5 using the STAR aligner (Ver. 2.5.3a) and read counts were prepared with the TOPHAT package. Differential expression of genes was analysed with the DESeq2 package for the R statistical computing framework. We used a cut-off padj value of <0.001 to determine genes that were differentially regulated between

experimental and control samples. All data from RNA-seq is available from ArrayExpress (https://www.ebi.ac.uk/arrayexpress/) using accession number: E-MTAB-7692.

**GO Term analysis**

We used the GoToolBox platform (http://genome.crg.es/GOToolBox/) to perform enrichment analysis on the genes identified in the CRISPRa screen and via RNAseq as described previously (Castro et al. 2011). In brief, a Data-Set was created using the Mouse Genome Informatics version (MGI) and then used for pathway enrichment analysis. Fold changes for enriched gene sets were computed by dividing the frequency in our set by that of the reference. Representative pathways with a fold change of 2 or more and with a p-value of 0.05 or lower were graphically represented in the results.

**Immunofluorescence**

C57BL/6J MEF reprogrammed iPSCs were plated at 2 x $10^3$ onto 24 well plates in 2i/LIF. After 24 hours, cells were washed with PBS and fixed in 4% paraformaldehyde (PFA), blocked and permeabilised with 1% bovine serum albumin (BSA) and 3% serum in PBS with 0.1%Triton X100. Samples were incubated with mouse anti-SSEA-1 (BD Biosciences) or rabbit anti-*Nanog* (Abcam) antibodies at 4℃ overnight, then rinsed and incubated with Alexa488-conjugated goat anti-Mouse IgM and Alexa594-conjugated goat anti-Rabbit IgG (Invitrogen), and counterstained with DAPI.

To examine the X chromosome status in female iPSCs, cells were plated at 5 x $10^3$ on gelatin coated slides in 2i/LIF or in M10 medium for 5 days, then cells were fixed in 4% PFA, and immunofluorescence was performed as mentioned above. Slides were incubated with rabbit anti-H3K27me3 (Millipore) and mouse anti-*Oct4* antibodies

(Santa Cruz), then Alexa594-conjugated goat anti-Rabbit IgG and Alexa488 goat anti-Mouse IgG and counterstained with DAPI. All antibodies used are listed in Table S4.

***In Vitro* differentiation**

C57BL/6J MEF reprogrammed iPSCs were plated at 5 x $10^5$ in a petri-dish in M10 medium for 4 days, then dissociated with 0.05% Trypsin/EDTA and plated at 1 x $10^5$ in M10 on gelatinized plates for another 4 days. The cells were then fixed in 4% PFA and examined for mesoderm and endoderm markers using immunofluorescent staining with antibodies against smooth muscle antigen (SMA) (R&D Systems) and alpha fetoprotein (AFP) (R&D Systems). For neuronal differentiation, cells were plated at 1.5 x $10^5$ in N2B27 medium on gelatinized plates. The medium was changed every other day and at day 8, the cells were fixed and stained with anti-beta tubulin III (Tuj1) antibody (R&D Systems).

**Chimeras**

Chimeras were produced by a standard microinjection protocol. Chimerism was estimated based on coat colour since iPSCs derived from EpiSCs and MEFs are of MF1 and C57BL/6J genetic background (agouti and black furs) whereas the host blastocysts were from albino C57BL/6.

**Statistical analyses**

All statistical analyses were performed in GraphPad Prism (Version 6.01). Data are presented as means ± standard deviations. Statistical significance was determined either using a Student's unpaired *t*-test with two-tailed distribution or Two-way ANOVA. Students' t-test was used for comparison across two groups while Two-way ANOVA with multiple comparisons were performed on samples undergoing a time course experiment. *p*-values less than 0.05 were considered significant.

## Supplementary Figure Legends

**Supplementary Figure S1 | Related to main Figure 1: Plasmids used in the study**

**LTR:** long terminal repeat from HIV-1. **PB:** *PiggyBac* inverted terminal repeats to enable stable integration by *PBase* mediated transposition. **U6:** U6 promoter for sgRNA transcription. **sgRNA:** sgRNA scaffold. **sgRNA-MS2-MS2:** sgRNA scaffold with extended stem loops for dCas9:SAM. **PGK:** PGK promoter. **PuroR:** puromycin N-acetyltransferase. **T2A:** 2A peptide from *Thosea asigna* virus capsid protein. **BFP:** blue fluorescent protein. **Poly(A):** polyadenylation signal. **EF1-α:** human elongation factor 1 alpha promoter. **VP160-dCas9:** 10 tandem repeats of transcriptional activation domain of herpes simplex virus protein VP16 fused to dCas9. **mCherry:** mCherry fluorescent protein. **P2A:** 2A peptide from *porcine teschovirus-1* polyprotein. **BlastR:** *Aspergillus terreus* blasticidin S deaminase. **GCN4:** GCN4 single chain antibody. **VP64:** 4 tandem repeats of transcriptional activation domain of herpes simplex virus protein VP16. **dCas9-SunTag:** GCN4 peptide fused to dCas9. **dCas9-VPR:** VP64, P65 and RTA (transcriptional activation domain from the human herpesvirus 4) fused to dCas9. **MS2:** bacteriophage MS2 coat protein. **P65:** C-terminal portion of the p65 subunit of mouse NF-κB. **HSF1:** C-terminal activation domain from the human heat shock transcription factor. **E2A:** 2A peptide from equine rhinitis A virus polyprotein. **bpA:** bovine growth hormone polyadenylation signal. **CAG:** CMV enhancer, chicken β-actin and rabbit β-globin promoter. **TRE:** tet response element. **TET3G:** Tet-On-3G transactivator protein.

**Supplementary Figure S2 | Related to main Figures 1 and 2: Supplementary results for GoF CRISPRa screen and gene dosage of *Oct4***

**(a)** Flow cytometry plots showing dynamics of GFP expression in *Oct4*-GFP reporter EpiSCs during reprogramming: *Oct4*-GFP EpiSCs transfected with dCas9:SAM +

sgRNA against gene *Nanog*, dCas9:SAM only or untransfected. Over a time course of 14 days in selective 2i/LIF medium, the GFP positive cell population rapidly and completely disappears in the untransfected or dCas9:SAM only groups. However, cells reprogrammed by CRISPRa mediated overexpression of *Nanog* recover their *Oct4*-GFP levels by day 14. **(b)** Flow cytometry plots showing the percentage of BFP$^{+ve}$ cells obtained upon transducing *Oct4*-GFP EpiSCs with lentiviral sgRNA library at an MOI of 0.0 (untransduced) and an MOI of 0.3. **(c)** RT-qPCR on *Oct4*-GFP EpiSCs transfected with CRISPRa and cDNA Oct4 showing levels of total *Oct4* in cells after 3 days in 2i/LIF. Levels of *Oct4* are normalized to *Gapdh* (error bars represent mean of experimental triplicates ± s.d.). **(d)** Western blot on *Oct4*-GFP EpiSCs transfected with CRISPRa and cDNA *Oct4* showing levels of total Oct4 in cells after 3 days in 2i/LIF (top panel). Western blot for Oct4 in gRNA and CAG cDNA transfected EpiSCs with a comparison to ESCs (bottom panel) *Gapdh* was used as loading control in both cases. **(e)** Variable gene dosage with tiled guides: *Oct4*-GFP EpiSCs were transfected with dCas9:SAM and all tiled sgRNAs for *Sall1* and *Nanog* available in our library. Numbers on top of bars indicate distance to the transcription start site (TSS). RT-qPCR expression levels of *Sall1* and *Nanog*, on tiled sgRNA transfected cells, normalized to *Gapdh* and relative to dCas9:SAM (error bars represent mean of experimental triplicates ± s.d.). **(f)** *Umodl1* regulates reprogramming pathways: RT-qPCR expression levels of key regulators in JAK/STAT3 and TGFβ signalling, on flow-sorted for sgRNA expression either 24 h after transfection (EpiSC media) or 24 h after changing to 2i/LIF media (48 h after transfection), normalized to *Gapdh* and relative to dCas9:SAM (error bars represent mean of 3 independent experiments ± s.d.), ** $p<0.01$, *** $p<0.001$. **(g)** RT-qPCR shows that *Sall4* is not overexpressed when

*Oct4*-GFP+ve EpiSC are transfected with *Sall1* CRISPRa relative to dCas9:SAM (error bars represent mean of 3 independent experiments ± s.d.).

**Supplementary Figure S3 | Related to main Figure 2: Reprogramming in *Nanog*-GFP reporter EpiSCs and supplementary results for ESC to EpiSC conversion**

**(a,b)** Gene induction of *Sall1*, *Nanog* and *Oct4* in *Oct4*-GFP EpiSC **(a)** and *Nanog*-GFP EpiSCs **(b)**, respectively, with CRISPRa (single sgRNA per target) as well as cDNA mediated overexpression. Expression levels were measured by RT-qPCR 72h after transfection and expressed in relation to dCas9:SAM-only or empty vector (PBCAG:Empty) (error bars represent mean of 3 independent experiments ± s.d). **(c)** Reprogramming efficiencies of *Sall1*, *Nanog* and *Oct4*. *Nanog*-GFP EpiSCs were stably transfected with CRISPRa or cDNA and cultured in 2i/LIF for 16 days without selection. *Nanog*-GFP$^{+ve}$ colonies were counted and are represented as mean of 3 independent experiments ± s.d ** p<0.01. **(d)** RT-qPCR expression profiles of pluripotency markers and EpiSC markers in iPSC colonies normalized to *Gapdh* and relative to EpiSCs (error bars represent mean of 3 independent experiments ± s.d). Cells were transfected with cDNA as before. **(e)** Chimeric mouse produced with cDNA *Sall1* – induced PSCs injected into C57B/6 blastocyst. **(f)** Identical endpoints of *Oct4*-GFP and *Nanog*-GFP reporter EpiSCs in reprogramming: *Oct4*-GFP EpiSCs (top panel) and *Nanog*-GFP EpiSCs (bottom panel) transfected with either dCas9:SAM alone or in combination with sgRNAs against *Sall1*, *Nanog* and *Oct4* and selected in 2i/LIF medium in a time course of 14 and 22 days. As before, *Oct4*-GFP EpiSCs lose initial GFP expression rapidly and recover it upon successful reprogramming via CRISPRa mediated gene induction of *Sall1*, *Nanog* or *Oct4*, but not in cells transfected with dCas9:SAM only. *Nanog*-GFP reporter EpiSCs – showing no baseline GFP expression – nevertheless upregulate GFP with similar but slightly slower dynamics in

sgRNA transfected groups only. On day 14 both reporter lines show comparable GFP positive cell populations and a clear synergistic effect when co-transfecting sgRNAs against *Sall1* and *Nanog*. Values are mean of 3 independent experiments ± s.d ** $p<0.01$, *** $p<0.001$, **** $p<0.0001$ **(g-i)** *Rex1*-GFP$^{+ve}$ ES cells were stably transfected with *Sall1* or *Nanog* cDNA, or empty vector and cultured in EpiSC medium. Change of expression levels of pluripotency marker *Rex1* and differentiation markers *Fgf5* and *Otx2* measured by RT-qPCR normalized to *Gapdh* expression (error bars represent mean of 3 independent experiments ± s.d).

**Supplementary Figure S4 | Related to main Figures 3 and 4: *Sall1* cannot replace *Oct4* in MEF reprogramming and supplementary results for RNA-seq**

**(a)** (4F+CRISPRa) MEFs were stably transfected with CAG4F and gRNAs against *Sall1*/*Nanog*/*Sall1*+*Nanog* and reprogrammed in ESC medium. AP$^{+ve}$ colonies were counted after 18 days of reprogramming. Gene induction of *Sall1* produced more iPSC colonies compared to CAG4F alone and gene induction of both *Sall1* and *Nanog* produced significantly higher number of colonies compared to activation of either *Sall1* or *Nanog* alone (error bars represent mean of 3 independent experiments ± s.d., ** $p<0.01$). (4F+cDNA) MEFs stably transfected with TRE4F, TRENanog and TRESall1 (all co-transfected with PBEF-1αTet3G) and induced with 0.5 μg/ml Doxycycline for 12 days. Overexpression of *Sall1* produced more iPSC colonies compared to CAG4F alone and overexpression of both *Sall1* and *Nanog* produced significantly higher number of colonies compared to activation of either *Sall1* or *Nanog* alone. AP$^{+ve}$ colonies were counted on day 18 (error bars represent mean of 3 independent experiments ± s.d., *** $p<0.001$). **(b)** Morphology of *Oct4*-GFP$^{+ve}$ colonies at day 18 in ESC media. The reprogrammed colonies from *Oct4*-GFP$^{+ve}$ MEFs are morphologically similar to ESC colonies with *Oct4*-GFP$^{+ve}$ fluorescence. No

colonies were observed in untransfected or dCas9:SAM only transfected MEFs. **(c)** MEF were stably transfected with CAG4F and TRESall1/PBEF-1αTet3G and reprogrammed in ESC medium. 24h after transfection, expression of Sall1 was induced with different concentration of Doxycycline for 12 days. After 18 days, both *Oct4*-GFP$^{+ve}$ and AP$^{+ve}$ colonies were counted. Overexpression of *Sall1* produced more iPSC colonies (error bars represent mean of 3 independent experiments ± s.d., ** $p<0.01$, *** $p<0.001$, $^{\Delta}$ $p<0.05$, $^{\Delta\Delta}$ $p<0.01$ vs. 4F). **(d)** MEFs stably transfected with CAG4F and TRESall1/PBEF-1αTet3G and reprogrammed in ESC medium. 0.5 μg/ml Doxycycline was added on the indicated days for the duration of reprogramming. *Oct4*-GFP$^{+ve}$ and AP$^{+ve}$ colonies were counted on D18 (error bars represent mean of 3 independent experiments ± s.d., ** $p<0.01$, $^{\Delta\Delta\Delta}$ $p<0.001$ vs. 4F). **(e-g)** MEFs were stably transfected with combinations of *Oct4*, *C-myc*, *Klf4*, *Sox2* (OCKS) and *Sall1* cDNA, whereby *Sall1* replaced either **(e)** *Klf4*, **(f)** *Sox2* or **(g)** *Oct4*. Cells were reprogrammed in ESC medium for 18 days and AP$^{+ve}$ colonies were counted (error bars represent mean of 3 independent experiments ± s.d). **(h)** Venn diagram showing number and percentage of genes being differentially expressed in *Oct4*-GFP EpiSC transfected with cDNA for *Sall1/Nanog/Sall1+Nanog* for RNA-seq. Differentially expressed genes were identified with a cut-off padj value < 0.001. **(i)** CAG cDNA mediated overexpression of *Sall1*, *Nanog* in *Oct4*-GFP EpiSC transfected with either *Sall1*/*Nanog*/*Sall1+Nanog* for RNA-seq. Expression levels were measured by RT-qPCR 48h after transfection and expressed in relation to empty vector (error bars represent mean of 3 independent experiments ± s.d).

SUPPLEMENTARY FIGURE S1

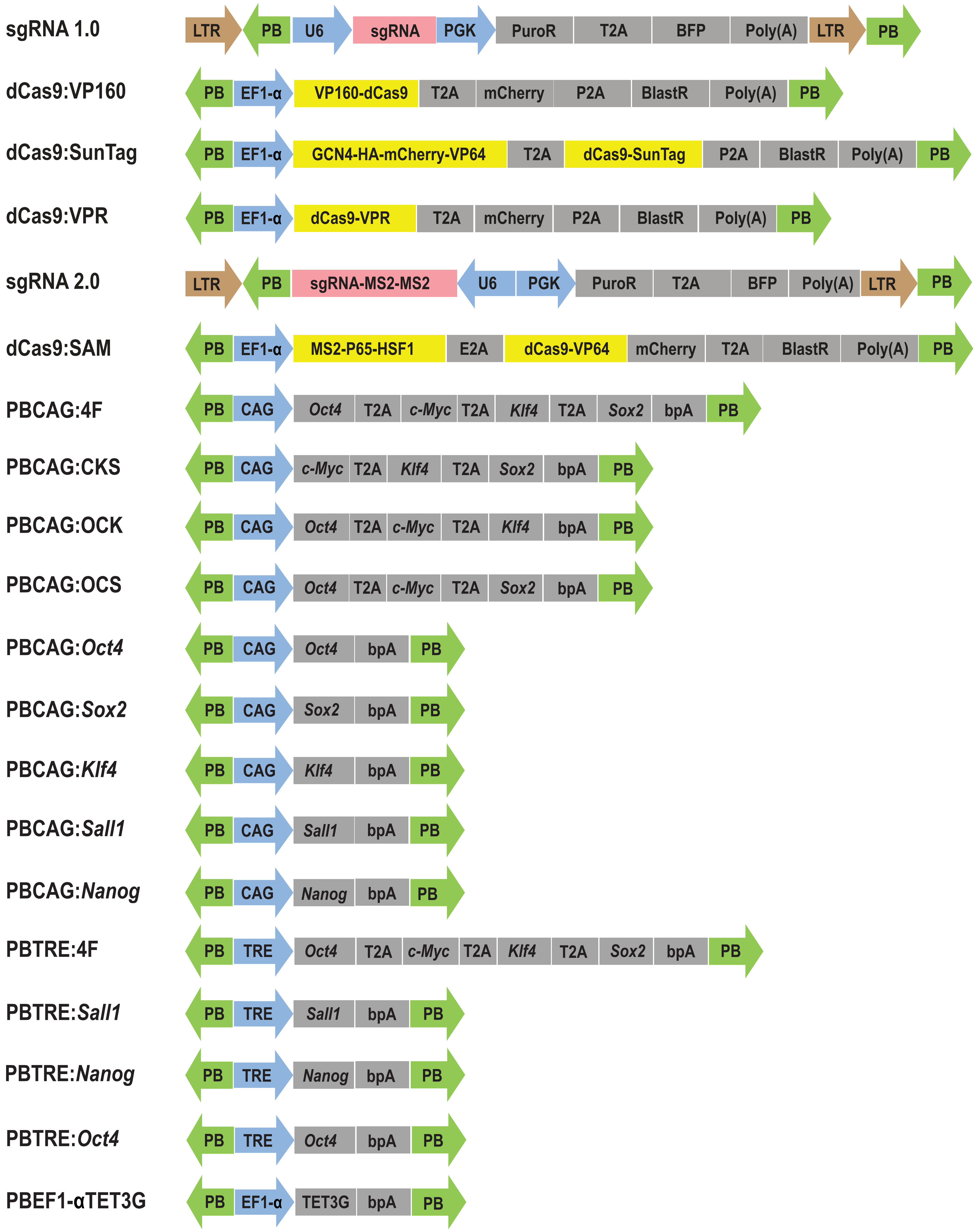

SUPPLEMENTARY FIGURE S2

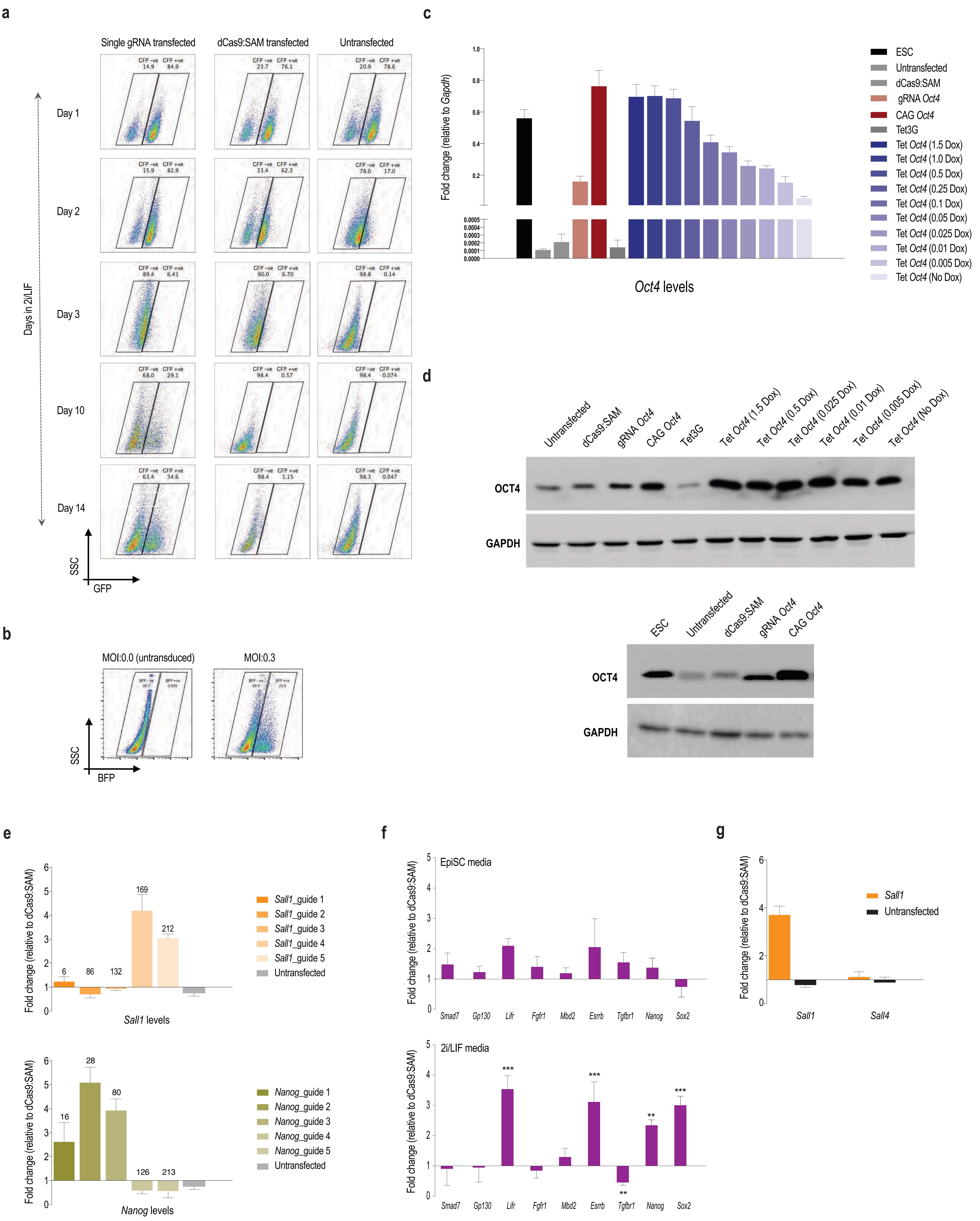

SUPPLEMENTARY FIGURE S3

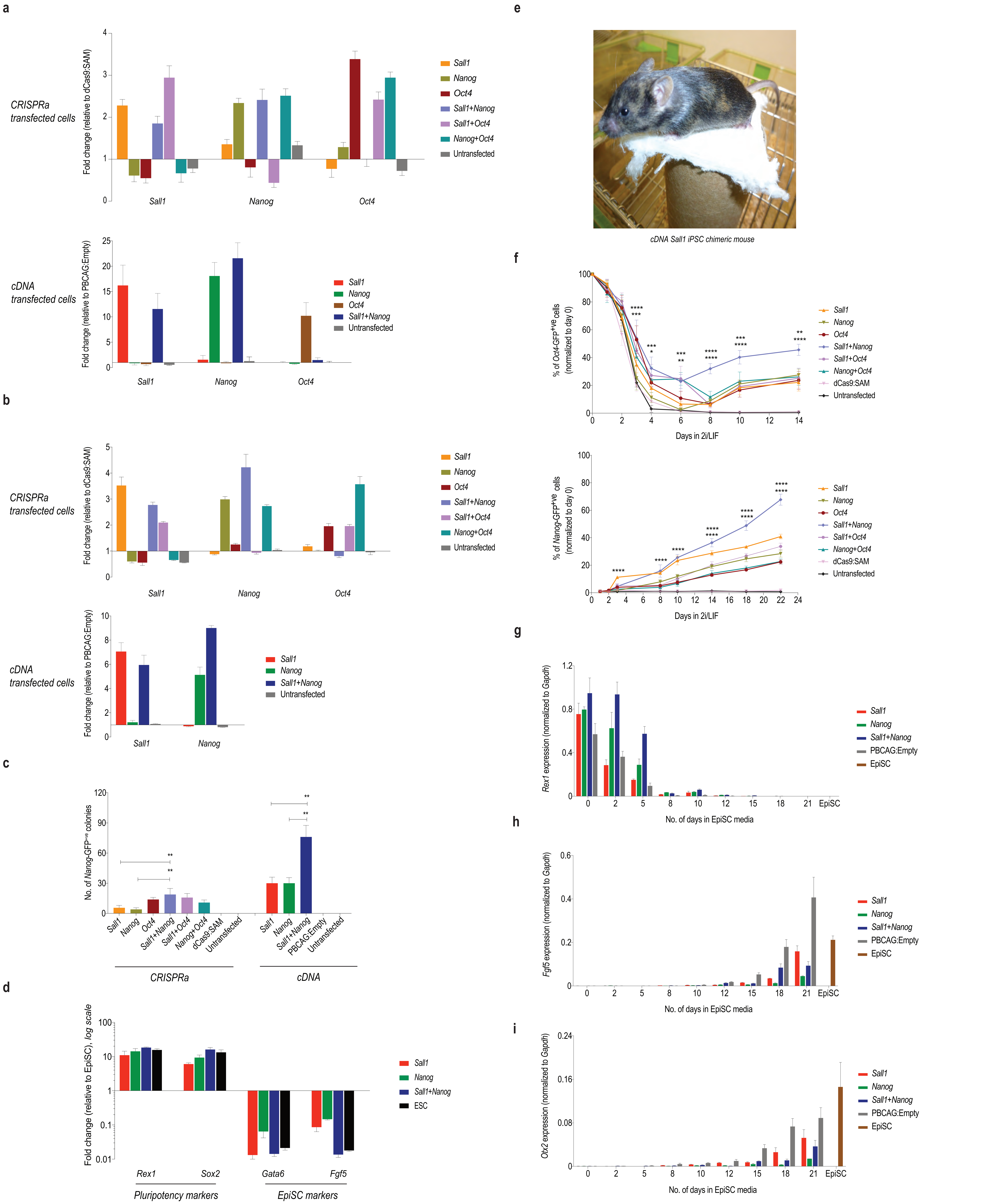

SUPPLEMENTARY FIGURE S4

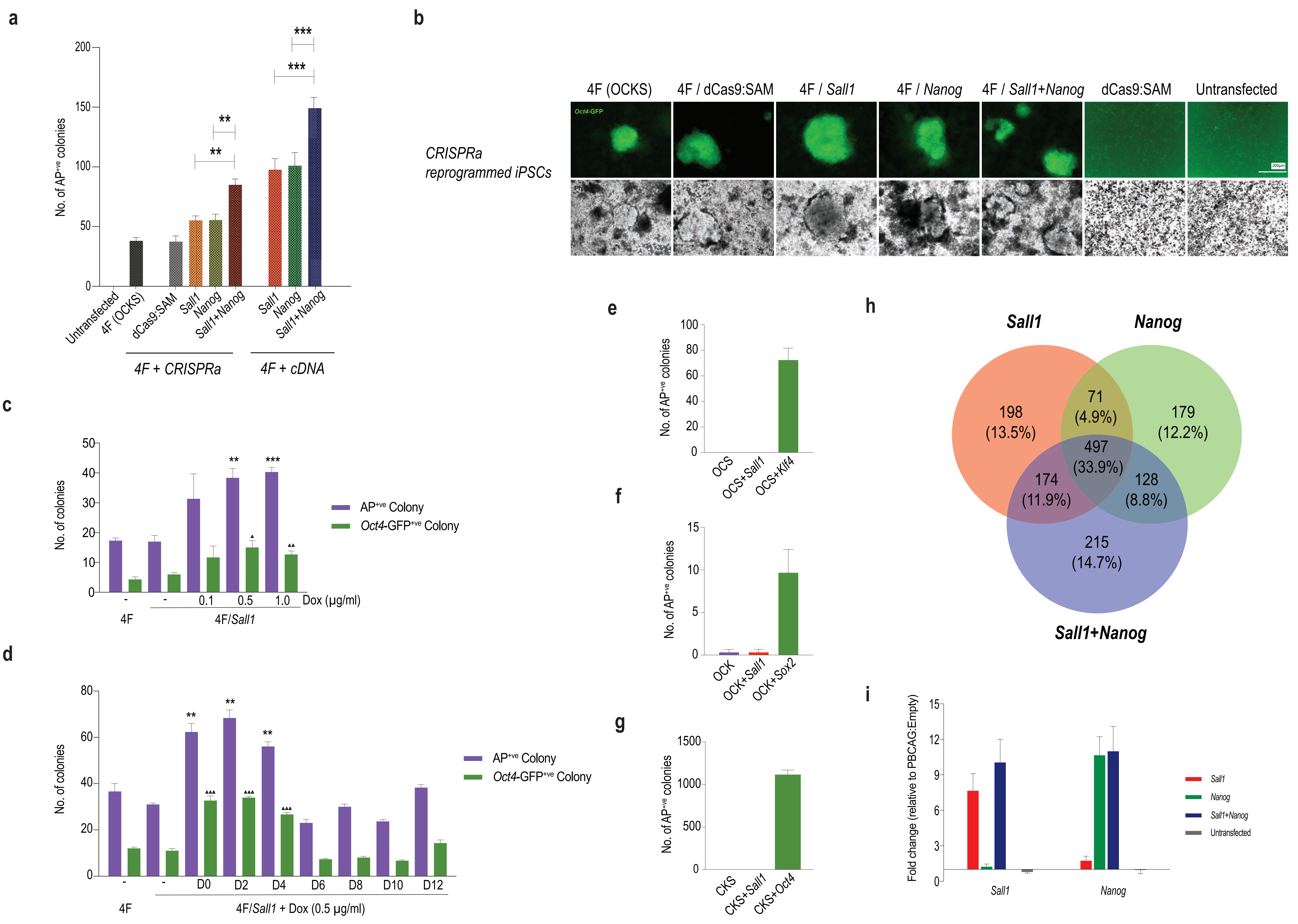

**Supplementary Table S1**

**Primers for confirming *PiggyBac* mediated Gene integration (Genotyping).**

| Gene Name | Primer Name | Primer Sequence |
|---|---|---|
| *Sall1-BPA* | Sall1-BPA F | CAATCCTGTCAAGTTCCCAGAAAT |
| | Sall1-BPA R | CATCCCCAGCATGCCTGCTATT |
| *Nanog-BPA* | Nanog-BPA F | AGGGCTATCTGGTGAACGCATC |
| | Nanog-BPA R | AATCCTCCCCCTTGCTGTCCT |
| *Oct4-c-Myc* | Oct4-c-Myc F | GCCCCCAGGTCCCCACTTTG |
| | Oct4-c-Myc R | CCAGCTGATCGGCGGTGGAG |
| *Klf4-Sox2* | Klf4-Sox2 F | ACTATGCAGGCTGTGGCAA |
| | Klf4-Sox2 R | TTGCTGCGGGCCCGGCGGCT |
| *Tet3G* | Tet3G F | CCGTCCAGGCACCTCGATTAGTTC |
| | Tet3G R | GGTATGACTTGGCGTTGTTCC |
| *Actb* | Actb F | GTTTGAGACCTTCAACACCCC |
| | Actb R | GTGGCCATCTCCTGCTCGAAGTC |
| *gRNA* | pKLV_Flip_gRNA F | AGCAAAAAAAGCACCGACTCG |
| | pKLV_Flip_gRNA R | TAAAGCGCATGCTCCAGACTGC |
| *dCas9:SAM* | SamCas9 F | TTACTCAGTTCGTGCTCGTGGAC |
| | SamCas9 R | ATTGCCTTCACGATGAGTTCACA |

**Supplementary Table S2**

**Combinations of cDNA transfection used for MEF reprogramming.** ***(Related to Figure 3 and Supplementary Figure S4)***

| **Constructs** | **Combinations (amount of DNA in µg)** | | | | | | | | | | | | | | |
|---|---|---|---|---|---|---|---|---|---|---|---|---|---|---|---|
| | 1 | 2 | 3 | 4 | 5 | 6 | 7 | 8 | 9 | 10 | 11 | 12 | 13 | 14 | 15 |
| PBCAG4F | 1.0 | 1.0 | | | | | | | | | | | | | |
| PBCAGCKS | | | | | | | 1.0 | | | 1.0 | | | 1.0 | | |
| PBCAGOCK | | | | | | | | 1.0 | | | 1.0 | | | 1.0 | |
| PBCAGOCS | | | | | | | | | 1.0 | | | 1.0 | | | 1.0 |
| PBCAG*Oct4* | | | | | | | | | | 1.0 | | | | | |
| PBCAG*Klf4* | | | | | | | | | | | | 1.0 | | | |
| PBCAG*Sox2* | | | | | | | | | | | 0.5 | | | | |
| PBTRE4F | | | 1.0 | 1.0 | 1.0 | 1.0 | | | | | | | | | |
| PBTRE*Sall1* | | 0.5 | | 0.5 | | 0.5 | | | | | | | 0.5 | 0.5 | 0.5 |
| PBTRE*Nanog* | | | | | 0.1 | 0.1 | | | | | | | | | |
| PBEF1-αTET3G | | 1.0 | 1.0 | 1.0 | 1.0 | 1.0 | | | | | | | 1.0 | 1.0 | 1.0 |
| PiggyBac Transposase | 2.0 | 2.0 | 2.0 | 2.0 | 2.0 | 2.0 | 2.0 | 2.0 | 2.0 | 2.0 | 2.0 | 2.0 | 2.0 | 2.0 | 2.0 |

## Supplementary Table S3

**Mouse RT-qPCR Probes for RNA Expression.** ***(Related to Figures 2, 4 and Supplementary Figures S2, S3 and S4)***

| Gene Name | Applied Biosystems Catalogue Number |
|---|---|
| *Esrrb* | Mm00442411_m1 |
| *Fgf5* | Mm00438615_m1 |
| *Gapdh* | 4352339E |
| *Gata6* | Mm00802636_m1 |
| *Id3* | Mm00492575_m1 |
| *Klf4* | Mm00516104_m1 |
| *Nanog* | Mm02384862_g1 |
| *Pou5f1* | Mm00658129_gH |
| *Rex1* | Mm03053975_g |
| *Sox2* | Mm03053810_s1 |
| *Id2* | Mm00711781_m1 |
| *Mdb2* | Mm00521967_m1 |
| *Jarid2* | Mm00445574_m1 |
| *Tet3* | Mm00805756_m1 |
| *Tex10* | Mm06549480_m1 |
| *Klf5* | Mm00456521_m1 |
| *Smad7* | Mm00484742_m1 |
| *Gp130* | Mm00439665_m1 |
| *Sall1* | Mm00491266_m1 |
| *Otx2* | Mm0046859_m1 |
| *Fgfr1* | Mm00438930_m1 |
| *Fam189a2* | Mm01194369_m1 |
| *Lifr* | Mm00442942_m1 |
| *Myc* | Mm00487804_m1 |
| *Mycn* | Mm00476449_m1 |
| *Arid2* | Mm00558381_m1 |

## Supplementary Table S4

**Antibodies for Western Blotting / Immunofluorescence staining.** ***(Related to Figure 3 and Supplementary Figure S2)***

| Antibody | Company | Catalogue Number | Dilution |
|---|---|---|---|
| SSEA-1 Clone MC480 | BD Pharmingen | 560079 | 1:200 |
| NANOG | Abcam | Ab80892 | 1:150 |
| β III TUBULIN (TUJ1) | R & D Systems | MAB1159 | 1:150 |
| α-Smooth Muscle Actin (SMA) | R & D Systems | MAB1420 | 1:150 |
| α-Fetoprotein (AFP) | R & D Systems | MAB1368 | 1:150 |
| H3K27ME3 | Millipore | 07-449 | 1:1000 |
| OCT4 (C10) | Santa-Cruz | SC-5279 | 1:150 (IF)<br>1:800 (WB) |
| GAPDH | Sigma | G8795 | 1:4000 |
